# Sex-specific efficacy and safety of short-term and de-escalation DAPT strategies after PCI: a network meta-analysis

**DOI:** 10.1186/s13293-026-00903-y

**Published:** 2026-04-22

**Authors:** Hao-Yun Lo, Su-Kiat Chua, Jen-Kuang Lee, Donna Shu-Han Lin

**Affiliations:** 1 Division of Cardiology, Department of Internal Medicine, Shin Kong Wu Ho- Su Memorial Hospital, No. 95, Wen Chang Road, Shih Lin District, Taipei, 111 Taiwan; 2 School of Medicine, College of Medicine, Fu Jen Catholic University, New Taipei City, Taiwan; 3 Division of Cardiology, Department of Internal Medicine, National Taiwan University Hospital, Taipei, Taiwan; 4 Department of Internal Medicine, National Taiwan University College of Medicine, Taipei, Taiwan; 5 Department of Laboratory Medicine, National Taiwan University College of Medicine, Taipei, Taiwan; 6 Cardiovascular Center, National Taiwan University Hospital, Taipei, Taiwan; 7 Telehealth Center, National Taiwan University Hospital, Taipei, Taiwan

**Keywords:** Antiplatelet strategies, Percutaneous coronary intervention, De-escalation, Short DAPT, Sex differences

## Abstract

**Background:**

Sex differences in thrombotic and bleeding risks after percutaneous coronary intervention (PCI) are well established, yet it remains uncertain whether the efficacy and safety of modern dual antiplatelet therapy (DAPT) strategies differ between men and women.

**Objectives:**

This study aimed to compare sex-specific outcomes of contemporary short-term and de-escalation DAPT strategies to guide individualized post-PCI management.

**Methods:**

A network meta-analysis of randomized controlled trials reporting sex-stratified outcomes for alternative DAPT strategies versus standard DAPT was performed. Strategies were categorized as standard DAPT, guided de-escalation, short DAPT followed by aspirin or P2Y12 inhibitor (P2Y12i) monotherapy, de-escalation to clopidogrel, or de-escalation to reduced-dose P2Y12i. The primary outcomes were major adverse cardiovascular events (MACE), bleeding, and net adverse clinical events (NACE).

**Results:**

A total of 25 trials including 115,223 men and 38,574 women were analyzed. For MACE, no strategy was superior in men, while de-escalation to clopidogrel showed a favorable trend in women. In men, short DAPT followed by aspirin (risk ratio [RR] 0.44, 95% confidence interval [CI] 0.27–0.73) or P2Y12 inhibitor monotherapy (RR 0.52, 95% CI 0.39–0.70) reduced bleeding, whereas this benefit was not observed in women (P for sex difference = 0.015). De-escalation to clopidogrel provided the lowest bleeding risk and the most favorable NACE profile in women.

**Conclusions:**

Sex-based differences exist in the optimal DAPT strategy following PCI, with short DAPT followed by monotherapy robustly reducing bleeding risk in men, while de-escalation to clopidogrel showed the most favorable profile for bleeding and net clinical outcomes in women. However, this finding should be considered hypothesis-generating pending confirmatory evidence, and underscores the need for dedicated prospective trials evaluating sex-specific antiplatelet strategies after PCI.

**Supplementary Information:**

The online version contains supplementary material available at 10.1186/s13293-026-00903-y.

## Abstract (plain language summary)

After undergoing a procedure to place a stent for heart disease, patients are prescribed two types of blood-thinning medications to prevent dangerous blood clots. While this treatment is life-saving, it increases the risk of bleeding. Historically, medical guidelines have recommended similar treatment plans for everyone. However, men and women are biologically different; women generally have a higher risk of bleeding, and their blood cells may respond differently to these medications.

Our study aimed to find out if men and women should receive different treatments to balance safety and effectiveness. We combined and analyzed data from 25 clinical trials involving over 150,000 patients. We compared the standard treatment against newer strategies, such as shortening the time patients take two drugs or switching to a milder medication.

We found that a “one-size-fits-all” approach is not ideal. The results showed that the best strategy depends on the patient’s sex. For men, the safest option was to stop one of the blood thinners early and continue with a single, potent medication. For women, however, the best balance of safety and protection came from “de-escalation”—switching from a strong blood thinner to a gentler, standard medication (clopidogrel).

This research is important because it suggests that doctors should customize blood-thinning prescriptions based on whether the patient is male or female. By tailoring treatment in this way, we can better protect women from bleeding complications without increasing their risk of heart problems.

**Supplementary Information:**

The online version contains supplementary material available at 10.1186/s13293-026-00903-y.

## Highlights

- This network meta-analysis of over 150,000 patients is the first to demonstrate that the safety benefits of modern dual antiplatelet therapy (DAPT) strategies differ significantly by sex, with short-term DAPT reducing bleeding in men but not in women.
- De-escalation to clopidogrel was identified as the most favorable strategy for women, providing the lowest bleeding risk and the best overall net adverse clinical event profile.
- These findings challenge a "one-size-fits-all" approach and support tailoring post-PCI antiplatelet therapy by sex, specifically favoring de-escalation to clopidogrel for women and short DAPT followed by P2Y12 inhibitor monotherapy for men.

**Supplementary Information:**

The online version contains supplementary material available at 10.1186/s13293-026-00903-y.

## Introduction

Dual antiplatelet therapy (DAPT), consisting of aspirin and a P2Y12 inhibitor (P2Y12i), remains the cornerstone of treatment following percutaneous coronary intervention (PCI) with drug-eluting stents, recommended for 6 months in patients with chronic coronary syndrome (CCS) and 12 months for acute coronary syndrome (ACS) [1–3]. Despite this, advancements in stent technology have raised questions about the necessity for prolonged intensive antiplatelet therapy, especially considering the balance between reducing thrombotic events and minimizing bleeding risk. Consequently, strategies involving shorter durations of DAPT or subsequent monotherapy with either aspirin or a P2Y12i have emerged as viable alternatives [4].

However, a critical gap remains in understanding whether sex differences affect the efficacy and safety outcomes of these antiplatelet strategies. Evidence suggests distinct thrombotic and bleeding risk profiles between male and female patients undergoing PCI. Women typically present with smaller coronary vessels and may have different plaque characteristics compared to men [5, 6]. A recent prospective multicenter study found that young women (< 55 years old) with ST-segment elevation myocardial infarction (STEMI) had thrombi significantly lower in fibrin, p-selectin, and von Willebrand factor content compared to young men, suggesting potential differences in the pathophysiological mechanisms of coronary thrombosis by sex [7]. Furthermore, ex vivo and clinical studies indicate women exhibit higher platelet reactivity after aspirin administration compared to men, potentially influencing their response to antiplatelet therapy [8]. Additionally, it has been shown that the reduction in platelet reactivity achieved with the newer ADP antagonists was less pronounced in women compared to men [9], suggesting sex-specific differences in platelet physiology.

In addition to the aforementioned findings from studies examining platelet biology and thrombus composition, contemporary clinical data reveal sex-specific differences in the prognosis of STEMI patients, with women experiencing higher short-term mortality despite adjustments for baseline cardiovascular risk factors [10]. Women are also at higher bleeding risk following PCI, a disparity not fully attributable to demographic or social factors alone, suggesting inherent biological differences may significantly contribute to increased bleeding events [11, 12]. Data from the HORIZONS-AMI trial demonstrated that female sex independently predicted major bleeding at 3 years post-PCI [11]. Similarly, analysis of the TRANSLATE-ACS study indicated higher rates of bleeding in women post-PCI, particularly moderate or severe bleeding events, even after extensive adjustment for clinical covariates [13]. The MASTER DAPT trial further highlighted that female patients with high bleeding risk receiving abbreviated DAPT demonstrated a favorable trend toward reduced major adverse cardiac and cerebral events compared to men, who did not experience a similar benefit [14].

Given these findings, it is essential to clarify the optimal antiplatelet regimen for female patients undergoing PCI. To date, there is limited evidence directly addressing sex differences in outcomes following various DAPT strategies, particularly those involving short-term therapy and de-escalation regimens. This meta-analysis thus aims to address this knowledge gap by comprehensively comparing clinical outcomes between sexes across different DAPT durations and de-escalation approaches, ultimately guiding sex-specific antiplatelet therapy decisions for improved patient care.

## Materials and methods

The protocol for this meta-analysis was registered with the International Prospective Register of Systematic Reviews (PROSPERO), under registration number CRD42024559223. This study followed the Preferred Reporting Items for Systematic Reviews and Meta-Analyses (PRISMA) guidelines for both execution and reporting. Ethical approval and informed consent were not necessary, as the analysis was based exclusively on previously published, publicly available, and anonymized data.

### Search strategy

A comprehensive literature search was performed using PubMed, EMBASE, and the Cochrane databases to identify relevant studies published between January 1, 2009, and September 30, 2024, comparing various antiplatelet therapy strategies following percutaneous coronary intervention (PCI). The search terms included “antiplatelet therapy,” “de-escalation,” “switch,” “platelet function monitoring,” “genotype-guided,” “P2Y12i monotherapy,” “ticagrelor monotherapy,” “prasugrel monotherapy,” “aspirin monotherapy,” “ticagrelor,” “prasugrel,” “clopidogrel,” “aspirin,” “acetylsalicylic acid,” “coronary intervention,” “angioplasty,” and “stent”. The details of the search strategy used are provided in the Supplemental Table 1. Studies were considered eligible for inclusion if they met the following criteria: [1] published in English; [2] randomized controlled trials (RCTs) comparing alternative antiplatelet strategies with standard DAPT after PCI; [3] reported subgroup analyses separately for male and female patients; and [4] provided outcome data on major adverse cardiovascular events (MACE), Bleeding Academic Research Consortium (BARC) 2,3,5 bleeding, or net adverse clinical events (NACE). Secondary analyses of relevant RCTs were also evaluated for inclusion. Abstracts, letters, review articles, meta-analyses, and gray literature were excluded from the analysis. Initial screening of search results was independently conducted by two authors (DSHL and HYL) to assess their relevance to the study objectives. Subsequently, full-text reviews were independently carried out by two authors to confirm eligibility for final inclusion. Any disagreements during the selection process were resolved by discussion and reached consensus.

### Outcomes definition

This study assesses both efficacy and safety outcomes. The primary efficacy outcome is MACE, defined as a composite of all-cause death, cardiac death, myocardial infarction, ischemic stroke, definite stent thrombosis, or clinically driven target vessel revascularization. Although specific definitions of primary thrombotic events vary slightly across the included trials, all are collectively categorized as MACE for the purpose of this analysis. Detailed outcome definitions for each trial are provided in Supplemental Table 2. The primary safety outcome was defined as bleeding events categorized as BARC types 2, 3, or 5 [15]. Furthermore, NACE—defined as a composite of MACE and bleeding events, whichever occurred first—was evaluated as an outcome of interest in this analysis using the prespecified composite endpoint data reported in each trial. Across trials, outcomes were consistently reported as time to first event (i.e., first-event analyses).

### Data extraction

Two authors (DSHL and HYL) evaluated the risk of bias in each included RCT using the Cochrane Risk of Bias tool. Data extraction was standardized and encompassed essential details such as the trial name, publication year, specific antiplatelet strategies employed in the intervention group, numbers of male and female participants in both intervention and control arms, definitions of reported outcomes, event counts separated by sex, and the reported risk ratios (e.g., relative risk or hazard ratio). For the extraction of outcome data, we primarily used the sample size and the number of events in each arm to calculate the relative risk and the standard error of the estimate. In a few instances, however, we extracted the reported risk ratios—typically hazard ratios—that had been calculated and presented by the original study authors.

### Antiplatelet therapy strategies

To streamline comparative analyses, antiplatelet therapy strategies were classified into six distinct categories: [1] Standard dual antiplatelet therapy (DAPT); [2] Guided de-escalation; [3] Short-duration DAPT followed by aspirin monotherapy; [4] Short-duration DAPT followed by P2Y12i monotherapy; [5] De-escalation specifically to clopidogrel; and [6] De-escalation involving a reduced-dose P2Y12i. Details of each trial’s categorization are presented in Table 1. Network plots stratified by outcome and sex, with node sizes and edge thickness reflecting sample size and comparison weight, are provided in the supplements (Supplemental Fig. 1A-1 C).

**Table 1:** Study-level characteristics of included randomized controlled trials

Trial name	Comparison	No. of participants	Follow-up, mo	Mean age, yrs	Female, %	HTN, %	HLD, %	DM, %	CKD*, %	Current smoking, %	Previous MI, %	Previous PCI, %	Previous CABG, %	Silent ischemia, %	Stable angina, %	STEMI, %	NSTEMI, %	Unstable angina, %
PRODIGY	6-mo DAPT (C), then A only	983	24	67.9	24	70.4	53.4	23.7	NA	25.1	26.2	17.7	10.7	0	25.4	33.3	22.8	18.5
	24-mo DAPT (C)	987	24	67.8	22.6	73.0	56.0	24.7	NA	22.5	27.3	18.6	11.1	0	26.0	32.5	22.9	18.5
RESET	3-mo DAPT (C), then A only	1059	12	62.4	35.6	62.3	57.7	29.8	NA	25.2	1.8	3.5	0.2	0	44.5	14.7		40.8
	12-mo DAPT (C)	1058	12	62.4	37.1	61.4	59.9	28.8	NA	22.8	1.6	3.0	0.6	0	46.3	13.8		39.9
OPTIMIZE	3-mo DAPT (C), then A only	1563	12	61.3	36.5	86.4	63.2	35.4	7.4†	18.6	34.6	20.9	7.1	8.6	59.8	0	5.4	26.2
	12-mo DAPT (C)	1556	12	61.9	36.9	88.2	63.7	35.3	5.8†	17.3	34.8	19.1	8.2	9.2	58.6	0	5.4	26.9
ISAR-SAFE	6-mo DAPT (C), then A only	1997	9‡	67.2	19.3	90.1	87.5	24.8	NA	14.6	25.9	NA	7.7	10.9	48.6	7.9	10.4	21.5
	12-mo DAPT (C)	2003	9‡	67.2	19.5	91.5	87.4	24.2	NA	15.3	24.5	NA	7.5	11.3	47.8	8.3	10.1	21.9
I-LOVE-IT 2	6-mo DAPT (C), then A only	909	12	60.4	32.8	61.0	25.3	23.2	NA	36.6	17.2	8.5	0.4	3.0	14.3	13.4	11.3	58.0
	12-mo DAPT (C)	920	12	60.0	31.3	64.8	23.4	22.1	NA	38.3	15.8	6.5	0.4	4.0	15.1	13.7	10.7	56.5
IVUS-XPL	6-mo DAPT (C), then A only	699	12	63	33	63	68	36	NA	25	5	10	3	0	51	15		34
	12-mo DAPT (C)	701	12	64	30	65	65	37	NA	24	4	10	2	0	51	16		33
ANTARCTIC	12-mo DAPT with PFT-guided dosing (P) or de-escalation (P to C)	435	12	80	38	72	53	28	4§	9	19	24	7	0	0	35	46	20
	12-mo DAPT (P 5 mg)	442	12	81	41	72	55	28	8§	9	15	26	5	0	0	34	50	16
NIPPON	6-mo DAPT (C or Ticlopidine), then A only	1654	18	67.4	21.2	71.2	68.3	37.4	NA	58.0	12.2	25.0	1.3	16.2	48.7	12.0	2.0	17.9
	18-mo DAPT (C or Ticlopidine)	1653	18	67.2	20.6	73.1	68.5	38.4	NA	60.3	11.8	26.1	1.8	16.6 (other)	44.4	11.9	1.6	20.0
TROPICAL-ACS	PFT-guided de-escalation	1304	12	59.0	79	61	42	18	3?	45	11	13	3	0	0	56	44	0
	12-mo DAPT (P)	1306	12	58.5	78	62	41	22	3	45	12	14	4	0	0	55	45	0
SMART-DATE	6-mo DAPT, then A only	1357	18	62.0	25.1	49.9	24.2	26.9	1.0	38.0	2.3	4.9		0	0	37.5	31.5	31.0
	12-mo or longer DAPT	1355	18	62.2	24.1	48.7	25.2	28.1	0.5	40.1	1.7	3.9		0	0	37.9	31.4	30.7
REDUCE	3-mo DAPT, then A only	751	12	61.0	17.4	50.7	46.3	21.6	NA	42.1	12.5	11.7	2.8	0	0	49.3	35.6	15.2
	12-mo DAPT	745	12	60.0	22.7	50.7	44.9	19.5	NA	42.7	11.8	9.8	2.8	0	0	45.2	41.0	13.8
GLOBAL LEADERS	1-mo DAPT (T), then 23-mo T only	7980	24	64.5	23.4	73.7	67.0	25.7	13.8	25.9	22.9	32.7	5.6	53.0		13.3	21.1	12.6
	12-mo DAPT (C or T), then 12-mo A only	7988	24	64.6	23.1	73.0	67.9	24.9	13.4	26.3	23.5	32.7	6.2	53.2		12.9	21.1	12.7
SMART-CHOICE	3-mo DAPT then P2Y12i	1495	12	64.6	27.3	61.6	45.1	38.2	2.9	28.4	4.1	11.5		0	41.8	11.0	16.0	31.2
	12-mo DAPT	1498	12	64.4	25.8	61.3	45.5	36.8	3.5	24.5	4.3	11.8		0	41.8	10.0	15.4	32.8
POPular Genetics	CYP2C19 genotype–guided strategy	1242	12	61.9	25.5	41.9	21.0	12.1	9.8	45.8	7.8	8.0	1.0	0	0	100	0	0
	DAPT with T or P	1246	12	61.4	24.8	41.0	20.5	11.1	8.8	45.8	7.0	7.3	1.8	0	0	100	0	0
TWILIGHT	3-mo DAPT (T) then T only	3555	12	65.2	23.8	72.6	60.7	37.1	16.8	20.4	28.7	42.3	10.2	6.6	29.5	0	28.8	35.1
	15-mo DAPT (T)	3564	12	65.1	23.9	72.2	60.2	36.5	16.7	23.1	28.6	42.0	9.8	6.3	28.0	0	30.8	34.9
TICO	3-mo DAPT (T) then T only	1527	12	61	21	50	61	27	19	36	4	9	1	0	0	36	35	29
	12-mo DAPT (T)	1529	12	61	20	51	60	27	22	38	3	8	1	0	0	36	32	32
HOST-REDUCE-POLYTECH-ACS	1-mo DAPT (P) then de-escalate P to 5 mg	1170	12	58.7	10.3	62.6	76.1	43.8	2.6	37.9	3.0	9.7		0	0	14.8	26.3	58.9
	12-mo DAPT (P)	1168	12	58.9	11.2	63.6	77.8	40.9	2.9	33.8	4.7	12.7		0	0	13.1	24.2	62.7
One-Month DAPT	1-mo DAPT then A only	1507	12	67	31	67	81	37	13	17	4	16	1	0	62	3		35
	6 to 12-mo DAPT then A only	1513	12	67	31	66	82	38	14	16	4	18	2	0	59	3		38
TALOS-AMI	1-mo DAPT (T) then 11-mo DAPT (C)	1349	12	60.1	16.1	48.6	41.7	26.8	12.1	49.7	NA	4.5	0.2	0	0	54.4	45.6	0
	12-mo DAPT (T)	1348	12	59.9	17.6	49.2	41.2	27.4	10.9	50.0	NA	4.5	0.1	0	0	53.5	46.5	0
STOPDAPT-2 ACS	1–2 mo DAPT (C or P) then C only	2058	12	67.0	20.8	67.8	66.7	29.5	3.3¥	34.9	6.6	10.9	0.4	0	0	57.3	19.4	23.3
	12-mo DAPT (C)	2078	12	66.6	20.6	68.1	66.9	29.9	3.4¥	33.8	5.3	9.7	0.9	0	0	55.1	20.5	24.4
HOST-IDEA	3 to 6-mo DAPT, then A or C for SIHD, A, C, T or P for ACS	1002	12	65.6	27.0	73.3	81.2	40.5	11.0	NA	4.5	13.2		43.4		0	20.9	35.7
	12-mo DAPT	1011	12	65.9	25.2	73.5	80.1	37.4	10.5	NA	4.2	14.1		46.3		0	19.0	34.7
T-PASS	< 1-mo DAPT (T) then T only	1426	12	61	16	47	74	30	8	39	2	7	< 1	0	0	40	36	24
	12-mo DAPT (T)	1424	12	61	17	48	74	29	7	38	2	7	<!	0	0	41	34	25
MASTER DAPT	1-mo DAPT	2295	12	76.1	30.7	76.9	67.2	32.9	18.2	10.0	18.9	25.9	7.4	10.7	40.2	11.9	25.9	11.3
	At least 3-mo DAPT	2284	12	76.0	30.8	78.2	68.1	34.3	20.1	8.1	18.8	26.0	7.5	12.0	40.6	11.6	24.4	11.4
SHARE	3-mo DAPT, then C for CCS, C or T for ACS	694	12	62.8	22.5	56.7	40.7	32.8	3.3	31.0	5.2	10.7	1.9	26.1		73.9		
	12-mo DAPT	693	12	63.2	25.4	60.5	44.8	35.1	2.5	27.3	5.8	14.7	0.9	26.4		73.6		
ULTIMATE-DAPT	1-mo DAPT (T) then T only	1700	12	62	25.7	62.2	69.3	31.8	7.0	28.6	8.4	10.1	0.1	0	0	28.7	32.1	39.3
	12-mo DAPT (T)	1700	12	63	26.1	62.5	68.1	31.5	7.6	28.4	9.2	10.2	0.2	0	0	27.1	31.2	41.7

A, aspirin; C, clopidogrel; P, prasugrel; T, ticagrelor; mo, months; yrs, years; HTN, hypertension; HLD, hyperlipidemia; DM, diabetes mellitus; CKD, chronic kidney disease; MI, myocardial infarction; PCI, percutaneous coronary intervention; CABG, coronary artery bypass graft surgery; STEMI, ST segment elevation myocardial infarction; NSTEMI, non-ST segment elevation myocardial infarction; DAPT, dual antiplatelet therapy; SIHD, stable ischemic heart disease; ACS, acute coronary syndrome; CCS, chronic coronary syndrome

* All trials defined CKD by an estimated glomerular filtration rate or creatinine clearance of less than 60 ml/min/1.73m^2^ unless otherwise specified

† Defined by a baseline serum creatinine level of 1.5 mg/dL (132.6 µmol/L) or greater

‡ Patients were randomized at 6 months after index procedure and followed up to 15 months after index procedure

§ Defined by a creatinine clearance or less than 30 mL/min

¥ Reported only prevalence of severe chronic kidney disease, defined by an estimated glomerular filtration rate of less than 30 mL/min/1.73 m2 or maintenance dialysis therapy

### Statistical analysis

To evaluate and compare multiple antiplatelet therapy strategies simultaneously within a unified analytical framework, we conducted a network meta-analysis (NMA) using a frequentist graph-theoretical approach. Multivariate meta-analysis was performed using the restricted maximum likelihood (REML) method. Analyses were performed separately for male and female subgroups. Sex-based differences in treatment effects were formally assessed using a network random-effects meta-regression model, in which sex was included as a study-level covariate to explore potential treatment-by-subgroup interactions. To summarize and interpret the relative effectiveness and safety profiles of each treatment, the surface under the cumulative ranking curve (SUCRA) was computed. The SUCRA provides a numerical ranking of each treatment strategy, ranging from 0% to 100%, where higher values indicate a higher probability that the respective strategy is among the best options [16]. Because all included trials compared alternative strategies only against standard DAPT and did not include any direct comparisons between the other five strategies, assessment of inconsistency between direct and indirect estimates was not required. The overall heterogeneity of the various analyses was assessed using the *I*-squared (*I*^2^) statistics. The NMA including SUCRA ranking calculations were performed using R statistical software (version 4.4.2) with the “netmeta” package. Statistical significance was determined at a two-tailed *P* value < 0.05.

Since the clopidogrel de-escalation and reduced-dose P2Y12i categories were each informed by only a single study, they were combined into a single “De-escalation” node, yielding a five-node network. To address potential heterogeneity arising from variations in trial design, we performed additional analyses: restricting inclusion to trials using BARC criteria for bleeding ascertainment; stratifying by timing of therapy switch (< 3 months, 3–5 months, and ≥ 6 months); and categorizing follow-up duration as ≤ 12 months versus > 12 months. We also repeated the analysis after excluding the clopidogrel de-escalation node (TALOS-AMI), and separately restricted the analysis to trials enrolling non–pure ACS populations. Finally, the short-term DAPT followed by P2Y12i monotherapy strategy was sub-divided into potent (ticagrelor-based) and clopidogrel-based approaches, yielding a seven-node network.

## Results

### Search results

**Fig. 1:**
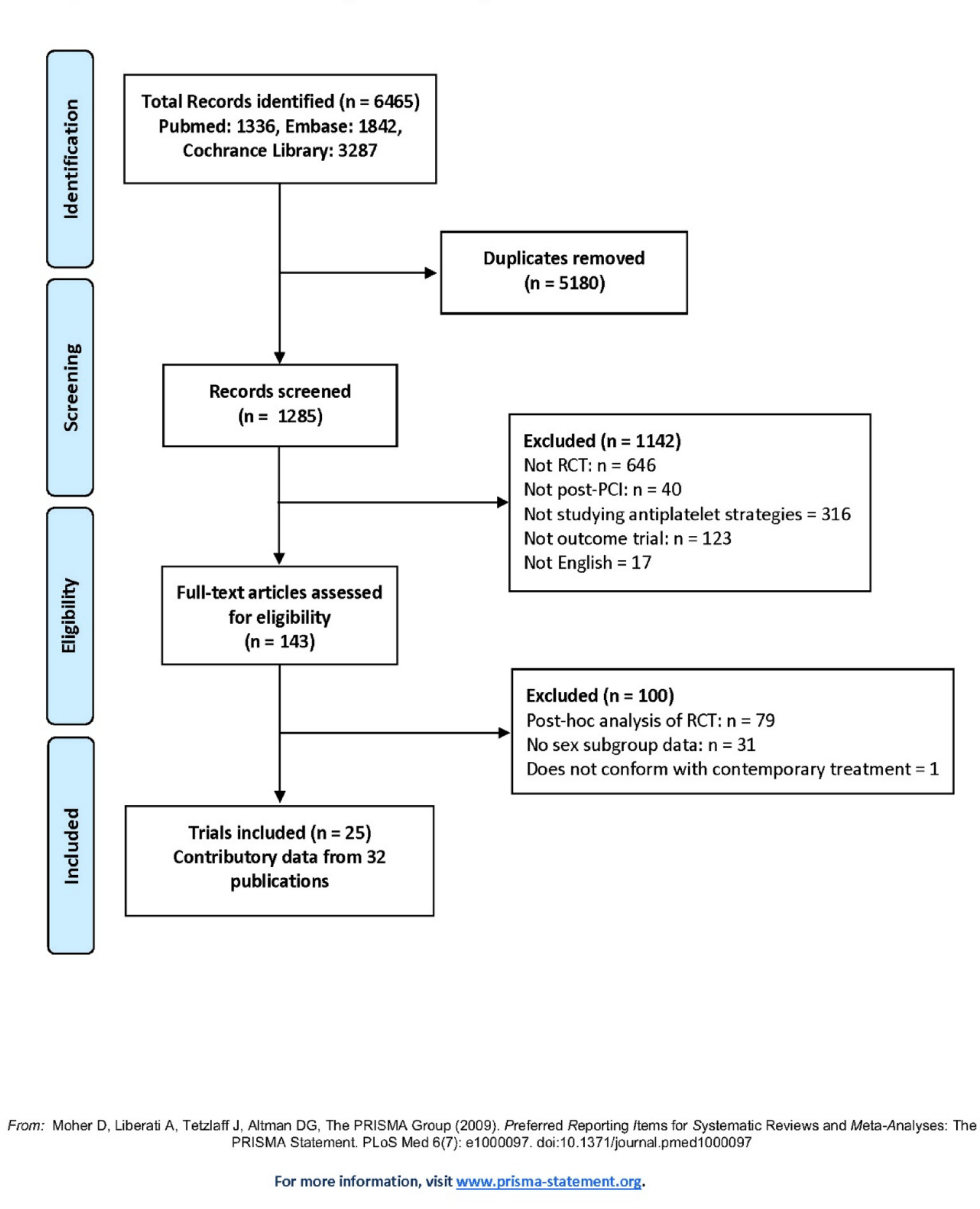
PRISMA flowchart for the identification, inclusion, and exclusion of studies

The initial search identified 1,285 citations. Based on the predefined inclusion criteria, 32 articles (with data from 25 trials) were selected for data extraction, comprising a total of 115,223 male and 38,574 female participants (Fig. 1).

### Risk of bias

The Cochrane Risk of Bias tool assessment indicated a low risk of bias across all 25 included RCTs. A detailed summary of the risk-of-bias evaluations are provided in Supplemental Table 3.

### Study characteristics

The 25 included trials are listed in Table 1. These RCTs compared different antiplatelet therapy strategies following PCI with standard DAPT. Short DAPT was defined as 1–3 months of DAPT followed by monotherapy; guided de-escalation as de-escalation directed by platelet function testing or CYP2C19 genotyping; and reduced-dose P2Y12i as dose reduction of a potent P2Y12i rather than a switch to a less potent agent. The specific agents, durations, and guidance methods for each trial are detailed in Table 1. The distribution of studies across different antiplatelet strategies was as follows: guided de-escalation: 3 studies; short DAPT then aspirin: 10 studies; short DAPT then P2Y12i: 8 studies; short DAPT then either aspirin or P2Y12i: 2 studies; de-escalation with reduced-dose P2Y12i: 1 study; de-escalation to clopidogrel: 1 study. Eleven studies exclusively enrolled patients who underwent PCI for ACS, whereas twelve studies included a mixed population of patients with both chronic coronary syndrome and ACS. Data regarding the total population, event counts, and female-specific numbers and events for each node in the network are summarized in Supplemental Table 4.

### Sex difference in comparative effects of different DAPT strategies on MACE

**Fig. 2:**
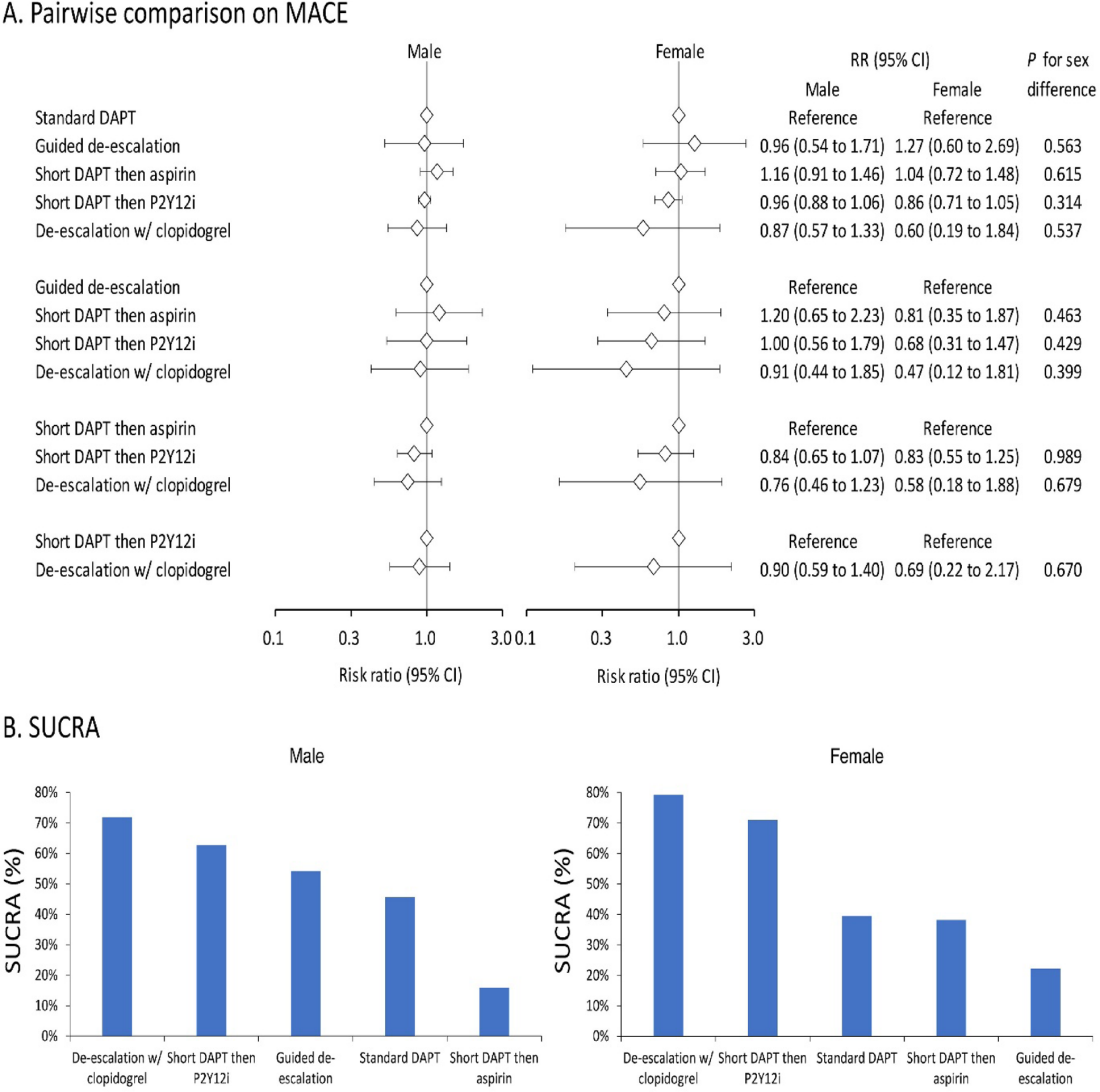
Forest plot (**A**) and SUCRA (**B**) of the network meta-analysis of MACE among patients receiving different dual antiplatelet therapy strategies following percutaneous coronary intervention. Forest plots illustrate the results of all pairwise comparisons, whereas SUCRA provides the simulated comprehensive ranking performance. The SUCRA percentage represents the probability of a treatment being the top-ranked intervention. CI, confidence interval; DAPT, dual antiplatelet therapy; MACE, major adverse cardiovascular events; NACE, net adverse clinical events; P2Y12i, P2Y12 receptor inhibitor; RR, risk ratio; SUCRA, surface under the cumulative ranking curve

For male patients, there was no significant difference in MACE across the various antiplatelet therapy strategies. However, for female patients, a trend was observed in which de-escalation with clopidogrel and short DAPT followed by a P2Y12i appeared to provide a more favorable MACE outcome compared to other strategies, including standard DAPT (Fig. 2A). The SUCRA analysis further demonstrated that these two strategies outperformed all other strategies in both male and female patients, with an even greater relative benefit in females (Fig. 2B). However, no statistically significant differences in treatment effects were observed between male and female patients across the various antiplatelet strategies. In addition, heterogeneity was minimal in both sexes (*I*² = 0% in men and 8.1% in women), as shown in Supplemental Table 5.

### Sex difference in comparative effects of different DAPT strategies on BARC 2, 3, 5 bleeding

**Fig. 3:**
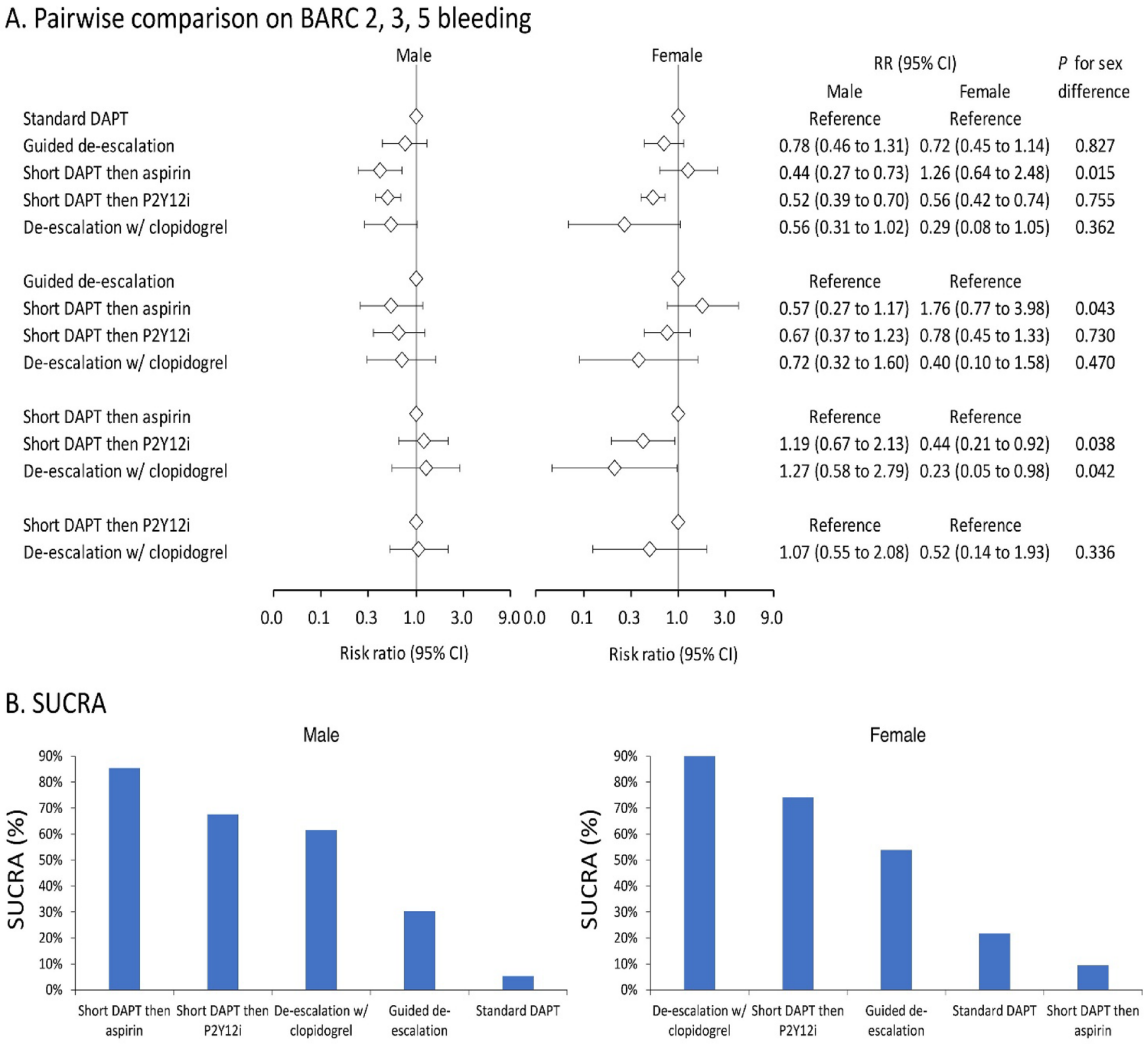
Forest plot (**A**) and SUCRA (**B**) of the network meta-analysis of BARC 2, 3, 5 bleeding event among patients receiving different dual antiplatelet therapy strategies following percutaneous coronary intervention. Forest plots illustrate the results of all pairwise comparisons, whereas SUCRA provides the simulated comprehensive ranking performance. The SUCRA percentage represents the probability of a treatment being the top-ranked intervention. BARC, Bleeding Academic Research Consortium; CI, confidence interval; DAPT, dual antiplatelet therapy; P2Y12i, P2Y12 receptor inhibitor; RR, risk ratio; SUCRA, surface under the cumulative ranking curve

Regardless of whether aspirin or a P2Y12i was continued after short DAPT, both strategies were effective in reducing bleeding risk in male patients compared to standard DAPT strategies, with both reaching statistically significant differences (short DAPT then aspirin: RR 0.44, 95% CI 0.27–0.73; short DAPT then P2Y12i: RR 0.52, 95% CI 0.39–0.70). However, the bleeding reduction benefit of short DAPT followed by aspirin compared to standard DAPT observed in males was not evident in female patients (RR 1.26, 95% CI 0.64–2.28) (Fig. 3A), leading to a significant difference between the two sexes (*P* for sex difference = 0.015). When comparing against short DAPT followed by aspirin as reference, there were no significant differences observed in bleeding risks with short DAPT followed by P2Y12i and de-escalation to clopidogrel in men, yet these two strategies were associated with significantly lower risks of bleeding in women (short DAPT then P2Y12i: RR 0.44, 95% CI 0.21–0.92, *P* for sex difference = 0.038; de-escalation to clopidogrel: RR 0.23, 95% CI 0.05–0.98, *P* for sex difference = 0.042) (Fig. 3A). In terms of SUCRA analysis, in female patients, the strategies that provided the greatest reduction in bleeding risk were de-escalation to clopidogrel and short DAPT followed by a P2Y12i, compared to short DAPT followed by aspirin and short DAPT followed by P2Y12i in men (Fig. 3B). Furthermore, while heterogeneity was negligible among women (*I*² = 0%), it was notably higher among men (*I*² = 45.8%), as detailed in Supplemental Table 5.

### Sex difference in comparative effects of different DAPT strategies on NACE

**Fig. 4:**
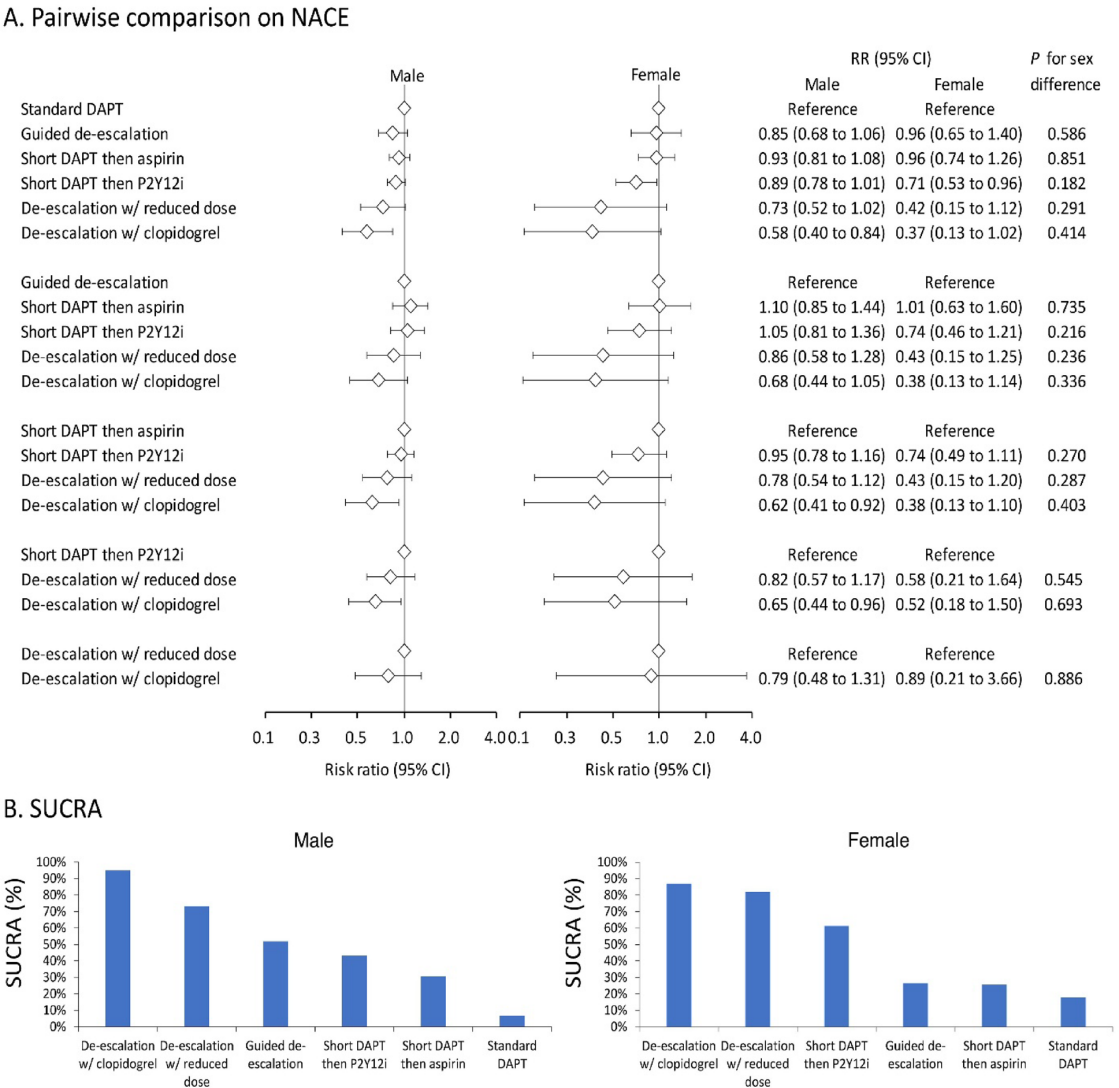
Forest plot (**A**) and SUCRA (**B**) of the network meta-analysis of NACE among patients receiving different dual antiplatelet therapy strategies following percutaneous coronary intervention. Forest plots illustrate the results of all pairwise comparisons, whereas SUCRA provides the simulated comprehensive ranking performance. The SUCRA percentage represents the probability of a treatment being the top-ranked intervention. CI, confidence interval; DAPT, dual antiplatelet therapy; NACE, net adverse clinical events; P2Y12i, P2Y12 receptor inhibitor; RR, risk ratio; SUCRA, surface under the cumulative ranking curve

In both men and women, de-escalation to clopidogrel was associated with the lowest risk of NACE among all DAPT strategies, as evident in forest plot comparisons (Fig. 4A) and SUCRA rankings (Fig. 4B). Other strategies that were associated with lower risks of NACE compared to standard DAPT included de-escalation with reduced-dose P2Y12i and short DAPT followed by P2Y12i. Interestingly, the magnitude of risk reduction, when compared to standard DAPT, seemed numerically greater in women compared to men with de-escalation to clopidogrel (RR 0.37 vs. 0.58), de-escalation with reduced-dose P2Y12i (RR 0.42 vs. 0.73), and short DAPT followed by P2Y12i (RR 0.71 vs. 0.89) (Fig. 4A). However, none of the aforementioned results on sex-based differences reached statistical significance (*P* for interaction > 0.05). Additionally, heterogeneity was trivial among men (*I*² = 8.1%) and modest among women (*I*² = 24.7%), as detailed in Supplemental Table 5.

### Subgroup analysis in patients presenting with ACS

In patients with ACS, regardless of sex, the most favorable strategy for MACE prevention was de-escalation with clopidogrel (Supplemental Figs. 2 A). Similar to the primary analysis cohort, the numerical magnitude of risk reduction was more pronounced in women, albeit without statistically significant differences between the two sexes (Supplemental Fig. 2 A). In terms of bleeding, the short DAPT followed by P2Y12i was associated with the lowest risk in men (RR 0.49, 95% CI 0.34–0.70 compared to standard DAPT), followed by de-escalation to clopidogrel (RR 0.56, 95% CI 0.33–0.95 compared to standard DAPT). In women, however, de-escalation to clopidogrel was associated with the lowest risk (RR 0.29, 95% CI 0.08–1.05 compared to standard DAPT), followed by short DAPT followed by P2Y12i (RR 0.60, 95% CI 0.38–0.93 compared to standard DAPT) (Supplemental Figs. 2B). For NACE outcomes, the trend and rankings were similar in women and men, with de-escalation to clopidogrel associated with the lowest risk in both sexes (Supplemental Figs. 2 C). Similar to the primary analysis cohort, the numerical magnitude of risk reduction associated with de-escalation and short DAPT strategies seemed greater in women compared to men, despite the lack of statistically significant difference between sexes (*P* for interaction > 0.05). Additionally, assessment of heterogeneity was detailed in Supplemental Table 5.

### Sensitivity and additional analyses

To assess the robustness of our primary findings, we conducted several sensitivity and additional analyses. These addressed potential concerns related to node sparsity, heterogeneity in outcome definitions and bleeding ascertainment, timing of therapy switch, type of P2Y12i used, variability in follow-up duration, and differences in study population composition. When the clopidogrel de-escalation and reduced-dose P2Y12i categories were combined into a single “De-escalation” node (five-node network), results were highly consistent with the primary six-node analysis (Supplemental Figs. 3 A–3 C). Similarly, restricting the analysis to studies that used BARC criteria for bleeding ascertainment did not materially alter the findings (Supplemental Fig. 4).

In stratified analyses by timing of therapy switch, a significant sex difference in NACE was observed within the 3–5-month stratum (P for interaction = 0.042), with short DAPT followed by P2Y12i monotherapy associated with a more pronounced protective effect in women than in men (RR 0.40 vs. 0.81; Supplemental Fig. 5 C). Results in the < 3-month and ≥ 6-month strata were broadly consistent with the primary analysis (Supplemental Figs. 6 and 7).

When stratified by follow-up duration, results in the ≤ 12-month subgroup were broadly consistent with the primary analysis (Supplemental Fig. 8A–8 C). In the > 12-month subgroup, however, a significant sex difference emerged for bleeding, with short DAPT followed by aspirin monotherapy demonstrating a more pronounced protective effect in men than in women (RR 0.27 vs. 1.25; P for interaction = 0.001; Supplemental Fig. 9B), while MACE and NACE results remained comparable to the primary analysis (Supplemental Figs. 9 A and 9 C).

Excluding the clopidogrel de-escalation node (TALOS-AMI) did not meaningfully change the results (Supplemental Figs. 10 A–10 C). Likewise, analyses restricted to trials enrolling both CCS and ACS populations yielded findings comparable to those from the primary analysis (Supplemental Figs. 11 A–11 C). Finally, sub-stratifying the short DAPT followed by P2Y12i monotherapy category into potent (ticagrelor-based) and clopidogrel-based strategies produced results consistent with the primary six-node analysis (Supplemental Figs. 12 A–12 C). Heterogeneity statistics across all analyses are reported in Supplemental Table 5.

## Discussion

This network meta-analysis provides a comprehensive evaluation of sex-specific outcomes across various contemporary DAPT strategies following PCI. Our primary findings indicate that for the prevention of MACE, de-escalation strategies are the most favorable for both sexes. In terms of bleeding, different strategies appear optimal for each sex; de-escalation to clopidogrel was associated with the lowest risk in women, whereas a short DAPT duration followed by P2Y12i monotherapy was most favorable for men. For the composite of NACE, which balances ischemic and bleeding risks, de-escalation to clopidogrel emerged as the superior strategy for both men and women.

The search for an optimal balance between antithrombotic efficacy and bleeding risk with DAPT strategies post-PCI remains a central clinical challenge. Previous work, such as a network meta-analysis by Ullah et al., found that while various antiplatelet strategies did not significantly differ for MACE, switching to P2Y12i monotherapy after 1–3 months of DAPT significantly reduced major bleeding [17]. However, that analysis was conducted in a predominantly male population, potentially masking important sex-specific effects. Our findings, which specifically stratify by sex, reveal a more nuanced picture, showing a trend towards either a reduction or a neutral outcome in MACE with de-escalation or short DAPT strategies in both sexes. This challenges the conventional belief that women derive less benefit from antithrombotic therapies. This traditional view is rooted in observations of inherent sex-based differences in platelet physiology and thrombus composition; studies suggest women have smaller coronary vessels and may exhibit higher on-treatment platelet reactivity, while their coronary thrombi may contain less fibrin and P-selectin, which could theoretically blunt the efficacy of standard antiplatelet agents [7]. Despite these physiological distinctions, our results align with emerging evidence suggesting these differences do not translate to poorer outcomes with modern DAPT regimens. Specifically, our finding is consistent with the sex-specific analysis of the MASTER DAPT trial, which showed a favorable trend for reduced ischemic events with an abbreviated DAPT duration in women with high bleeding risk—a benefit not observed in men [14]. This suggests that contemporary antithrombotic strategies provide robust ischemic protection that is at least equivalent in women compared to men, potentially overcoming baseline physiological disadvantages. The need for individualized antiplatelet decision-making is further underscored in the complex PCI setting, where patients face a simultaneously elevated ischemic and bleeding burden; a recent review emphasizes that antithrombotic regimen selection — whether prolonged DAPT, P2Y12i monotherapy, or dual pathway inhibition — must be tailored to each patient’s risk profile and procedural complexity [18]. Our findings suggest that biological sex represents an important additional dimension within this individualization framework, emphasizing the importance of moving beyond a one-size-fits-all model.

The analysis of bleeding outcomes highlights significant and complex sex-specific differences in the benefits derived from various DAPT strategies. Our finding that de-escalation to clopidogrel was most effective for bleeding reduction in women, while short DAPT followed by P2Y12i monotherapy was optimal for men, underscores that a generalized approach to minimizing bleeding risk is inadequate. This is particularly relevant as women are known to have a higher baseline risk of bleeding complications post-PCI, a finding consistently supported by risk models like that from the National Cardiovascular Data Registry [19]. It is also important to note that female participants were often older and carried a higher burden of comorbidities — including diabetes, renal impairment, and anemia — which are themselves independent risk factors for both bleeding and attenuated antiplatelet efficacy. These baseline differences may have contributed to the observed heterogeneity between sexes, and the favorable profile of clopidogrel de-escalation in women may in part reflect its suitability for patients at higher baseline risk, rather than a purely sex-specific biological effect. This elevated risk is often attributed to factors such as lower body weight, higher rates of comorbidities like chronic kidney disease, and potential sex-based variations in drug metabolism. The role of aspirin, a cornerstone of DAPT, introduces further complexity. The paradoxical, albeit non-significant, finding from a secondary analysis of the ADAPTABLE trial—whereby women on 81-mg aspirin had a numerically higher rate of major bleeding than those on 325-mg [20]—is particularly striking and runs counter to conventional dose-response expectations. This counterintuitive signal, combined with our network meta-analysis results favoring clopidogrel de-escalation for women, challenges a uniform approach to DAPT modification. Interestingly, the MASTER DAPT trial found that the overall bleeding benefit from an abbreviated DAPT strategy (followed by P2Y12i monotherapy) was numerically less pronounced in women than in men [14]. This may appear to conflict with our findings but highlights a critical nuance: the type of DAPT modification matters. While P2Y12i monotherapy may be less beneficial for bleeding in women compared to men, our analysis suggests that de-escalating to clopidogrel specifically offers a superior safety profile. This intricate interplay between aspirin dose, P2Y12i choice, and DAPT duration has distinct, sex-specific implications that mandate further investigation in dedicated prospective trials designed to compare these modern strategies head-to-head in female cohorts.

A key finding of this analysis is the consistent superiority of a de-escalation to clopidogrel strategy for improving NACE in both sexes and across different patient populations, including the high-risk ACS subgroup. Although the included trials enrolled heterogeneous populations spanning both ACS and CCS presentations — groups that differ substantially in baseline thrombotic risk and guideline-recommended DAPT duration — sensitivity analyses restricted to non-pure ACS populations yielded results consistent with the primary findings, suggesting that the observed sex-specific treatment effects are not confined to the high-risk ACS setting alone. The magnitude of this net benefit appeared greatest in women, a finding likely attributable to their higher baseline risk for both thrombotic and bleeding events. It is well-documented that women undergoing PCI are often older and have a higher prevalence of comorbidities [21], and female sex itself is an independent predictor of mortality and complications post-ACS [10]. Given this elevated dual risk, a strategy that effectively mitigates both ischemic and bleeding events—as de-escalation to clopidogrel does—logically yields a more pronounced absolute risk reduction and thus a greater net clinical benefit in women. This is strongly supported by the MASTER DAPT trial, which demonstrated a trend toward ischemic benefit with modern DAPT strategies specifically in women [14]. While previous meta-analyses that did not perform sex-specific comparisons favored short DAPT for bleeding reduction [4, 22], our study refines this understanding significantly. We show that the bleeding benefit of short DAPT is primarily driven by male patients. For female patients, de-escalation to clopidogrel was not only superior for NACE but was also the most favorable strategy for reducing bleeding risk. This distinction highlights that de-escalation to clopidogrel provides a uniquely balanced profile of safety and efficacy for women, establishing it as a potentially optimal sex-specific antiplatelet strategy.

A network meta-analysis by Occhipinti et al. [23], published concurrently with our work, similarly examined sex differences in DAPT de-escalation strategies after PCI and reached broadly convergent conclusions: de-escalation by DAPT discontinuation appeared more favorable in women, while P2Y12i switch or dose reduction ranked best in men. Our analysis included 25 trials (153,797 patients) compared to 20 trials (71,272 patients), with the five additional trials — RESET, OPTIMIZE, I-LOVE-IT 2, NIPPON, and HOST-IDEA — contributing predominantly CCS or mixed ACS/CCS populations employing aspirin monotherapy after short DAPT, thereby strengthening the evidentiary base for this treatment node while substantially extending the generalizability of our findings beyond the ACS-enriched setting of the Occhipinti cohort. Notably, while Occhipinti et al. identified aspirin discontinuation as the best overall strategy for women, their 5-node analysis also showed that P2Y12i switch to clopidogrel ranked highest specifically for major bleeding and MACE in women — a finding consistent with our identification of clopidogrel de-escalation as the most favorable strategy for women across bleeding and net clinical outcomes. Together, the two analyses reinforce the hypothesis that sex-specific antiplatelet strategies are warranted after PCI, and that clopidogrel-based approaches may offer the most favorable net clinical profile for women, pending confirmatory prospective evidence.

### Limitations

This study has several limitations inherent to its design. First, because sex effect modification was evaluated using study-level meta-regression rather than individual participant data, patient-level factors that may differ by sex—such as age, body weight, renal function, anemia, and procedural complexity—could not be accounted for. Ecological bias and residual confounding are therefore unavoidable, and the findings should be interpreted as reflecting no detectable interaction at the study level, rather than a definitive absence of sex effect modification. Second, variations in trial design—including the timing of therapy switch, follow-up duration, background gastroprotection practices, concomitant anticoagulation, and stent era—may have introduced heterogeneity and challenged the transitivity assumption, thereby limiting clinical comparability across treatment nodes. Although subgroup analyses were performed to address the first two factors, the lack of granular data on gastroprotection and anticoagulation practices precluded further stratification, and these variables therefore remain potential sources of residual heterogeneity. Third, inconsistencies in endpoint definitions across trials could impact accuracy. We addressed this by prioritizing trials with standardized definitions (e.g., BARC or PLATO bleeding) and ensuring our conclusions were consistent across multiple related outcomes to minimize bias from any single definition. Fourth, the evidence base for several treatment nodes was limited, with single-study nodes potentially compromising the robustness of network estimates—particularly SUCRA-based rankings—and findings from these sparsely populated nodes should therefore be regarded as exploratory. Additionally, female participants were substantially under-represented (38,574 of 153,797 [25.1%]), potentially limiting statistical power to detect sex-based differences in treatment effects across strategies. Despite these limitations, this study employed statistical methods to minimize potential bias and provides valuable data to promote further reference and discussion in the field.

## Conclusion

This study demonstrates sex-based differences in the optimal DAPT strategy following PCI. In men, short DAPT followed by aspirin or P2Y12i monotherapy robustly reduced bleeding risk across multiple treatment nodes and sensitivity analyses. In women, de-escalation to clopidogrel showed the most favorable overall profile, with statistically significant sex differences in bleeding outcomes, but should be considered hypothesis-generating, requiring confirmatory evidence from head-to-head trials or individual patient data meta-analyses before broad clinical adoption. These findings are most applicable to women without high thrombotic risk, and underscore the need for dedicated prospective trials evaluating sex-specific antiplatelet strategies after PCI.

## Supplementary Information

Supplementary Material 1

Supplementary Material 2

Supplementary Material 3

Supplementary Material 4

Supplementary Material 5

Supplementary Material 6: Fig. S1. Network plots for the primary 6-node analysis on MACE (A), bleeding (B), and NACE (C). Node sizes and edge weights are scaled to the total sample size and the strength (number of studies) of direct head-to-head comparisons, respectively. DAPT, dual antiplatelet therapy; MACE, major adverse cardiovascular events; NACE, net adverse clinical events; P2Y12i, P2Y12 receptor inhibitor.

Supplementary Material 7

Supplementary Material 8

Supplementary Material 9: Fig. S2. Forest plot and SUCRA of the network meta-analysis of MACE (A), BARC 2, 3, 5 bleeding event (B) and NACE (C) among patients receiving different dual antiplatelet therapy strategies following percutaneous coronary intervention for pure acute coronary syndrome population. BARC, Bleeding Academic Research Consortium; CI, confidence interval; DAPT, dual antiplatelet therapy; MACE, major adverse cardiovascular events; NACE, net adverse clinical events; P2Y12i, P2Y12 receptor inhibitor; RR, risk ratio; SUCRA, surface under the cumulative ranking curve.

Supplementary Material 10

Supplementary Material 11

Supplementary Material 12: Fig. S3. Forest plot and SUCRA of the network meta-analysis of MACE (A), BARC 2, 3, 5 bleeding (B), and NACE (C) among patients receiving different dual antiplatelet therapy strategies following percutaneous coronary intervention, using a five-node network in which the clopidogrel de-escalation and reduced-dose P2Y12 inhibitor categories were combined into a single “De-escalation” node. Supplemental BARC, Bleeding Academic Research Consortium; CI, confidence interval; DAPT, dual antiplatelet therapy; MACE, major adverse cardiovascular events; NACE, net adverse clinical events; P2Y12i, P2Y12 receptor inhibitor; RR, risk ratio; SUCRA, surface under the cumulative ranking curve.

Supplementary Material 13

Supplementary Material 14

Supplementary Material 15: Fig. S4. Forest plot and SUCRA of the network meta-analysis of BARC 2, 3, 5 bleeding among patients receiving different dual antiplatelet therapy strategies following percutaneous coronary intervention, restricted to trials using Bleeding Academic Research Consortium (BARC) criteria for bleeding ascertainment. BARC, Bleeding Academic Research Consortium; CI, confidence interval; DAPT, dual antiplatelet therapy; MACE, major adverse cardiovascular events; NACE, net adverse clinical events; P2Y12i, P2Y12 receptor inhibitor; RR, risk ratio; SUCRA, surface under the cumulative ranking curve.

Supplementary Material 16: Fig. S5. Forest plot and SUCRA of the network meta-analysis of MACE (A), BARC 2, 3, 5 bleeding (B), and NACE (C) among patients receiving different dual antiplatelet therapy strategies following percutaneous coronary intervention, restricted to trials with a therapy switch timing of 3–5 months. BARC, Bleeding Academic Research Consortium; CI, confidence interval; DAPT, dual antiplatelet therapy; MACE, major adverse cardiovascular events; NACE, net adverse clinical events; P2Y12i, P2Y12 receptor inhibitor; RR, risk ratio; SUCRA, surface under the cumulative ranking curve.

Supplementary Material 17

Supplementary Material 18

Supplementary Material 19: Fig. S6. Forest plot and SUCRA of the network meta-analysis of MACE (A), BARC 2, 3, 5 bleeding (B), and NACE (C) among patients receiving different dual antiplatelet therapy strategies following percutaneous coronary intervention, restricted to trials with a therapy switch timing of less than 3 months. BARC, Bleeding Academic Research Consortium; CI, confidence interval; DAPT, dual antiplatelet therapy; MACE, major adverse cardiovascular events; NACE, net adverse clinical events; P2Y12i, P2Y12 receptor inhibitor; RR, risk ratio; SUCRA, surface under the cumulative ranking curve.

Supplementary Material 20

Supplementary Material 21

Supplementary Material 22: Fig. S7. Forest plot and SUCRA of the network meta-analysis of MACE (A), BARC 2, 3, 5 bleeding (B), and NACE (C) among patients receiving different dual antiplatelet therapy strategies following percutaneous coronary intervention, restricted to trials with a therapy switch timing of 6 months or greater. BARC, Bleeding Academic Research Consortium; CI, confidence interval; DAPT, dual antiplatelet therapy; MACE, major adverse cardiovascular events; NACE, net adverse clinical events; P2Y12i, P2Y12 receptor inhibitor; RR, risk ratio; SUCRA, surface under the cumulative ranking curve.

Supplementary Material 23

Supplementary Material 24

Supplementary Material 25: Fig. S8. Forest plot and SUCRA of the network meta-analysis of MACE (A), BARC 2, 3, 5 bleeding (B), and NACE (C) among patients receiving different dual antiplatelet therapy strategies following percutaneous coronary intervention, restricted to trials with a follow-up duration of 12 months or less. BARC, Bleeding Academic Research Consortium; CI, confidence interval; DAPT, dual antiplatelet therapy; MACE, major adverse cardiovascular events; NACE, net adverse clinical events; P2Y12i, P2Y12 receptor inhibitor; RR, risk ratio; SUCRA, surface under the cumulative ranking curve.

Supplementary Material 26

Supplementary Material 27

Supplementary Material 28: Fig. S9. Forest plot and SUCRA of the network meta-analysis of MACE (A), BARC 2, 3, 5 bleeding (B), and NACE (C) among patients receiving different dual antiplatelet therapy strategies following percutaneous coronary intervention, restricted to trials with a follow-up duration of more than 12 months. BARC, Bleeding Academic Research Consortium; CI, confidence interval; DAPT, dual antiplatelet therapy; MACE, major adverse cardiovascular events; NACE, net adverse clinical events; P2Y12i, P2Y12 receptor inhibitor; RR, risk ratio; SUCRA, surface under the cumulative ranking curve.

Supplementary Material 29

Supplementary Material 30

Supplementary Material 31: Fig. S10. Forest plot and SUCRA of the network meta-analysis of MACE (A), BARC 2, 3, 5 bleeding (B), and NACE (C) among patients receiving different dual antiplatelet therapy strategies following percutaneous coronary intervention, after exclusion of the clopidogrel de-escalation node (TALOS-AMI), yielding a five-node network. BARC, Bleeding Academic Research Consortium; CI, confidence interval; DAPT, dual antiplatelet therapy; MACE, major adverse cardiovascular events; NACE, net adverse clinical events; P2Y12i, P2Y12 receptor inhibitor; RR, risk ratio; SUCRA, surface under the cumulative ranking curve.

Supplementary Material 32

Supplementary Material 33

Supplementary Material 34: Fig. S11. Forest plot and SUCRA of the network meta-analysis of MACE (A), BARC 2, 3, 5 bleeding (B), and NACE (C) among patients receiving different dual antiplatelet therapy strategies following percutaneous coronary intervention, restricted to trials enrolling both chronic and acute coronary syndrome populations. BARC, Bleeding Academic Research Consortium; CI, confidence interval; DAPT, dual antiplatelet therapy; MACE, major adverse cardiovascular events; NACE, net adverse clinical events; P2Y12i, P2Y12 receptor inhibitor; RR, risk ratio; SUCRA, surface under the cumulative ranking curve.

Supplementary Material 35

Supplementary Material 36

Supplementary Material 37: Fig. S12. Forest plot and SUCRA of the network meta-analysis of MACE (A), BARC 2, 3, 5 bleeding (B), and NACE (C) among patients receiving different dual antiplatelet therapy strategies following percutaneous coronary intervention, using a seven-node network in which the short DAPT followed by P2Y12 inhibitor monotherapy category was sub-divided into potent (ticagrelor-based) and clopidogrel-based strategies. BARC, Bleeding Academic Research Consortium; CI, confidence interval; DAPT, dual antiplatelet therapy; MACE, major adverse cardiovascular events; NACE, net adverse clinical events; P2Y12i, P2Y12 receptor inhibitor; RR, risk ratio; SUCRA, surface under the cumulative ranking curve.

Supplementary Material 38

Supplementary Material 39

## Data Availability

The datasets used and/or analyzed during the current study are available from the corresponding author on reasonable request.
