# Supplementary material for: Sex-specific efficacy and safety of short-term and de-escalation DAPT strategies after PCI: a network meta-analysis
Source: Biol Sex Differ. 2026 Apr 22;17:114. doi: 10.1186/s13293-026-00903-y (PMC13235097; doi:10.1186/s13293-026-00903-y)
Supplement: Supplementary file 1 — Supplementary Material 1 [file 13293_2026_903_MOESM1_ESM.docx]

**Supplemental table 1. Details of search strategies**

| **Pubmed Search:**  ((((((((((((((antiplatelet therapy[Title]) OR (de-escalation[Title])) OR (switch*[Title])) OR (platelet function monitoring[Title])) OR (genotype-guided[Title])) OR (P2Y12 monotherapy[Title])) OR (ticagrelor monotherapy[Title])) OR (prasugrel monotherapy[Title])) OR (aspirin monotherapy[Title])) OR (ticagrelor[Title])) OR (prasugrel[Title])) OR (clopidogrel[Title])) OR (aspirin[Title])) OR (acetylsalicylic acid[Title])) AND (((coronary intervention[Title/Abstract]) OR (angioplasty[Title/Abstract])) OR (stent[Title/Abstract])) AND (random*[Title/Abstract]) AND ("2009/01/01"[Date - Publication] : "2024"[Date - Publication]) |
| --- |
| **Embase Search:**  ('antiplatelet therapy':ti OR 'de escalation':ti OR switch*:ti OR 'platelet function monitoring':ti OR 'genotype guided':ti OR 'p2y12 monotherapy':ti OR 'ticagrelor monotherapy':ti OR 'prasugrel monotherapy':ti OR 'aspirin monotherapy':ti OR ticagrelor:ti OR prasugrel:ti OR clopidogrel:ti OR aspirin:ti OR 'acetylsalicylic acid':ti) AND ('coronary intervention':ab,ti OR angioplasty:ab,ti OR stent:ab,ti) AND 'randomized controlled trial' AND [2009-2024]/py |
| **Cochrane Library Search:**  (( "antiplatelet therapy" OR de-escalation OR switch* OR "platelet function monitoring" OR genotype-guided OR "P2Y12 monotherapy" OR "ticagrelor monotherapy" OR "prasugrel monotherapy" OR "aspirin monotherapy" OR ticagrelor OR prasugrel OR clopidogrel OR aspirin OR "acetylsalicylic acid" )):ti,ab,kw AND (( "coronary intervention" OR angioplasty OR stent )):ti,ab,kw AND (random*):ti,ab,kw |
