# Supplementary material for: Sex-specific efficacy and safety of short-term and de-escalation DAPT strategies after PCI: a network meta-analysis
Source: Biol Sex Differ. 2026 Apr 22;17:114. doi: 10.1186/s13293-026-00903-y (PMC13235097; doi:10.1186/s13293-026-00903-y)
Supplement: Supplementary file 2 — Supplementary Material 2 [file 13293_2026_903_MOESM2_ESM.docx]

**Supplemental table 2. Outcome definitions of included trials**

**MACE**

| Trial name | Outcome definition |
| --- | --- |
| PRODIGY | Death of any cause, nonfatal myocardial infarction, or cerebrovascular accident |
| RESET | Death from any cause, myocardial infarction, or stent thrombosis |
| OPTIMIZE | Death from any cause, MI, emergent coronary artery bypass graft surgery, or target-  lesion revascularization |
| ISAR-SAFE | Composite of death, myocardial infarction, definite or probable stent thrombosis or stroke |
| I-LOVE-IT 2 | Cardiac death, target vessel myocardial infarction MI, or clinically indicated target lesion revascularization |
| IVUS-XPL | Cardiac death, MI, or repeat revascularization of stented lesions |
| ANTARCTIC | Cardiovascular death, myocardial infarction, definite stent thrombosis, or urgent revascularization |
| NIPPON | All-cause death, MI, and cerebrovascular events |
| TROPICAL-ACS | Death from cardiovascular causes, myocardial infarction, or stroke |
| SMART-DATE | All-cause mortality, myocardial infarction, or stroke |
| REDUCE | All-cause death, myocardial infarction, stent thrombosis, stroke, target vessel revascularization |
| GLOBAL LEADERS | All-cause mortality, any stroke, any myocardial infarction or any revascularization |
| SMART-CHOICE | All-cause death, myocardial infarction, or stroke |
| POPular Genetics | Death from vascular causes, myocardial infarction, definite stent thrombosis, or stroke |
| TWILIGHT | Death from any cause, nonfatal myocardial infarction, or nonfatal stroke |
| TICO | All-cause death, myocardial infarction, stent thrombosis, stroke, or target-vessel revascularization |
| HOST-REDUCE-POLYTECH-ACS | Cardiovascular death, myocardial infarction, stent thrombosis, and ischemic stroke |
| One-Month DAPT | Not provided |
| TALOS-AMI | Cardiovascular death, MI, stroke, any revascularization, or stent thrombosis |
| STOPDAPT-2 ACS | Cardiovascular death, myocardial infarction, definite stent thrombosis, or any stroke |
| HOST- IDEA | Cardiac death, target vessel MI, or clinically driven target lesion revascularization |
| T-PASS | Cardiovascular death, myocardial infarction, stent thrombosis, or ischemia-driven target-vessel revascularization |
| MASTER DAPT | Death due to any cause, myocardial infarction, or stroke |
| SHARE | Cardiac death, myocardial infarction, stent thrombosis, stroke, or ischemia-driven target lesion revascularization |
| ULTIMATE-DAPT | Cardiac death, myocardial infarction, ischemic stroke, definite stent thrombosis,  or clinically driven target vessel revascularization |

**Bleeding**

| Trial name | Outcome definition |
| --- | --- |
| PRODIGY | BARC 2, 3, or 5 bleeding |
| RESET | TIMI major or minor bleeding |
| OPTIMIZE | Any bleeding (major bleeding plus bleeding events that did not meet criteria for either major or severe or life-threatening bleeding according to modified major REPLACE-2 and severe or life-threatening GUSTO criteria) |
| ISAR-SAFE | TIMI major or minor bleeding |
| I-LOVE-IT 2 | Major bleeding (Bleeding Academic Research Consortium type ≥3 bleeding) |
| IVUS-XPL | TIMI major bleeding |
| ANTARCTIC | BARC 2, 3, or 5 bleeding |
| NIPPON | BARC 3 or 5 bleeding |
| TROPICAL-ACS | BARC 2, 3, or 5 bleeding |
| SMART-DATE | BARC 2, 3, or 5 bleeding |
| REDUCE | BARC 2, 3, or 5 bleeding |
| GLOBAL LEADERS | BARC 3 or 5 bleeding |
| SMART-CHOICE | BARC 2, 3, or 5 bleeding |
| POPular Genetics | PLATO major bleeding |
| TWILIGHT | BARC 2, 3, or 5 bleeding |
| TICO | TIMI major bleeding |
| HOST-REDUCE-POLYTECH-ACS | BARC bleeding types 2, 3, or 5 |
| One-Month DAPT | STEEPLE trial criteria major bleeding |
| TALOS-AMI | BARC 2, 3, or 5 bleeding |
| STOPDAPT-2 ACS | TIMI major or minor bleeding |
| HOST- IDEA | BARC 3 or 5 bleeding |
| T-PASS | BARC 3 or 5 bleeding |
| MASTER DAPT | BARC 2, 3, or 5 bleeding |
| SHARE | BARC 3 or 5 bleeding |
| ULTIMATE-DAPT | BARC 2, 3, or 5 bleeding |

**NACE**

| Trial name | Outcome definition |
| --- | --- |
| PRODIGY | Death of any cause, nonfatal myocardial infarction, cerebrovascular accident, or BARC bleeding types 2, 3, or 5 |
| RESET | Death from cardiovascular cause, myocardial infarction, stent thrombosis, ischemia-driven target-vessel revascularization, or TIMI major or minor bleeding |
| OPTIMIZE | All-cause death, myocardial infarction, stroke, or major bleeding |
| ISAR-SAFE | All-cause death, myocardial infarction, stent thrombosis (definite or probable), stroke, or TIMI major bleeding |
| I-LOVE-IT 2 | All-cause death, all MI, stroke, and major bleeding (Bleeding Academic Research Consortium type ≥3 bleeding) |
| IVUS-XPL | Cardiac death, myocardial infarction, stroke, or TIMI major bleeding |
| ANTARCTIC | Cardiovascular death, myocardial infarction, stroke, definite stent thrombosis (Academic Research Consortium definition), urgent revascularization, or bleeding (BARC definition types 2, 3, or 5) |
| NIPPON | All cause death, Q-wave or non–Q-wave MI, cerebrovascular events, or major bleeding events |
| TROPICAL-ACS | Death from cardiovascular causes, myocardial infarction, stroke, or BARC 2, 3, or 5 bleeding |
| SMART-DATE | All-cause mortality, myocardial infarction, stroke, or BARC 2, 3, or 5 bleeding |
| REDUCE | All-cause death, myocardial infarction, stent thrombosis, stroke, target vessel revascularization, or BARC 2, 3, or 5 bleeding |
| GLOBAL LEADERS | All-cause mortality, any stroke, any myocardial infarction or any revascularization, or BARC 3 or 5 bleeding |
| SMART-CHOICE | All-cause death, myocardial infarction, stroke, or BARC 2, 3, or 5 bleeding |
| POPular Genetics | Death from any cause, myocardial infarction, definite stent thrombosis, stroke, or PLATO major bleeding |
| TWILIGHT | Not provided |
| TICO | All-cause death, myocardial infarction, stent thrombosis, stroke, target-vessel revascularization, or TIMI major bleeding |
| HOST-REDUCE-POLYTECH-ACS | Cardiovascular death, myocardial infarction, stent thrombosis, and ischemic stroke, or BARC bleeding types 2, 3, or 5 |
| One-Month DAPT | Cardiac death, nonfatal myocardial infarction, target vessel revascularization, stroke, or major bleeding |
| TALOS-AMI | Cardiovascular death, MI, stroke, any revascularization, or stent thrombosis, or BARC bleeding types 2, 3, or 5 |
| STOPDAPT-2 ACS | Cardiovascular death, myocardial infarction, definite stent thrombosis, any stroke,  TIMI major or minor bleeding |
| HOST- IDEA | Cardiac death, target vessel myocardial infarction, clinically driven target lesion revascularization, stent thrombosis, or BARC type 3 or 5 bleeding |
| T-PASS | All-cause death, myocardial infarction, stent thrombosis, stroke, or major bleeding |
| MASTER DAPT | Death due to any cause, myocardial infarction, stroke, or major bleeding |
| SHARE | Cardiac death, myocardial infarction, stent thrombosis, stroke, ischemia-driven target lesion revascularization, or BARC 3 or 5 bleeding |
| ULTIMATE-DAPT | Cardiac death, myocardial infarction, ischemic stroke, definite stent thrombosis,  clinically driven target vessel revascularization, or any BARC bleeding [types 1, 2, 3, or 5] |
