# Supplementary material for: Sex-specific efficacy and safety of short-term and de-escalation DAPT strategies after PCI: a network meta-analysis
Source: Biol Sex Differ. 2026 Apr 22;17:114. doi: 10.1186/s13293-026-00903-y (PMC13235097; doi:10.1186/s13293-026-00903-y)
Supplement: Supplementary file 3 — Supplementary Material 3 [file 13293_2026_903_MOESM3_ESM.docx]

**Supplemental table 3. Risk of bias summary**

|  | **Random sequence generation (selection bias)** | **Allocation concealment  (selection bias)** | **Blinding of participants and personnel (performance bias)** | **Blinding of outcome assessment (detection bias)  (patient-reported outcomes)** | **Blinding of outcome assessment (detection bias) (Mortality)** | **Incomplete outcome data addressed (attrition bias) (Short-term outcomes (2-6 weeks))** | **Incomplete outcome data addressed (attrition bias) (Longer-term outcomes (>6 weeks))** | **Selective reporting (reporting bias)** |
| --- | --- | --- | --- | --- | --- | --- | --- | --- |
| **PRODIGY** | Low | Low | Unclear | Low | Low | Low | Low | Low |
| **RESET** | Low | Low | Unclear | Low | Low | Low | Low | Low |
| **OPTIMIZE** | Low | Low | Unclear | Low | Low | Low | Low | Low |
| **ISAR-SAFE** | Low | Low | Low | Low | Low | Low | Low | Low |
| **I-LOVE-IT 2** | Low | Low | Unclear | Low | Low | Low | Low | Low |
| **IVUS-XPL** | Low | Low | Unclear | Low | Low | Low | Low | Low |
| **ANTARCTIC** | Low | Low | Unclear | Low | Low | Low | Low | Low |
| **NIPPON** | Low | Low | Unclear | Low | Low | Low | Low | Low |
| **TROPICAL-ACS** | Low | Low | Unclear | Low | Low | Low | Low | Low |
| **SMART-DATE** | Low | Low | Unclear | Low | Low | Low | Low | Low |
| **REDUCE** | Low | Low | Unclear | Low | Low | Low | Low | Low |
| **GLOBAL LEADERS** | Low | Low | Unclear | Low | Low | Low | Low | Low |
| **SMART-CHOICE** | Low | Low | Unclear | Low | Low | Low | Low | Low |
| **POPular Genetics** | Low | Low | Unclear | Low | Low | Low | Low | Low |
| **TWILIGHT** | Low | Low | Low | Low | Low | Low | Low | Low |
| **TICO** | Low | Low | Unclear | Low | Low | Low | Low | Low |
| **HOST-REDUCE-POLYTECH-ACS** | Low | Low | Unclear | Low | Low | Low | Low | Low |
| **One-Month DAPT** | Low | Low | Unclear | Low | Low | Low | Low | Low |
| **TALOS-AMI** | Low | Low | Unclear | Low | Low | Low | Low | Low |
| **STOPDAPT-2 ACS** | Low | Low | Unclear | Low | Low | Low | Low | Low |
| **HOST- IDEA** | Low | Low | Unclear | Low | Low | Low | Low | Low |
| **T-PASS** | Low | Low | Unclear | Low | Low | Low | Low | Low |
| **MASTER DAPT** | Low | Low | Unclear | Low | Low | Low | Low | Low |
| **SHARE** | Low | Low | Unclear | Low | Low | Low | Low | Low |
| **ULTIMATE-DAPT** | Low | Low | Low | Low | Low | Low | Low | Low |
