# Supplementary material for: Sex-specific efficacy and safety of short-term and de-escalation DAPT strategies after PCI: a network meta-analysis
Source: Biol Sex Differ. 2026 Apr 22;17:114. doi: 10.1186/s13293-026-00903-y (PMC13235097; doi:10.1186/s13293-026-00903-y)
Supplement: Supplementary file 4 — Supplementary Material 4 [file 13293_2026_903_MOESM4_ESM.docx]

**Supplemental Table 4**. Summary of sample size and events by treatment node, including total participants, total events, number of women, and events among women

| Outcome | Standard DAPT | Guided  de-escalation | Short DAPT then aspirin | Short DAPT then P2Y12i | De-escalation w/ reduced dose | De-escalation w/ clopidogrel |
| --- | --- | --- | --- | --- | --- | --- |
| MACE |  |  |  |  |  |  |
| Study no. | 12 | 1 | 3 | 7 | 0 | 1 |
| No. of pts. | 24082 | 435 | 3249 | 18989 | 0 | 1349 |
| No. of events | 1679* | 43 | 131 | 1387 | 0 | 47 |
| No. of females | 6025 | 164 | 875 | 4826 | 0 | 217 |
| Events of females | 427 | 17 | 43 | 322 | 0 | 5 |
| BARC 235 bleeding |  |  |  |  |  |  |
| Study no. | 9 | 1 | 2 | 5 | 0 | 1 |
| No. of pts. | 14694 | 1242 | 1734 | 10335 | 0 | 1349 |
| No. of events | 813 | 122 | 58 | 268 | 0 | 38 |
| No. of females | 3354 | 317 | 367 | 2440 | 0 | 217 |
| Events of females | 216 | 37 | 20 | 82 | 0 | 3 |
| NACE |  |  |  |  |  |  |
| Study number | 19 | 3# | 9 | 5 | 1 | 1 |
| No. of pts. | 29014 | 1677 | 11104 | 13665 | 1170 | 1349 |
| No. of events | 2495 | 183 | 573 | 1318 | 82 | 59 |
| No. of females | 7234 | 481 | 3043 | 3369 | 120 | 217 |
| Events of females | 656 | 69 | 166 | 318 | 7 | 6 |

* Event counts were not available for PRODIGY.

# The numbers of participants and events were not reported in TROPICAL-ACS.
