# Supplementary material for: Sex-specific efficacy and safety of short-term and de-escalation DAPT strategies after PCI: a network meta-analysis
Source: Biol Sex Differ. 2026 Apr 22;17:114. doi: 10.1186/s13293-026-00903-y (PMC13235097; doi:10.1186/s13293-026-00903-y)
Supplement: Supplementary file 5 — Supplementary Material 5 [file 13293_2026_903_MOESM5_ESM.docx]

**Supplemental Table 5**. The heterogeneity of the various analyses according to the *I*-squared statistics

| Outcome  Analysis | MACE | | BARC 2, 3, 5 bleeding | | NACE | |
| --- | --- | --- | --- | --- | --- | --- |
|  | Male | Female | Male | Female | Male | Female |
| Different number of nodes |  |  |  |  |  |  |
| 6 nodes (primary analysis) | 0 | 8.1 | 45.8 | 0 | 8.1 | 24.7 |
| 5 nodes (clopidogrel de-escalation and reduced-dose P2Y12i were combined) | 0 | 8.1 | 45.8 | 0 | 7.2 | 19.5 |
| 7 nodes (reduced-dose P2Y12i were divided into clopidogrel and ticagrelor) | 0 | 0 | 60.3 | 0 | 9.8 | 15.3 |
| Follow duration |  |  |  |  |  |  |
| ≤12 months | 0 | 19.7 | 0 | 0 | 0 | 9.1 |
| >12 months | 0 | 0 | NA | NA | 76.6 | 23.2 |
| Timing of therapy switch |  |  |  |  |  |  |
| <3 months | 45.7 | 0 | 0 | 0 | 61.9 | 44.8 |
| 3-5 months | 0 | 31.1 | 0 | 0 | 0 | 0 |
| ≥6 months | 0 | 19.4 | NA | NA | 33 | 18.1 |
| Population |  |  |  |  |  |  |
| Pure ACS | 40.7 | 42.5 | 24.9 | 0 | 24.2 | 27.5 |
| Non-pure ACS | 0 | 0 | 0 | 0 | 6.8 | 23.1 |
| BARC bleeding criteria | - | - | 59.4 | 0 | - | - |
| Remove TALOS trial | 0 | 8.1 | 45.8 | 0 | 8.1 | 24.7 |
