# Supplementary figures and images for: Sex-specific efficacy and safety of short-term and de-escalation DAPT strategies after PCI: a network meta-analysis
Source: Biol Sex Differ. 2026 Apr 22;17:114. doi: 10.1186/s13293-026-00903-y (PMC13235097; doi:10.1186/s13293-026-00903-y)

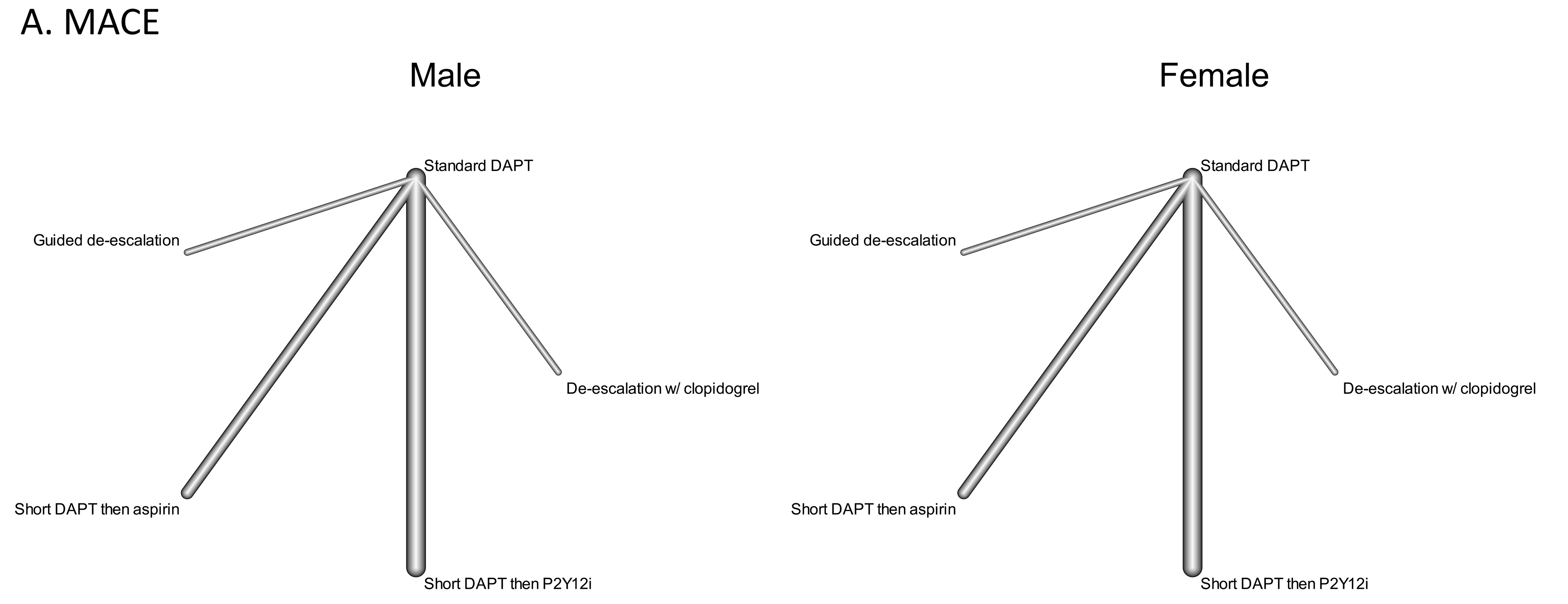

Supplement: Supplementary file 6 — Supplementary Material 6: Fig. S1. Network plots for the primary 6-node analysis on MACE (A), bleeding (B), and NACE (C). Node sizes and edge weights are scaled to the total sample size and the strength (number of studies) of direct head-to-head comparisons, respectively. DAPT, dual antiplatelet therapy; MACE, major adverse cardiovascular events; NACE, net adverse clinical events; P2Y12i, P2Y12 receptor inhibitor. [file 13293_2026_903_MOESM6_ESM.tif]

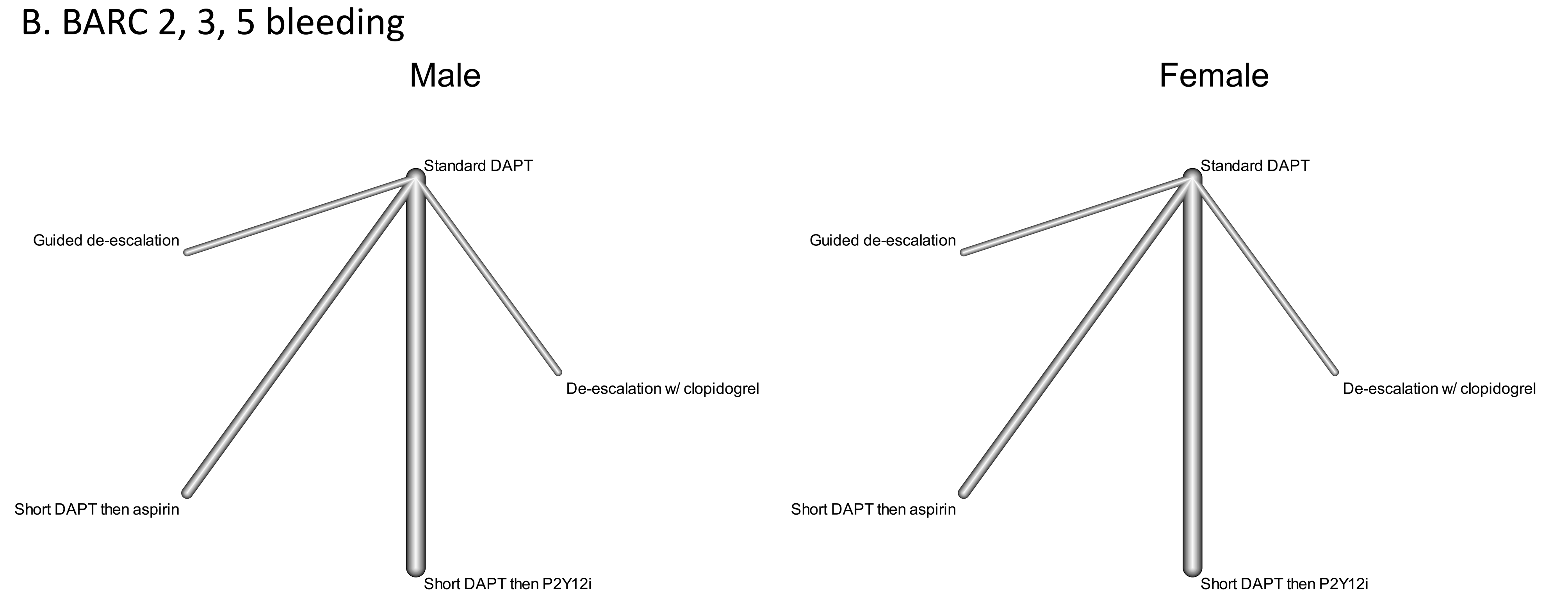

Supplement: Supplementary file 7 — Supplementary Material 7 [file 13293_2026_903_MOESM7_ESM.tif]

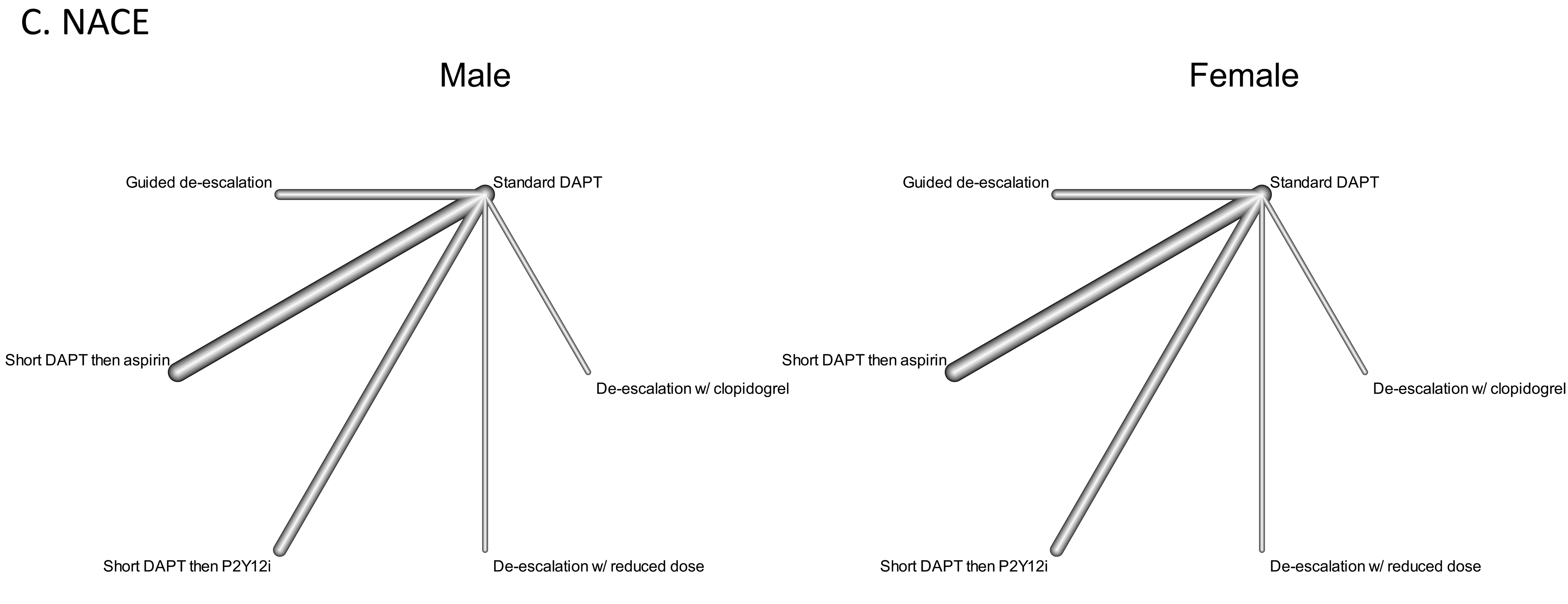

Supplement: Supplementary file 8 — Supplementary Material 8 [file 13293_2026_903_MOESM8_ESM.tif]

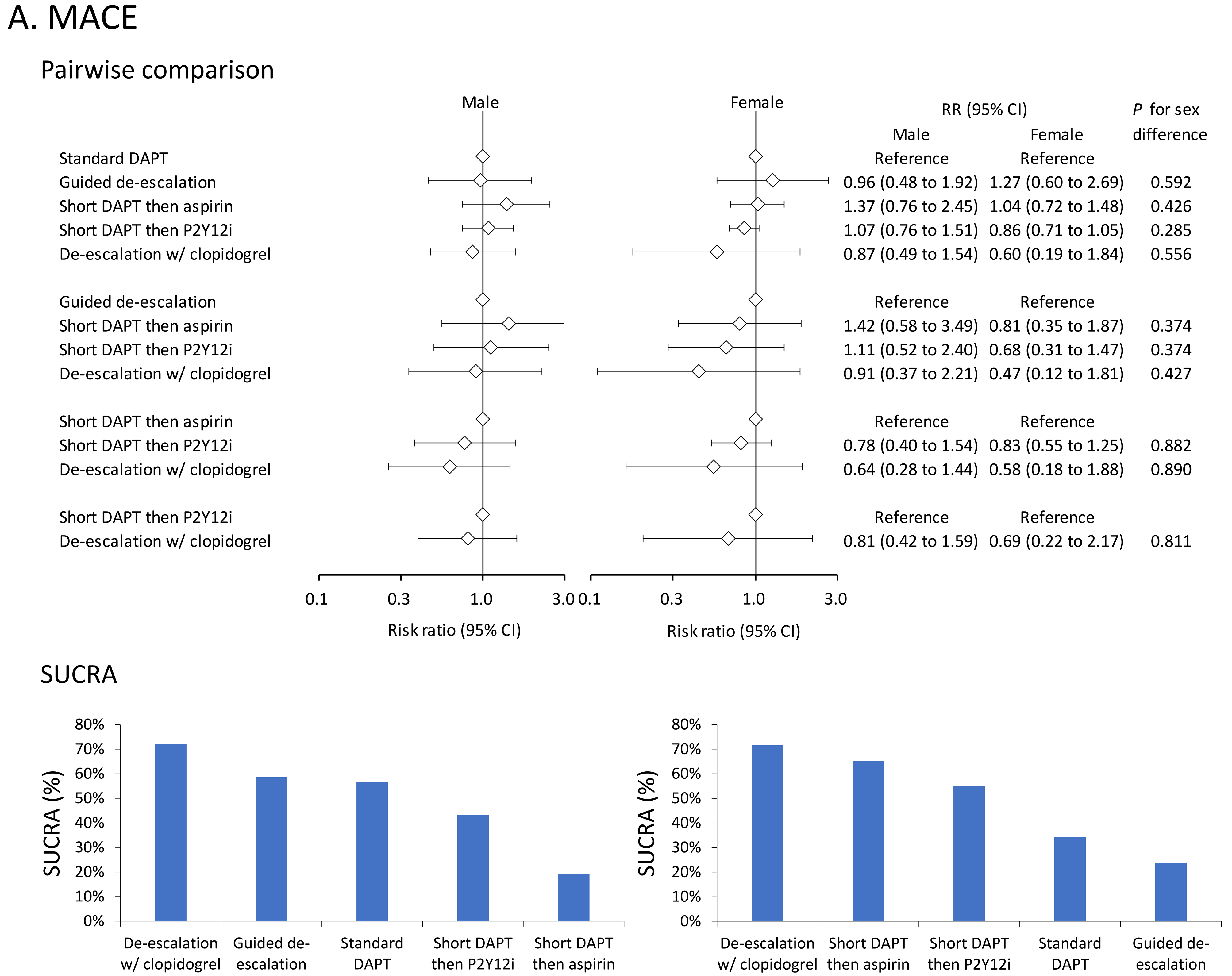

Supplement: Supplementary file 9 — Supplementary Material 9: Fig. S2. Forest plot and SUCRA of the network meta-analysis of MACE (A), BARC 2, 3, 5 bleeding event (B) and NACE (C) among patients receiving different dual antiplatelet therapy strategies following percutaneous coronary intervention for pure acute coronary syndrome population. BARC, Bleeding Academic Research Consortium; CI, confidence interval; DAPT, dual antiplatelet therapy; MACE, major adverse cardiovascular events; NACE, net adverse clinical events; P2Y12i, P2Y12 receptor inhibitor; RR, risk ratio; SUCRA, surface under the cumulative ranking curve. [file 13293_2026_903_MOESM9_ESM.tif]

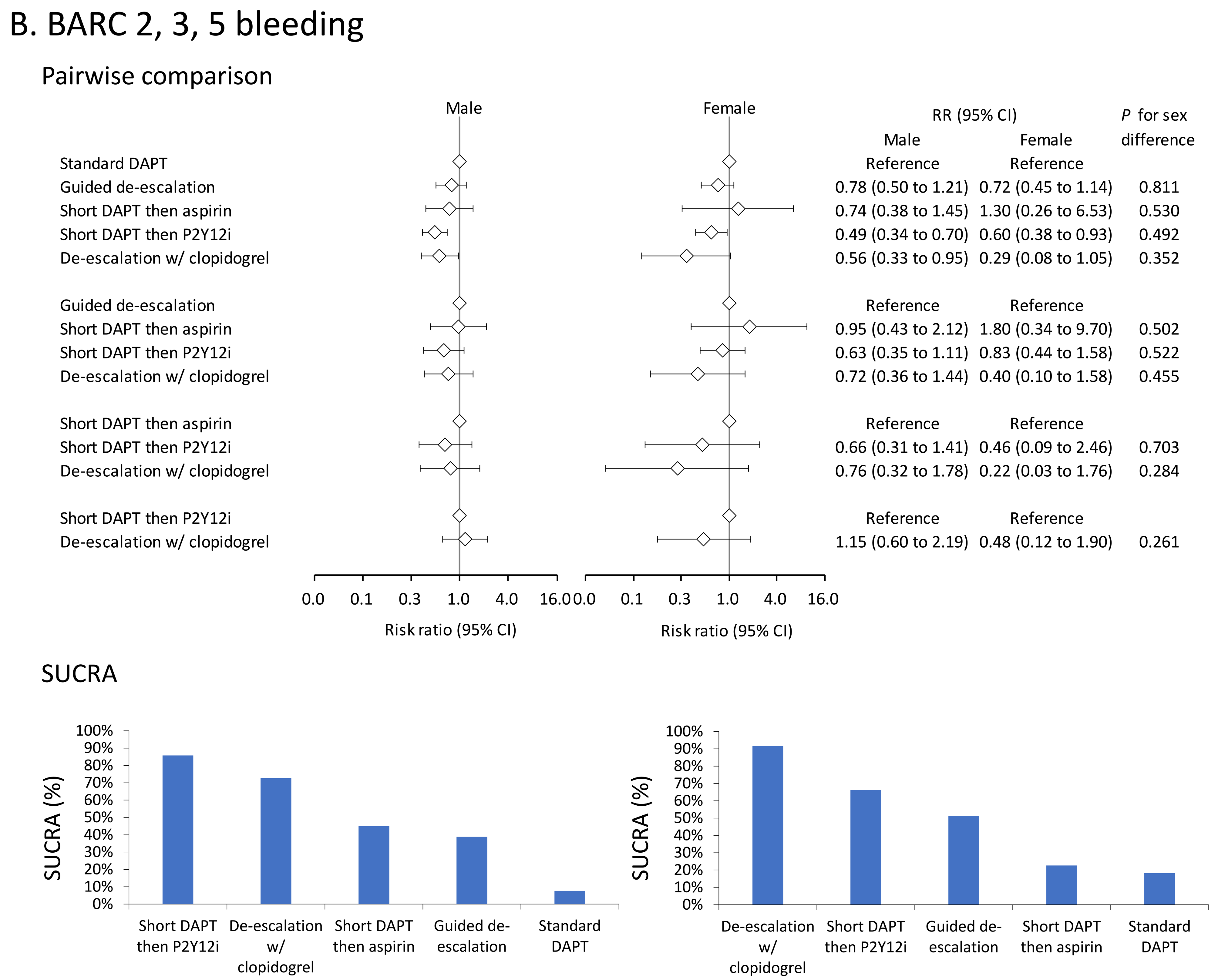

Supplement: Supplementary file 10 — Supplementary Material 10 [file 13293_2026_903_MOESM10_ESM.tif]

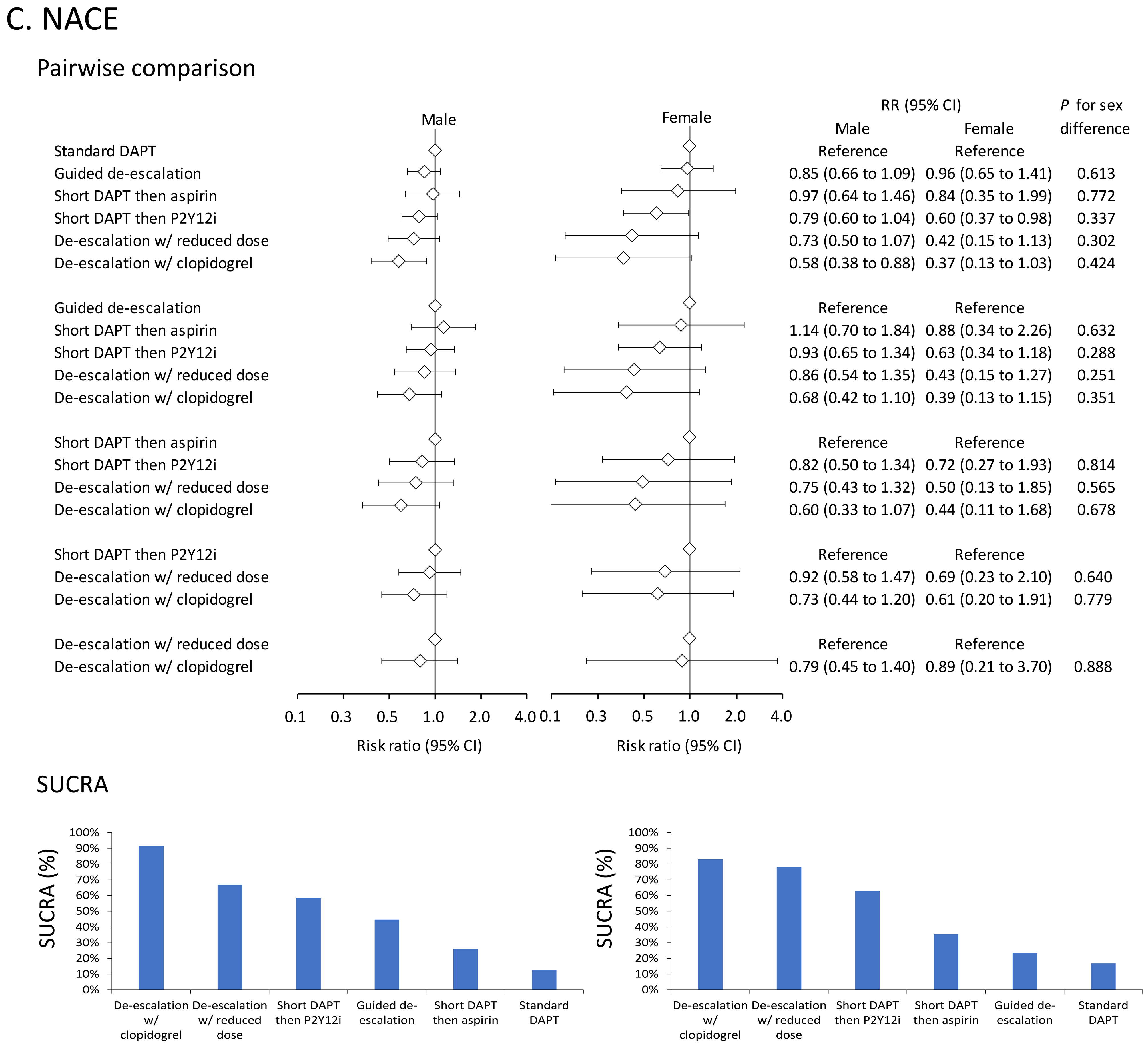

Supplement: Supplementary file 11 — Supplementary Material 11 [file 13293_2026_903_MOESM11_ESM.tif]

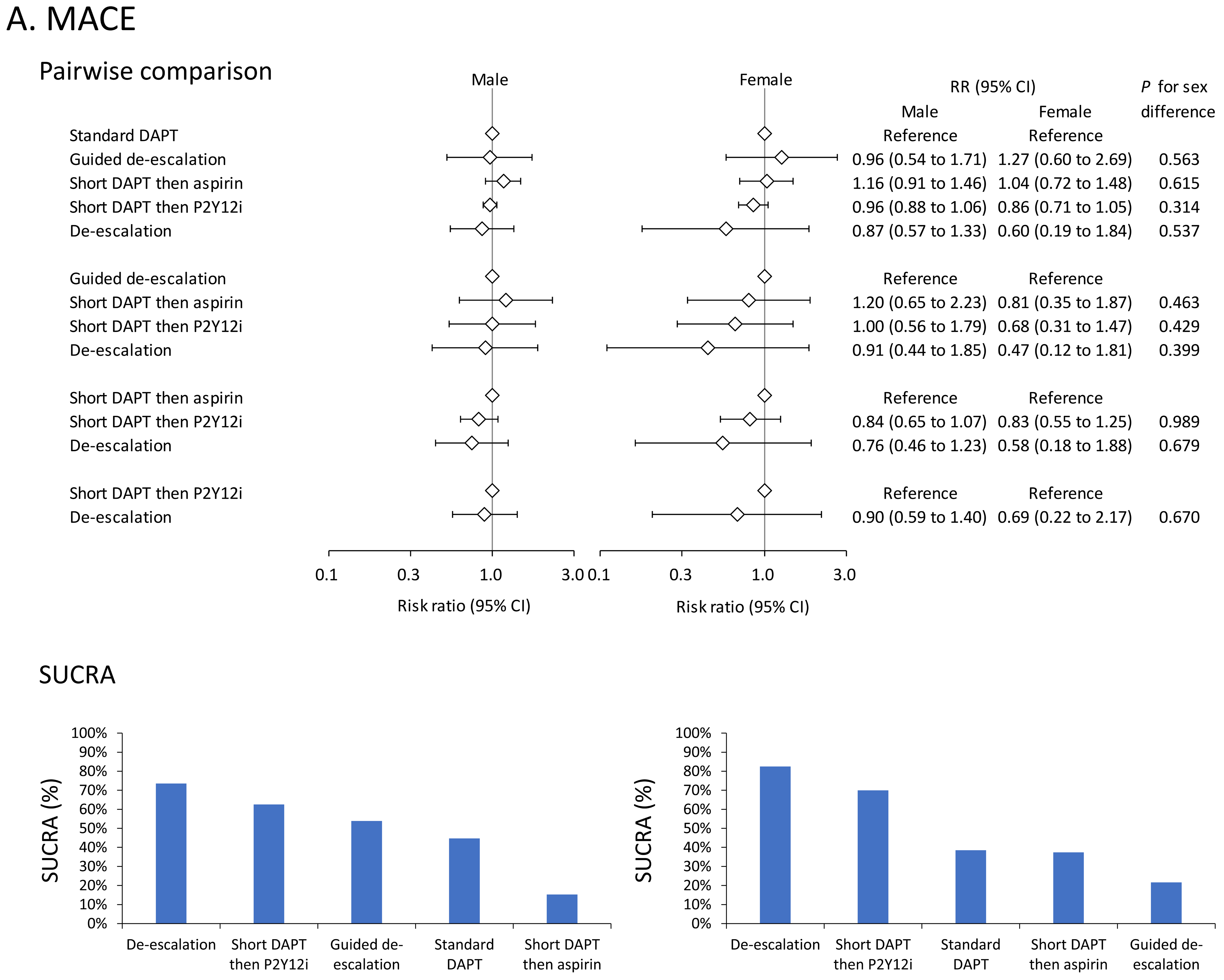

Supplement: Supplementary file 12 — Supplementary Material 12: Fig. S3. Forest plot and SUCRA of the network meta-analysis of MACE (A), BARC 2, 3, 5 bleeding (B), and NACE (C) among patients receiving different dual antiplatelet therapy strategies following percutaneous coronary intervention, using a five-node network in which the clopidogrel de-escalation and reduced-dose P2Y12 inhibitor categories were combined into a single “De-escalation” node. Supplemental BARC, Bleeding Academic Research Consortium; CI, confidence interval; DAPT, dual antiplatelet therapy; MACE, major adverse cardiovascular events; NACE, net adverse clinical events; P2Y12i, P2Y12 receptor inhibitor; RR, risk ratio; SUCRA, surface under the cumulative ranking curve. [file 13293_2026_903_MOESM12_ESM.tif]

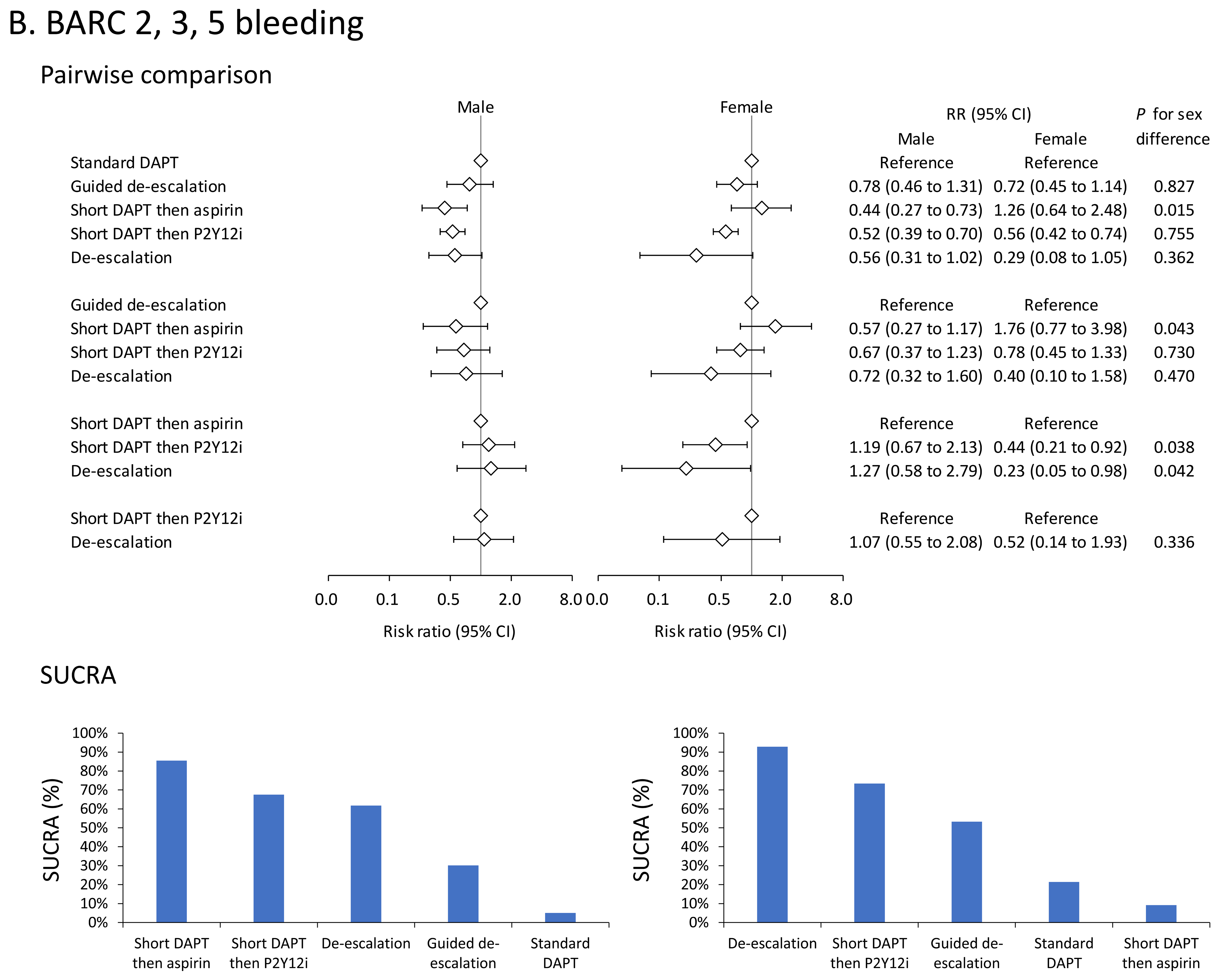

Supplement: Supplementary file 13 — Supplementary Material 13 [file 13293_2026_903_MOESM13_ESM.tif]

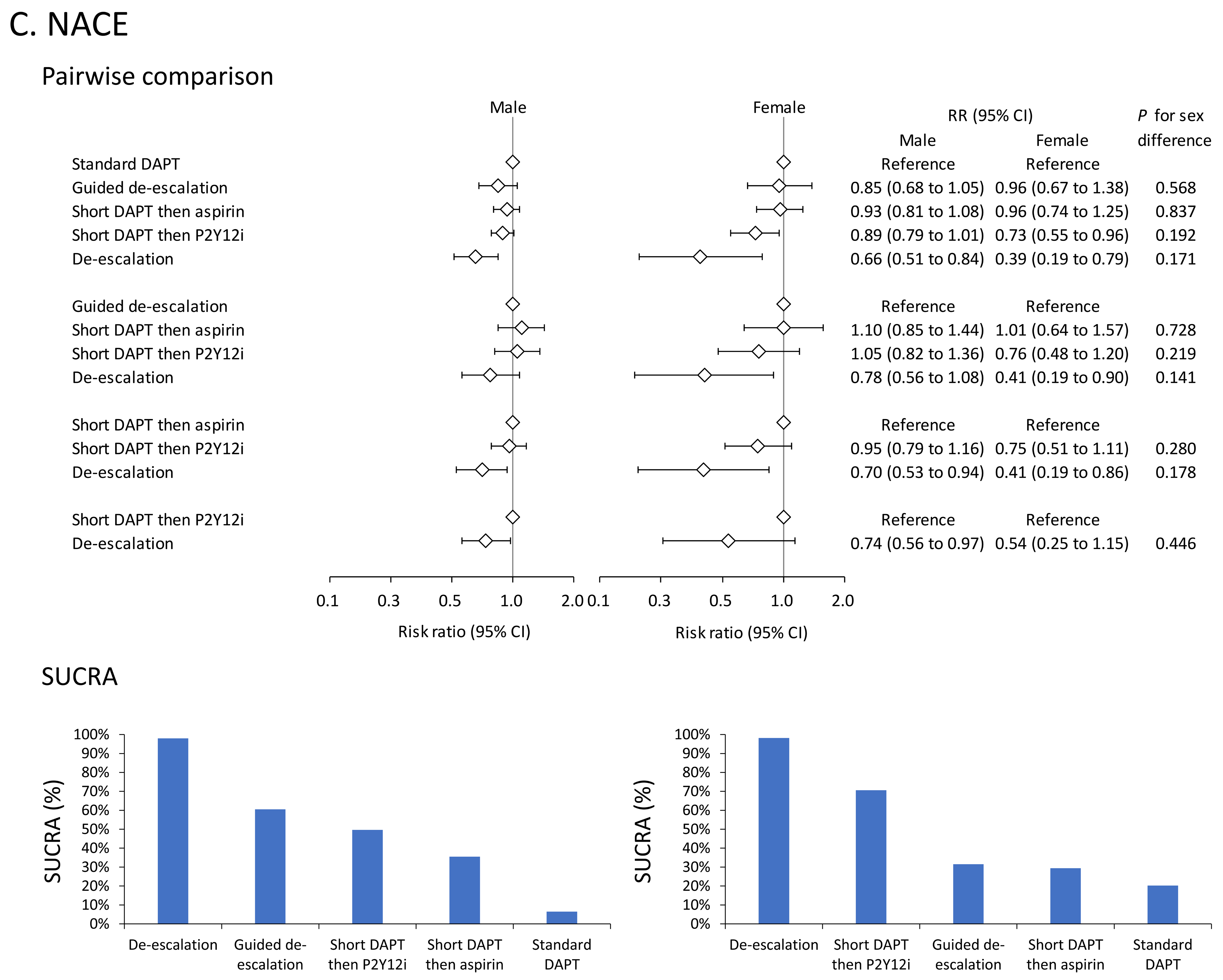

Supplement: Supplementary file 14 — Supplementary Material 14 [file 13293_2026_903_MOESM14_ESM.tif]

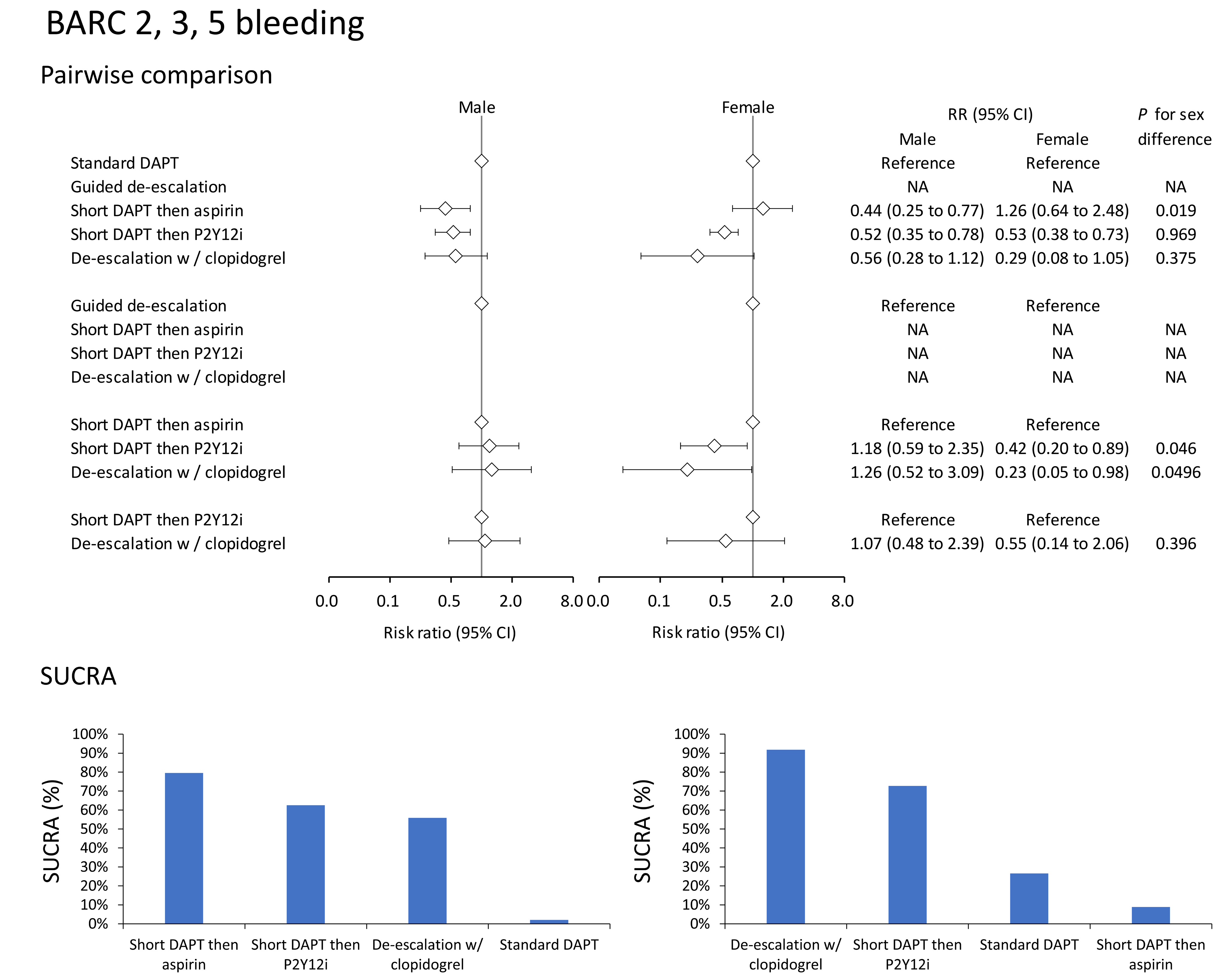

Supplement: Supplementary file 15 — Supplementary Material 15: Fig. S4. Forest plot and SUCRA of the network meta-analysis of BARC 2, 3, 5 bleeding among patients receiving different dual antiplatelet therapy strategies following percutaneous coronary intervention, restricted to trials using Bleeding Academic Research Consortium (BARC) criteria for bleeding ascertainment. BARC, Bleeding Academic Research Consortium; CI, confidence interval; DAPT, dual antiplatelet therapy; MACE, major adverse cardiovascular events; NACE, net adverse clinical events; P2Y12i, P2Y12 receptor inhibitor; RR, risk ratio; SUCRA, surface under the cumulative ranking curve. [file 13293_2026_903_MOESM15_ESM.tif]

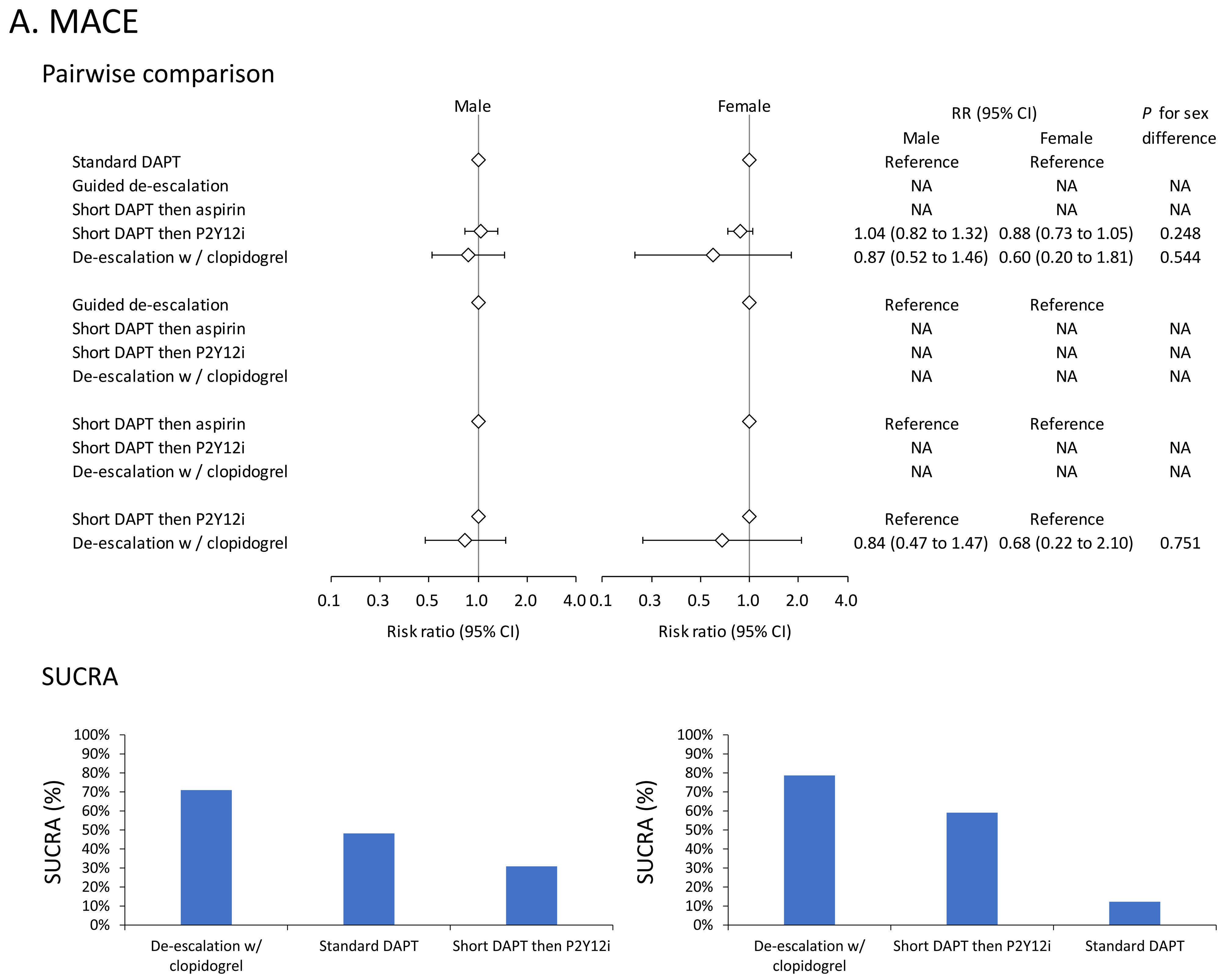

Supplement: Supplementary file 16 — Supplementary Material 16: Fig. S5. Forest plot and SUCRA of the network meta-analysis of MACE (A), BARC 2, 3, 5 bleeding (B), and NACE (C) among patients receiving different dual antiplatelet therapy strategies following percutaneous coronary intervention, restricted to trials with a therapy switch timing of 3–5 months. BARC, Bleeding Academic Research Consortium; CI, confidence interval; DAPT, dual antiplatelet therapy; MACE, major adverse cardiovascular events; NACE, net adverse clinical events; P2Y12i, P2Y12 receptor inhibitor; RR, risk ratio; SUCRA, surface under the cumulative ranking curve. [file 13293_2026_903_MOESM16_ESM.tif]

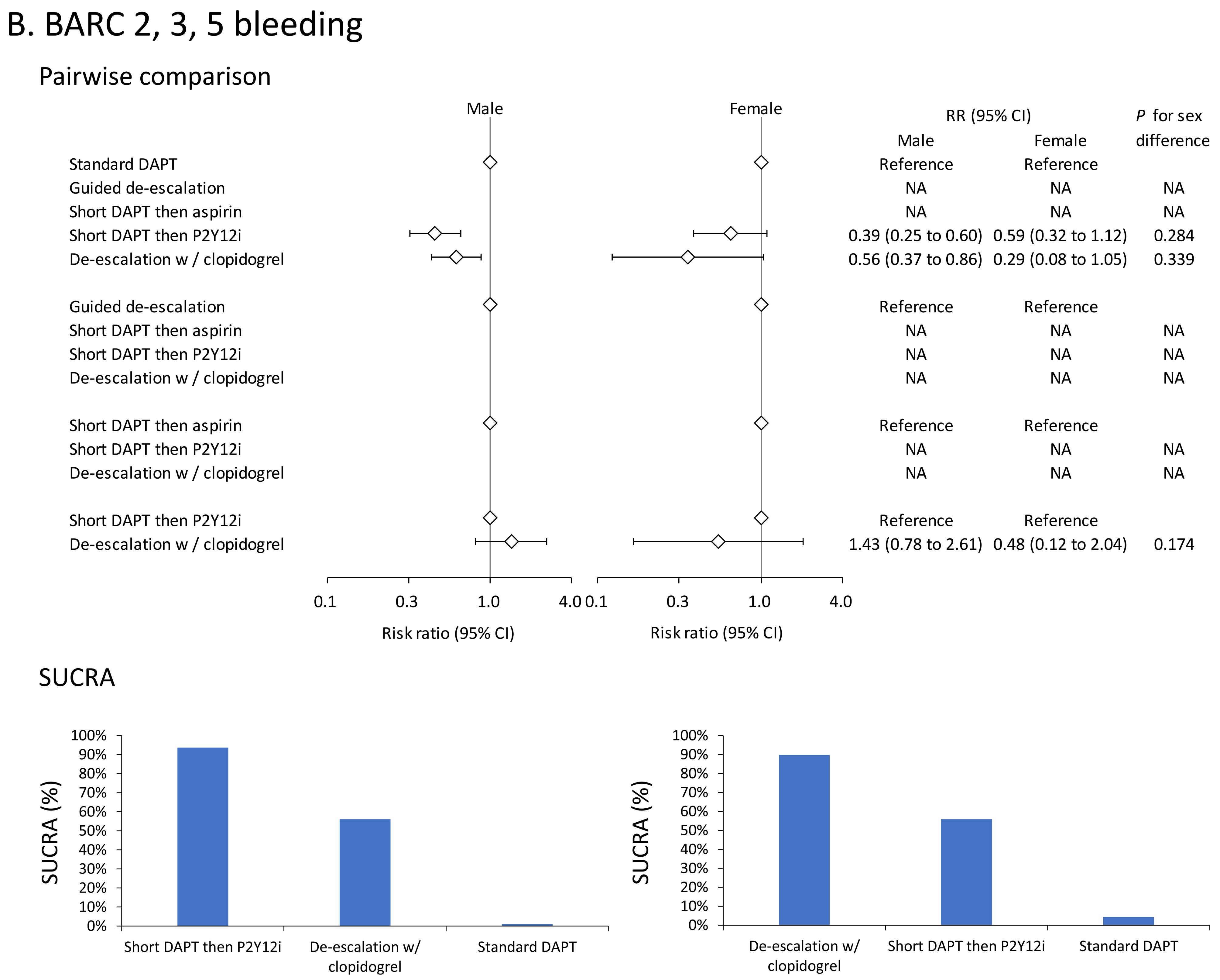

Supplement: Supplementary file 17 — Supplementary Material 17 [file 13293_2026_903_MOESM17_ESM.tif]

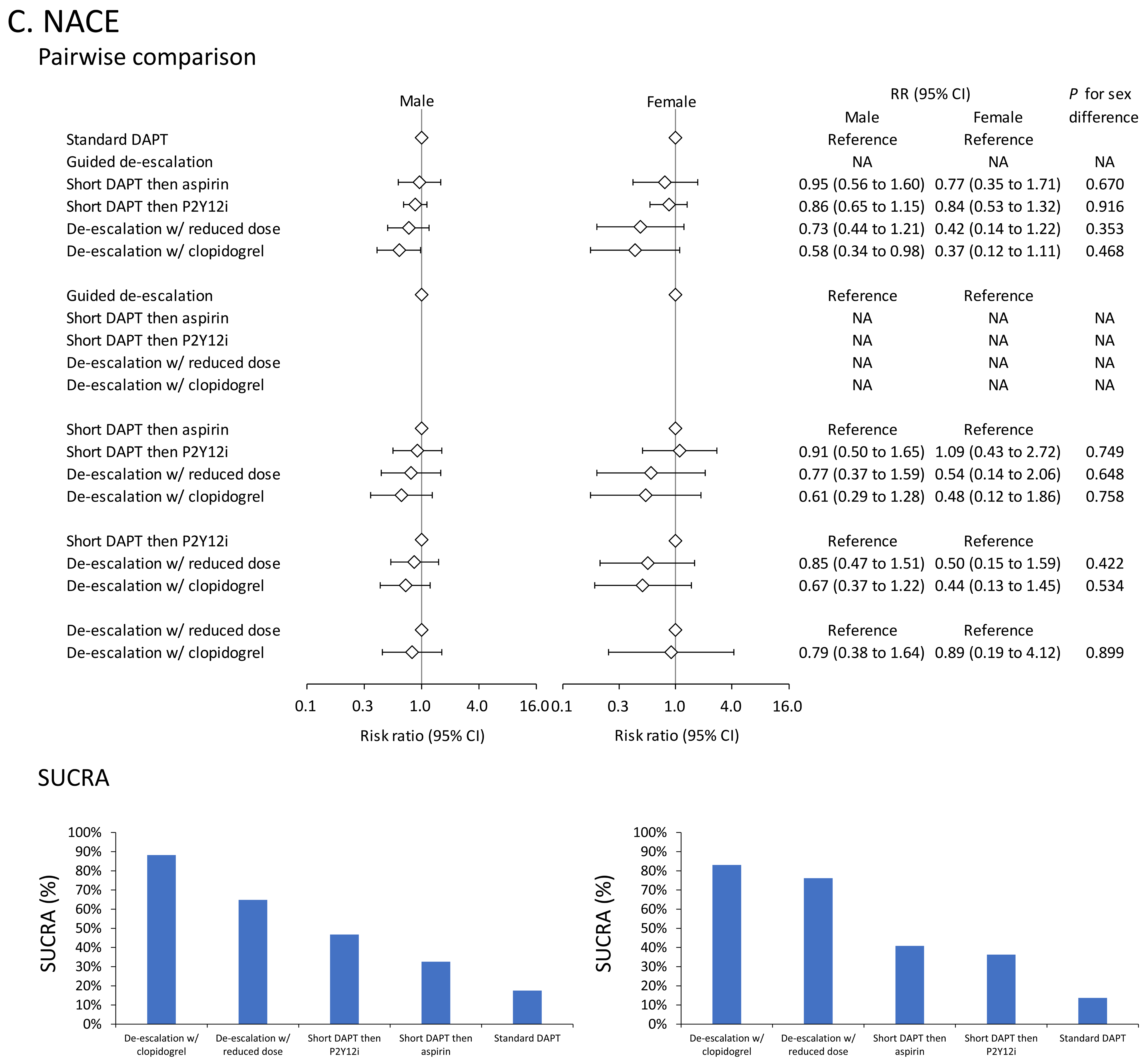

Supplement: Supplementary file 18 — Supplementary Material 18 [file 13293_2026_903_MOESM18_ESM.tif]

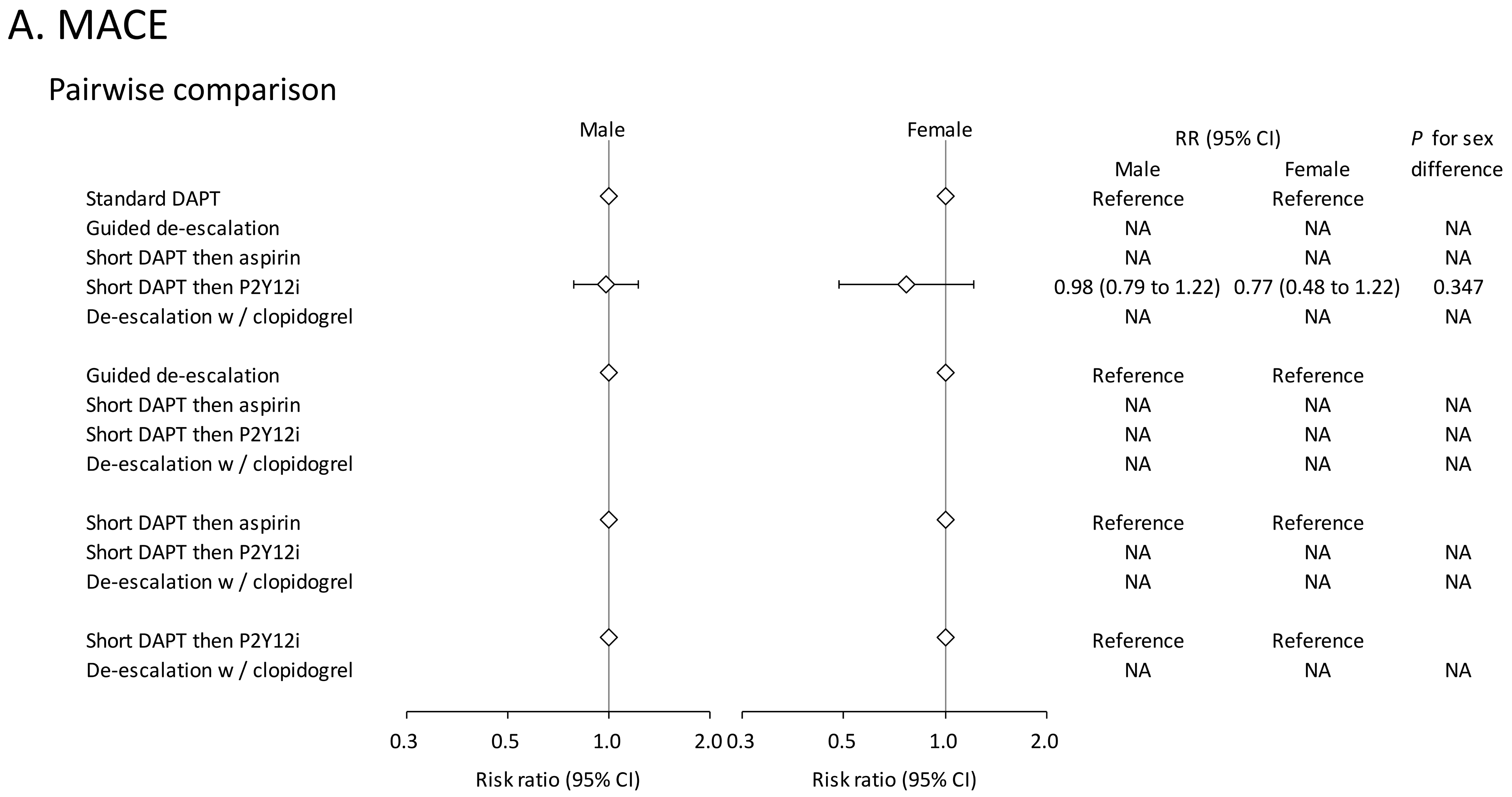

Supplement: Supplementary file 19 — Supplementary Material 19: Fig. S6. Forest plot and SUCRA of the network meta-analysis of MACE (A), BARC 2, 3, 5 bleeding (B), and NACE (C) among patients receiving different dual antiplatelet therapy strategies following percutaneous coronary intervention, restricted to trials with a therapy switch timing of less than 3 months. BARC, Bleeding Academic Research Consortium; CI, confidence interval; DAPT, dual antiplatelet therapy; MACE, major adverse cardiovascular events; NACE, net adverse clinical events; P2Y12i, P2Y12 receptor inhibitor; RR, risk ratio; SUCRA, surface under the cumulative ranking curve. [file 13293_2026_903_MOESM19_ESM.tif]

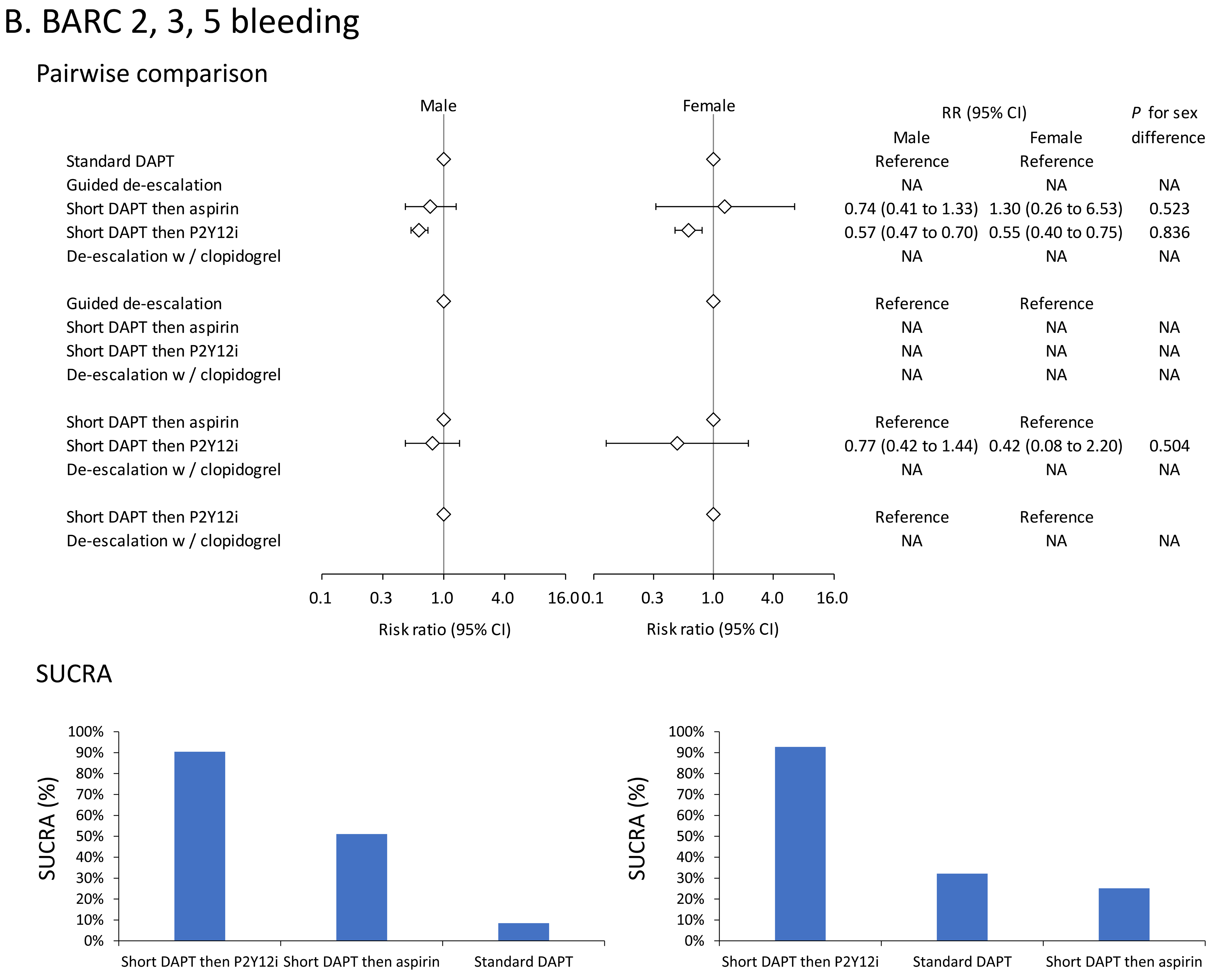

Supplement: Supplementary file 20 — Supplementary Material 20 [file 13293_2026_903_MOESM20_ESM.tif]

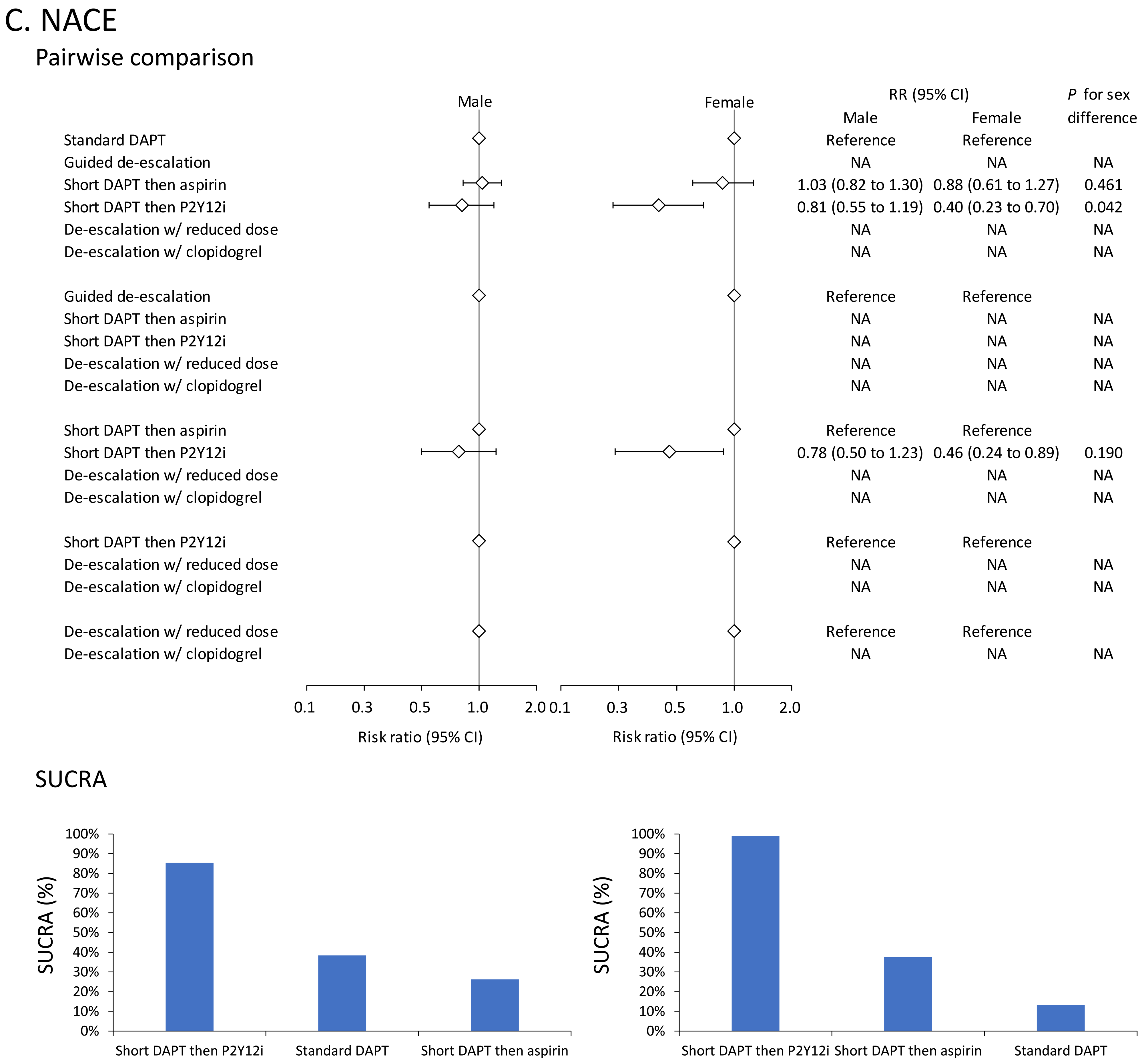

Supplement: Supplementary file 21 — Supplementary Material 21 [file 13293_2026_903_MOESM21_ESM.tif]

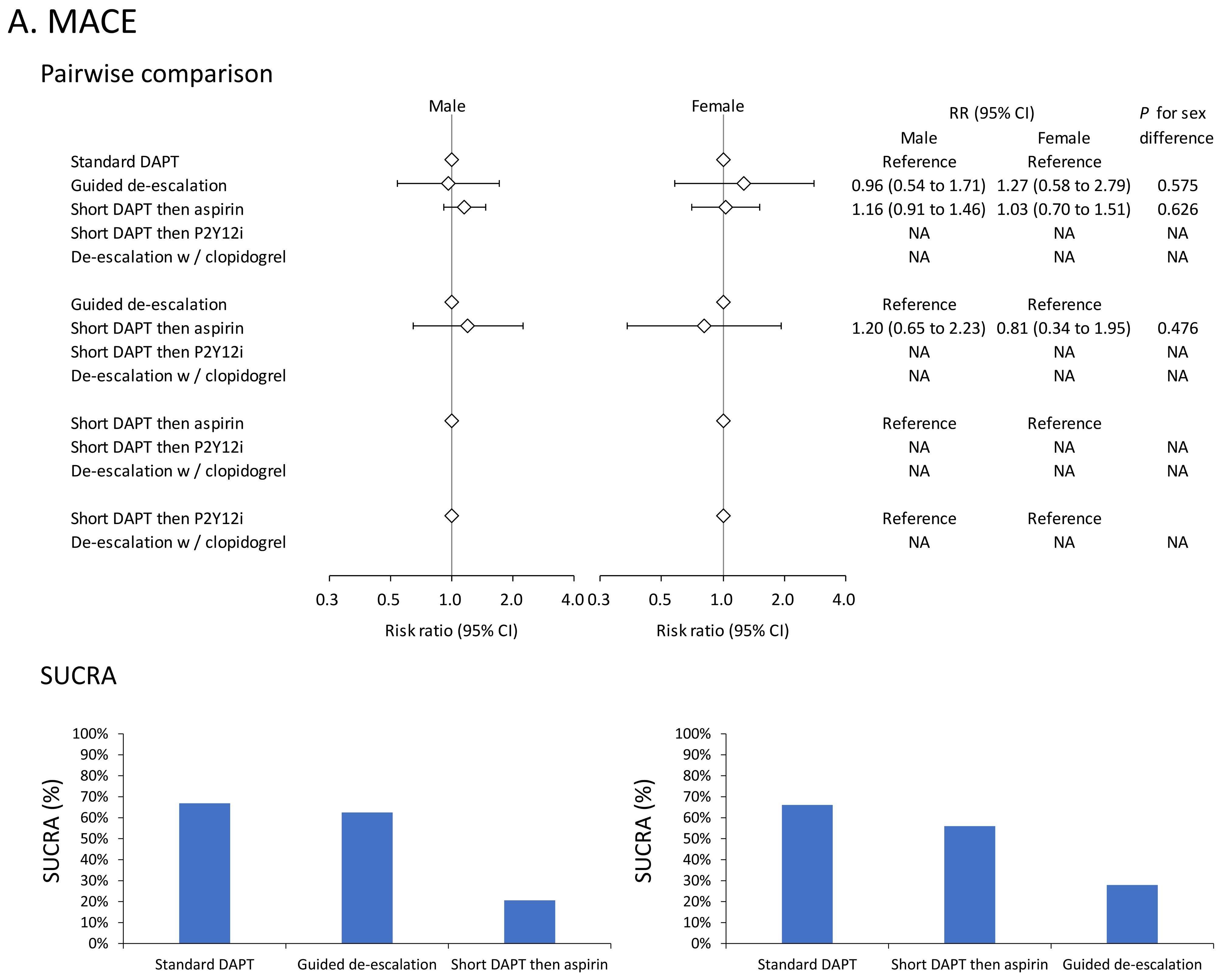

Supplement: Supplementary file 22 — Supplementary Material 22: Fig. S7. Forest plot and SUCRA of the network meta-analysis of MACE (A), BARC 2, 3, 5 bleeding (B), and NACE (C) among patients receiving different dual antiplatelet therapy strategies following percutaneous coronary intervention, restricted to trials with a therapy switch timing of 6 months or greater. BARC, Bleeding Academic Research Consortium; CI, confidence interval; DAPT, dual antiplatelet therapy; MACE, major adverse cardiovascular events; NACE, net adverse clinical events; P2Y12i, P2Y12 receptor inhibitor; RR, risk ratio; SUCRA, surface under the cumulative ranking curve. [file 13293_2026_903_MOESM22_ESM.tif]

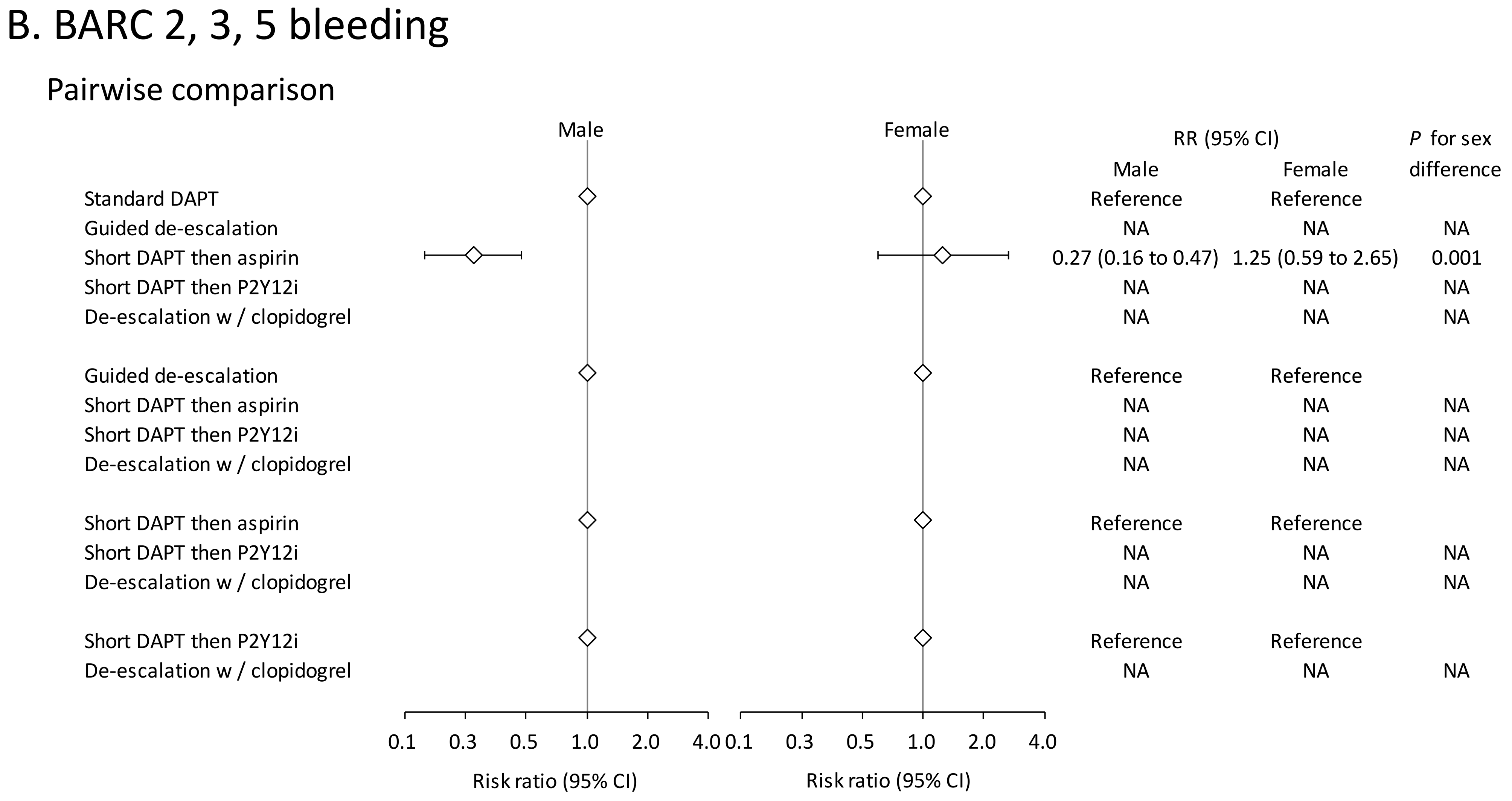

Supplement: Supplementary file 23 — Supplementary Material 23 [file 13293_2026_903_MOESM23_ESM.tif]

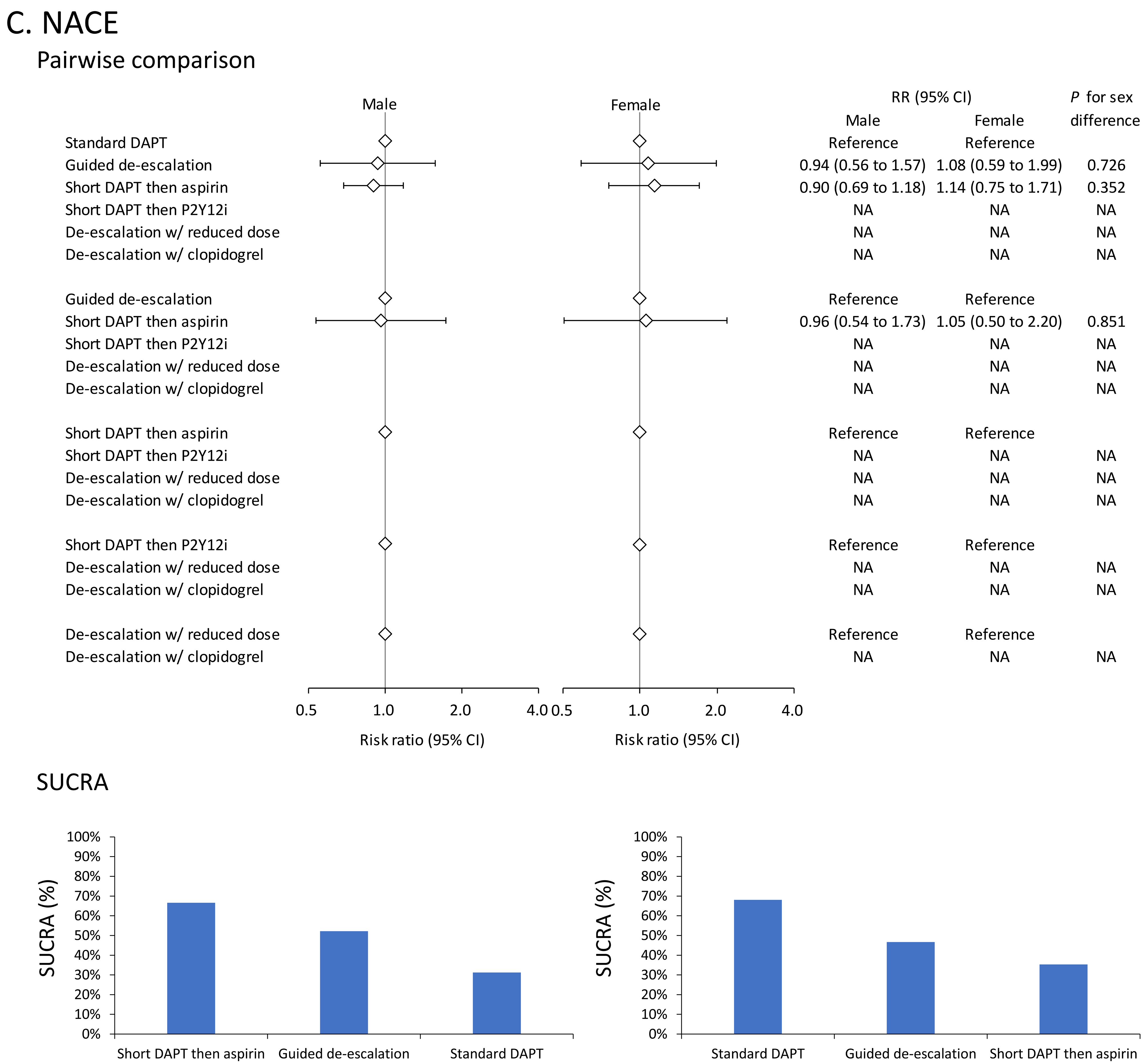

Supplement: Supplementary file 24 — Supplementary Material 24 [file 13293_2026_903_MOESM24_ESM.tif]

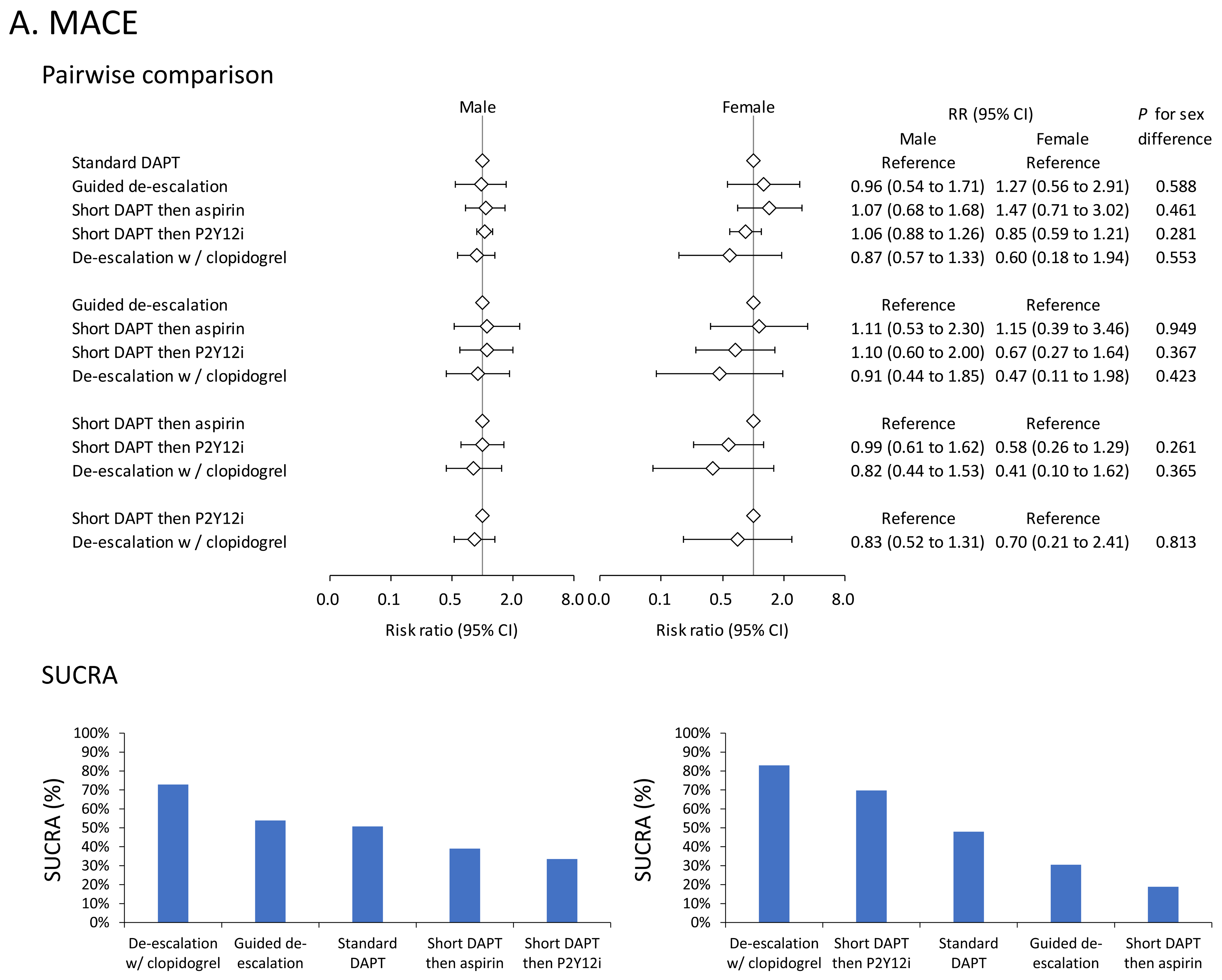

Supplement: Supplementary file 25 — Supplementary Material 25: Fig. S8. Forest plot and SUCRA of the network meta-analysis of MACE (A), BARC 2, 3, 5 bleeding (B), and NACE (C) among patients receiving different dual antiplatelet therapy strategies following percutaneous coronary intervention, restricted to trials with a follow-up duration of 12 months or less. BARC, Bleeding Academic Research Consortium; CI, confidence interval; DAPT, dual antiplatelet therapy; MACE, major adverse cardiovascular events; NACE, net adverse clinical events; P2Y12i, P2Y12 receptor inhibitor; RR, risk ratio; SUCRA, surface under the cumulative ranking curve. [file 13293_2026_903_MOESM25_ESM.tif]

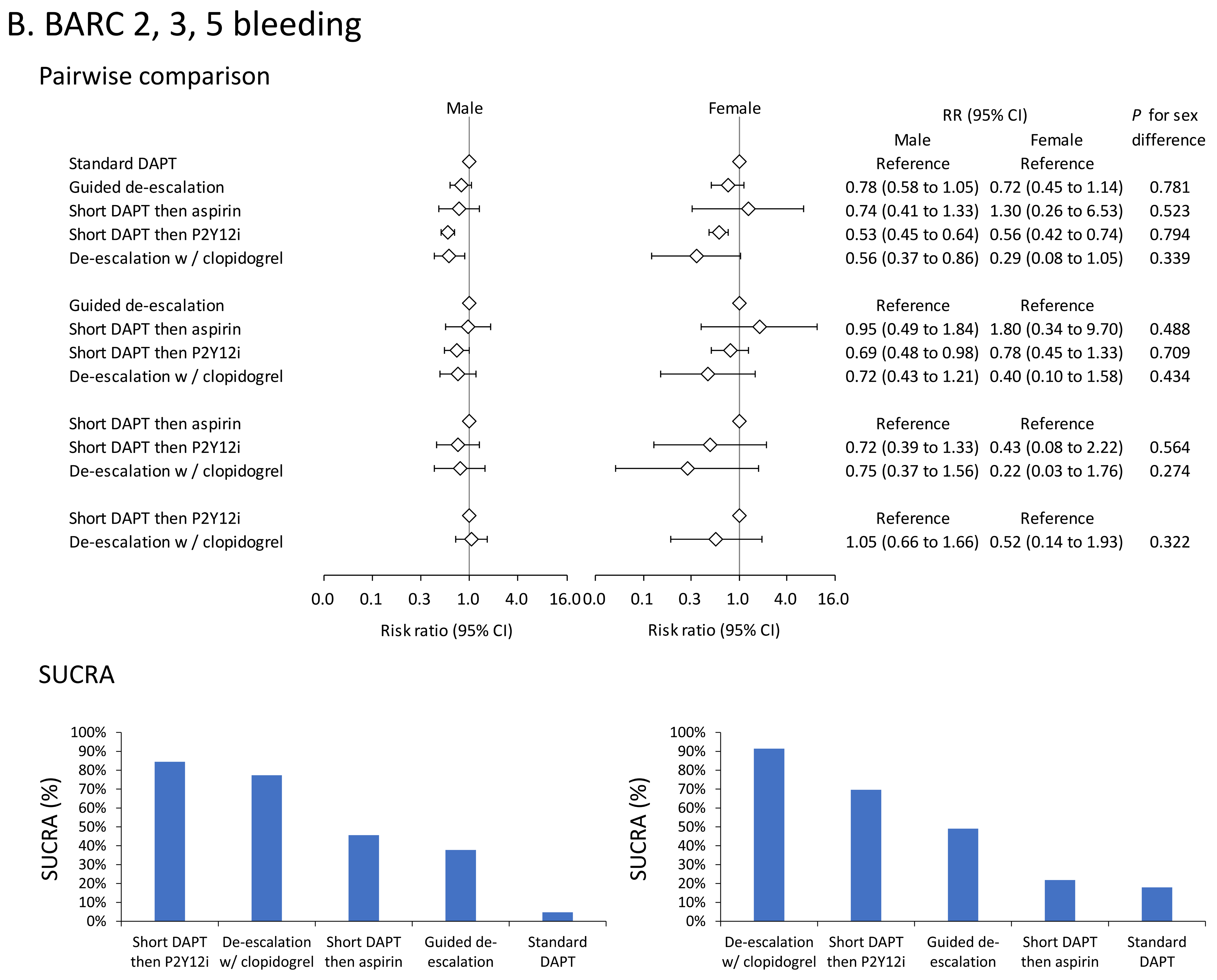

Supplement: Supplementary file 26 — Supplementary Material 26 [file 13293_2026_903_MOESM26_ESM.tif]

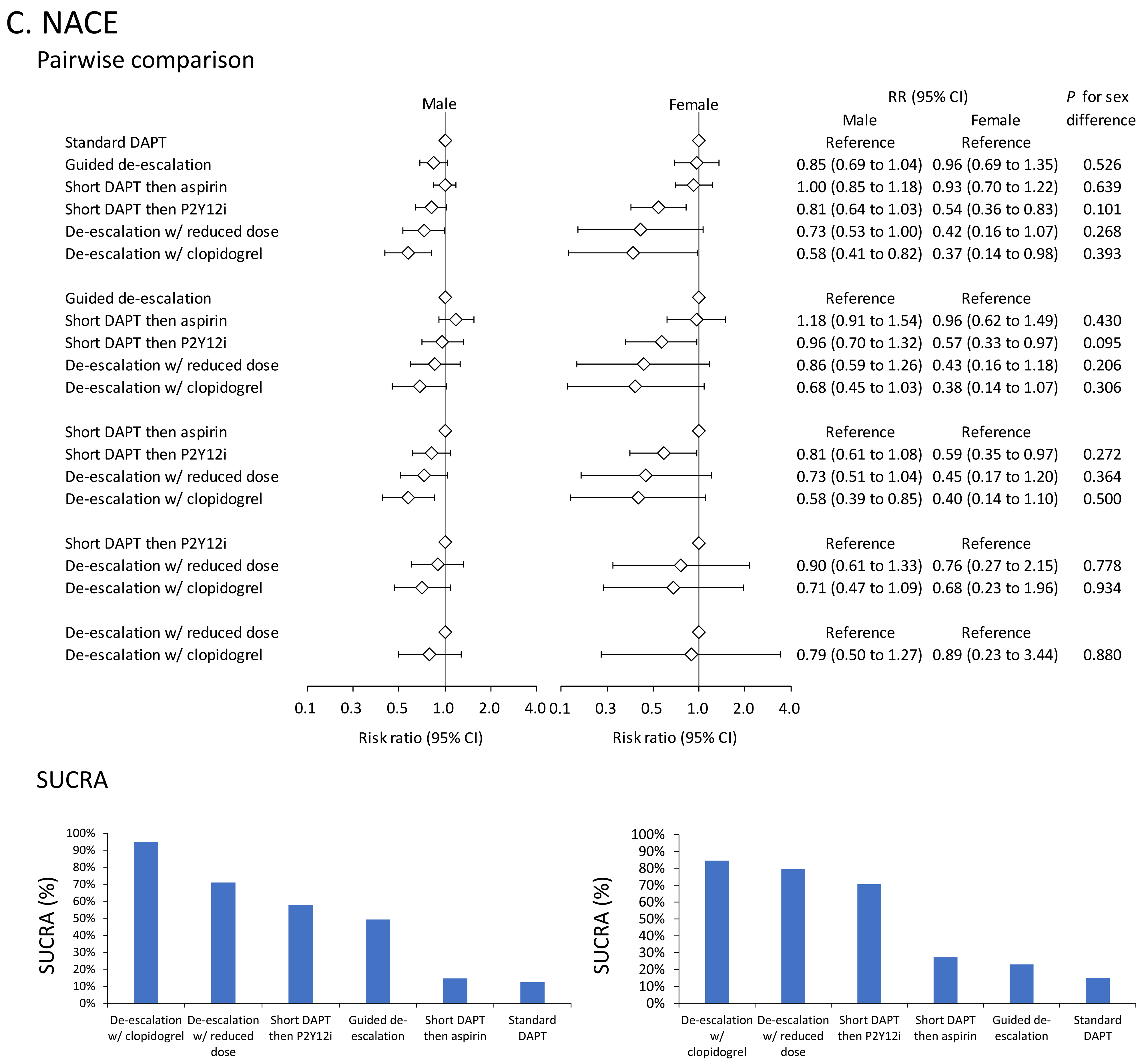

Supplement: Supplementary file 27 — Supplementary Material 27 [file 13293_2026_903_MOESM27_ESM.tif]

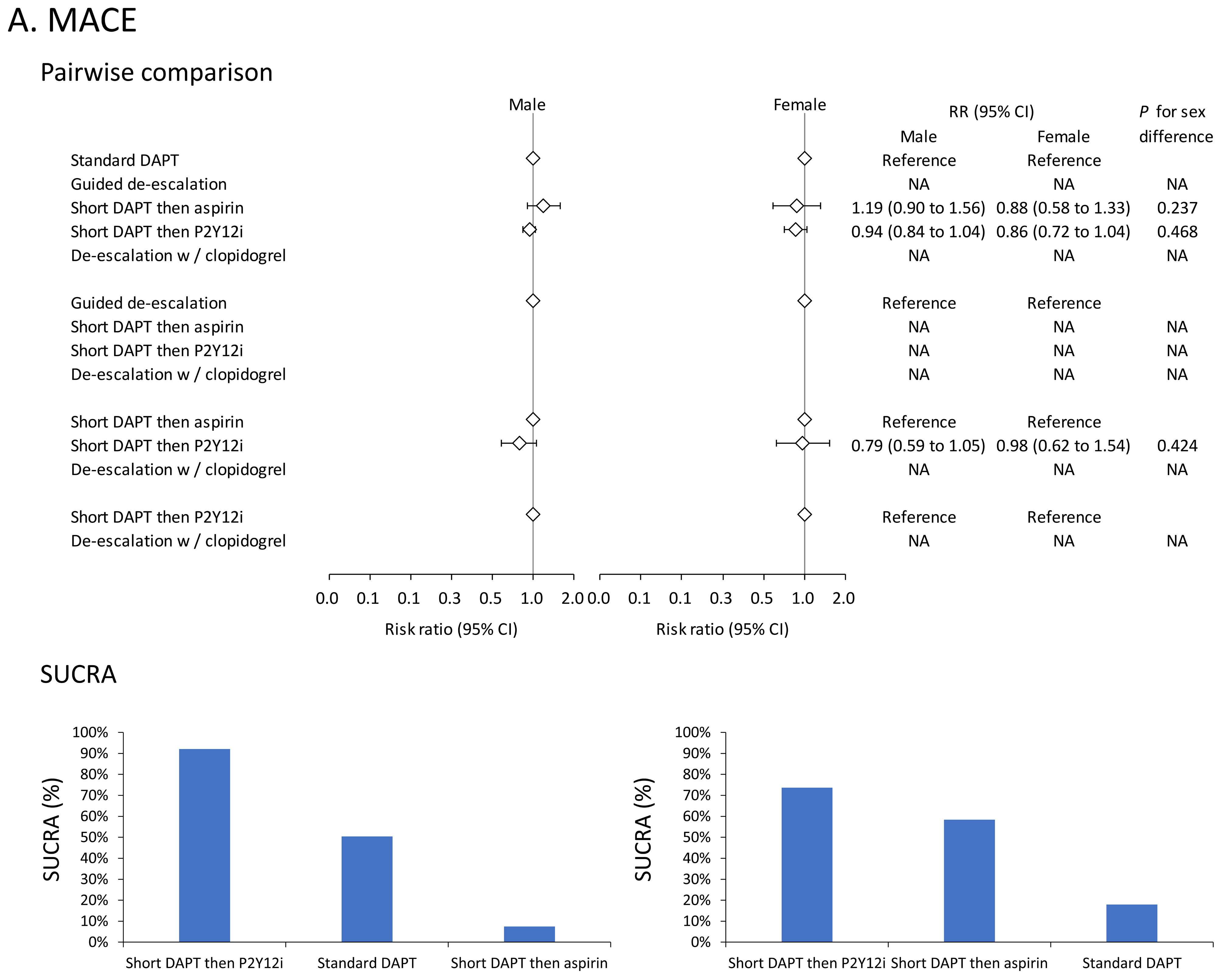

Supplement: Supplementary file 28 — Supplementary Material 28: Fig. S9. Forest plot and SUCRA of the network meta-analysis of MACE (A), BARC 2, 3, 5 bleeding (B), and NACE (C) among patients receiving different dual antiplatelet therapy strategies following percutaneous coronary intervention, restricted to trials with a follow-up duration of more than 12 months. BARC, Bleeding Academic Research Consortium; CI, confidence interval; DAPT, dual antiplatelet therapy; MACE, major adverse cardiovascular events; NACE, net adverse clinical events; P2Y12i, P2Y12 receptor inhibitor; RR, risk ratio; SUCRA, surface under the cumulative ranking curve. [file 13293_2026_903_MOESM28_ESM.tif]

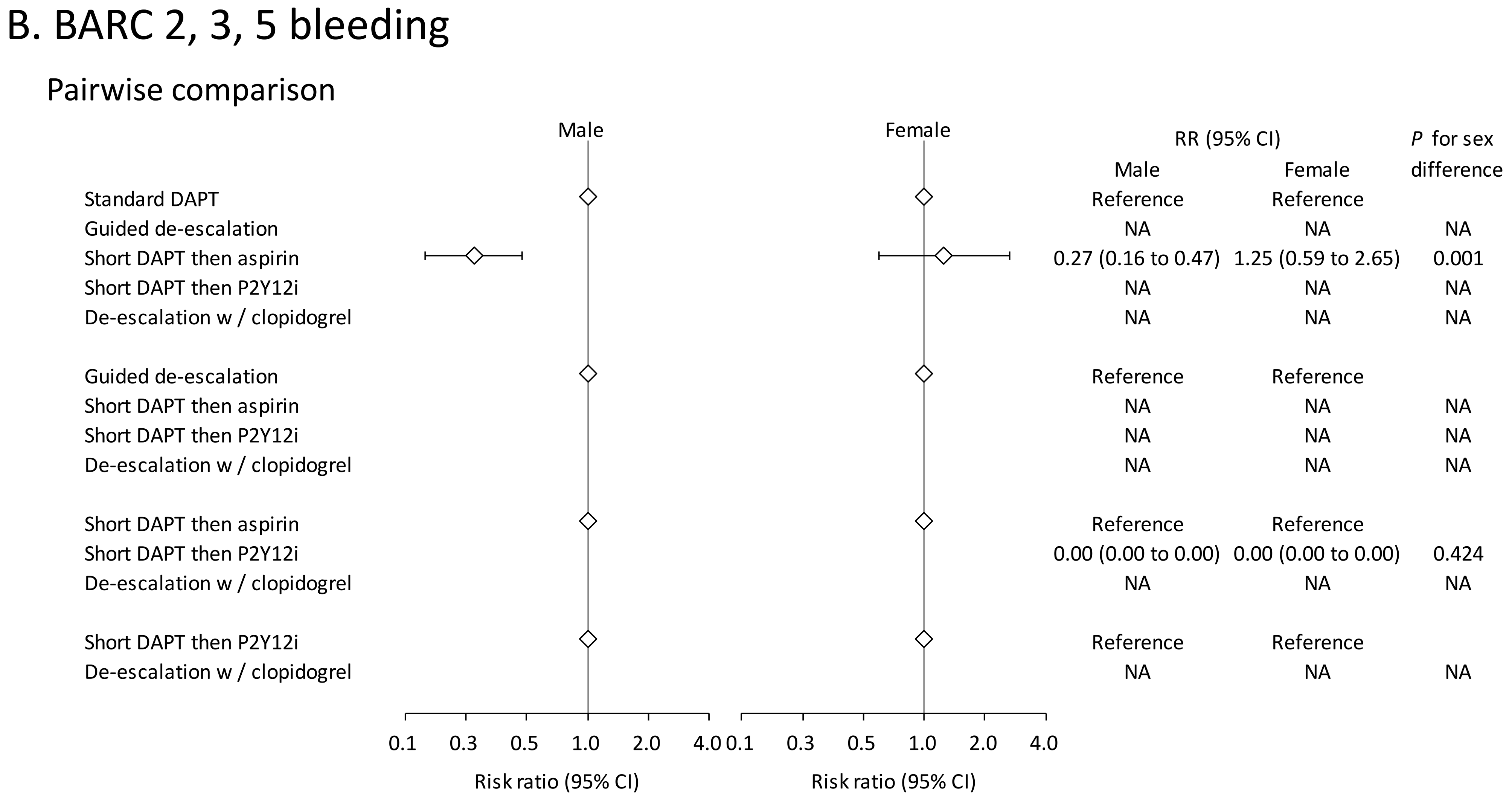

Supplement: Supplementary file 29 — Supplementary Material 29 [file 13293_2026_903_MOESM29_ESM.tif]

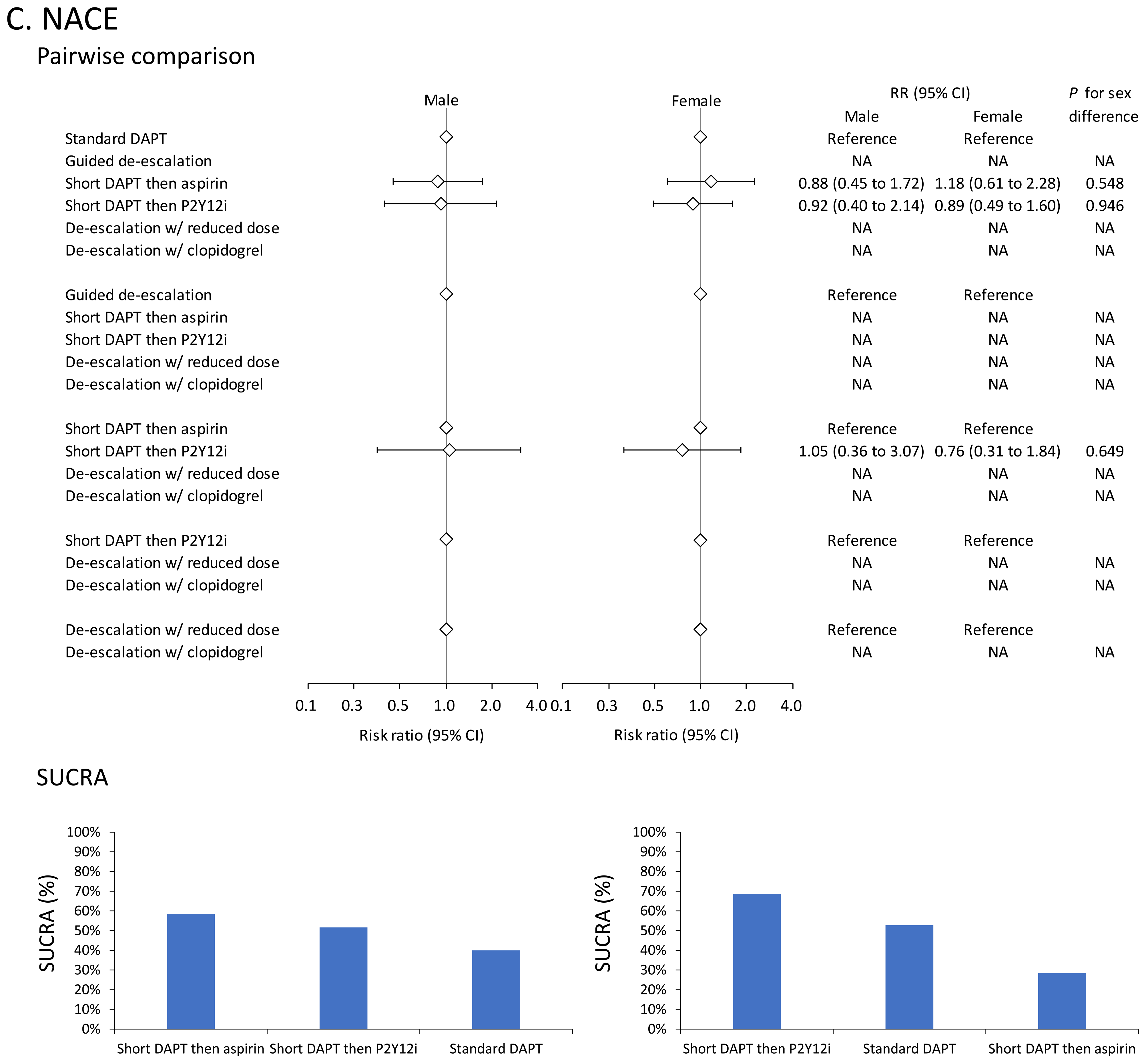

Supplement: Supplementary file 30 — Supplementary Material 30 [file 13293_2026_903_MOESM30_ESM.tif]

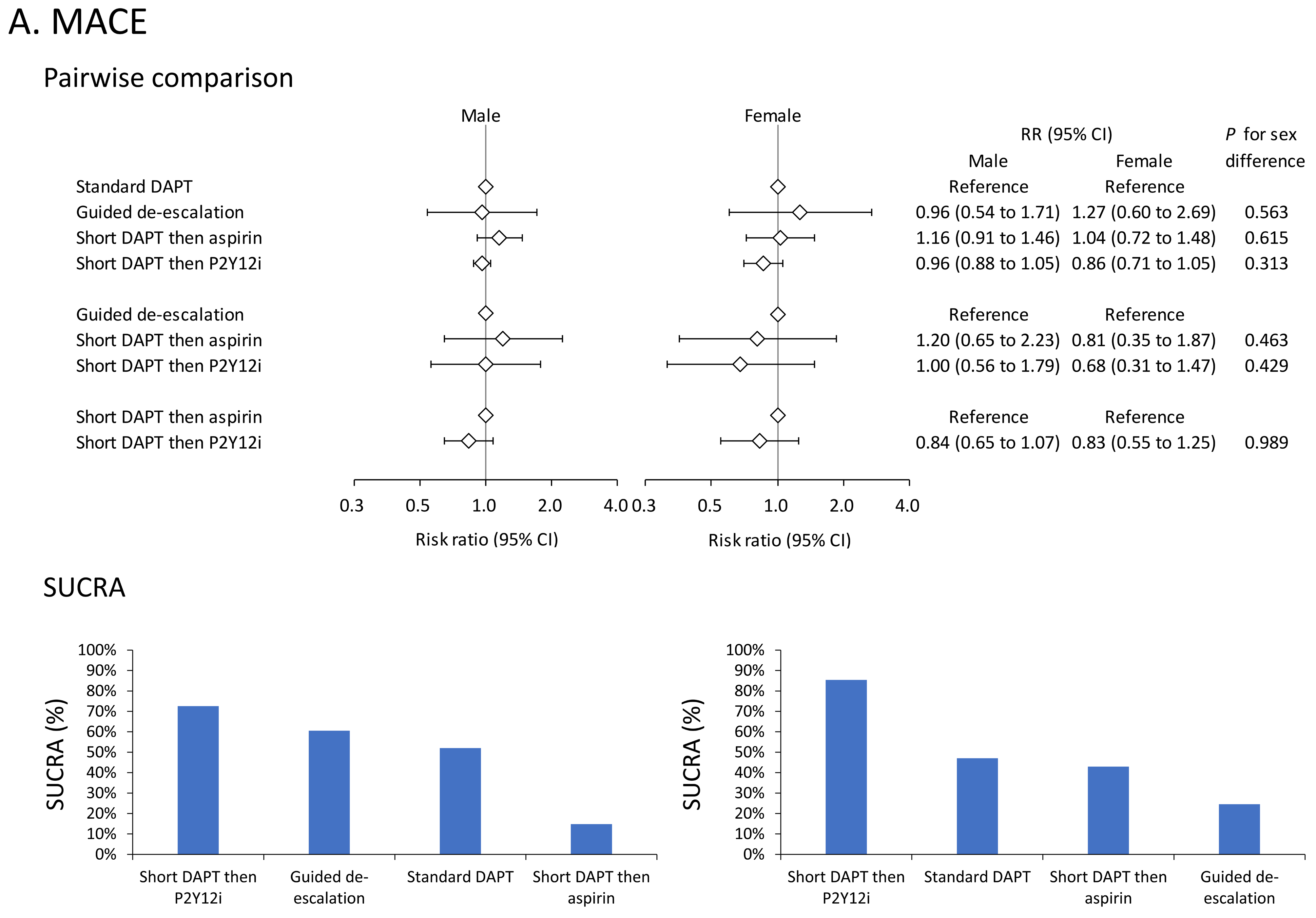

Supplement: Supplementary file 31 — Supplementary Material 31: Fig. S10. Forest plot and SUCRA of the network meta-analysis of MACE (A), BARC 2, 3, 5 bleeding (B), and NACE (C) among patients receiving different dual antiplatelet therapy strategies following percutaneous coronary intervention, after exclusion of the clopidogrel de-escalation node (TALOS-AMI), yielding a five-node network. BARC, Bleeding Academic Research Consortium; CI, confidence interval; DAPT, dual antiplatelet therapy; MACE, major adverse cardiovascular events; NACE, net adverse clinical events; P2Y12i, P2Y12 receptor inhibitor; RR, risk ratio; SUCRA, surface under the cumulative ranking curve. [file 13293_2026_903_MOESM31_ESM.tif]

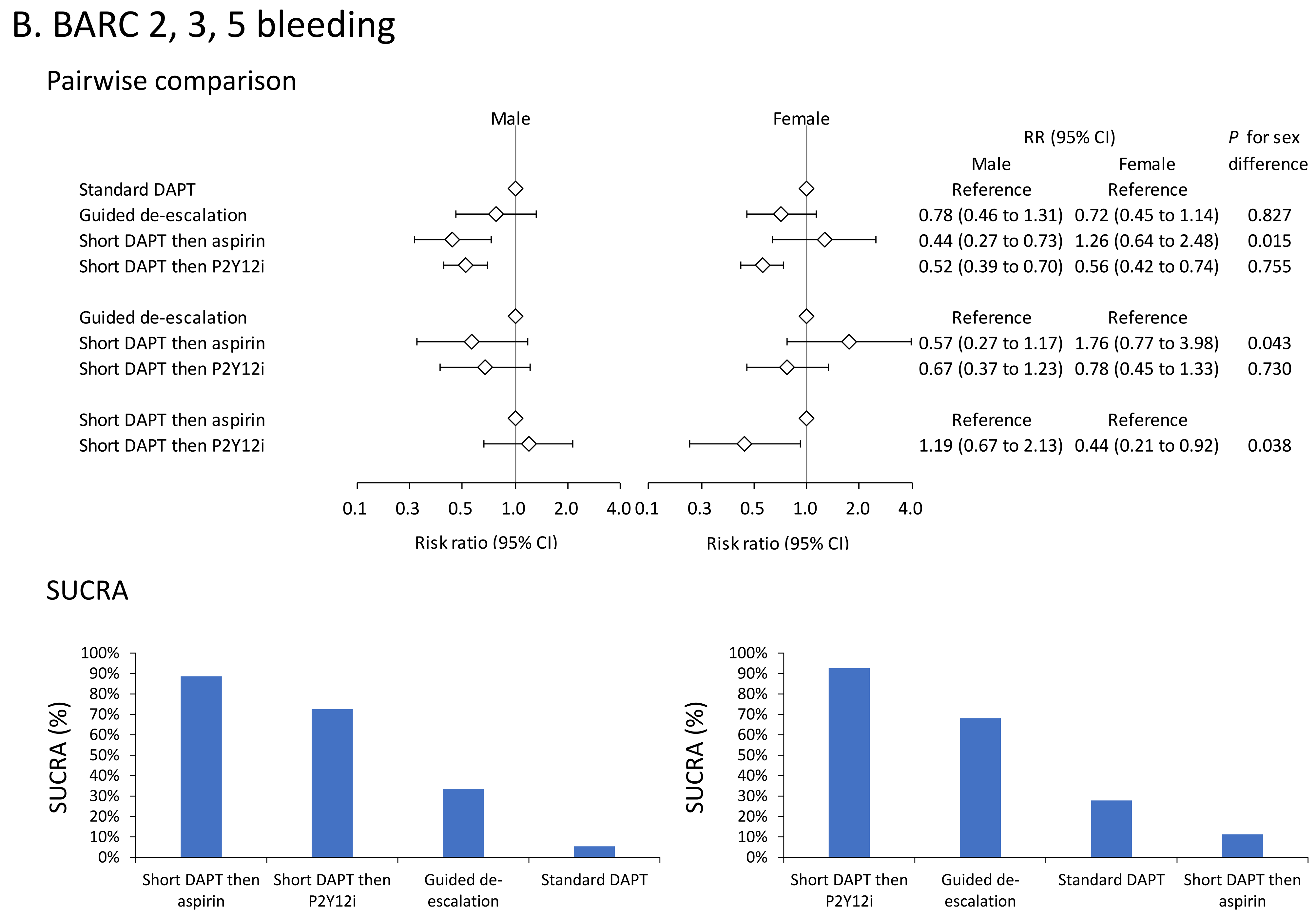

Supplement: Supplementary file 32 — Supplementary Material 32 [file 13293_2026_903_MOESM32_ESM.tif]

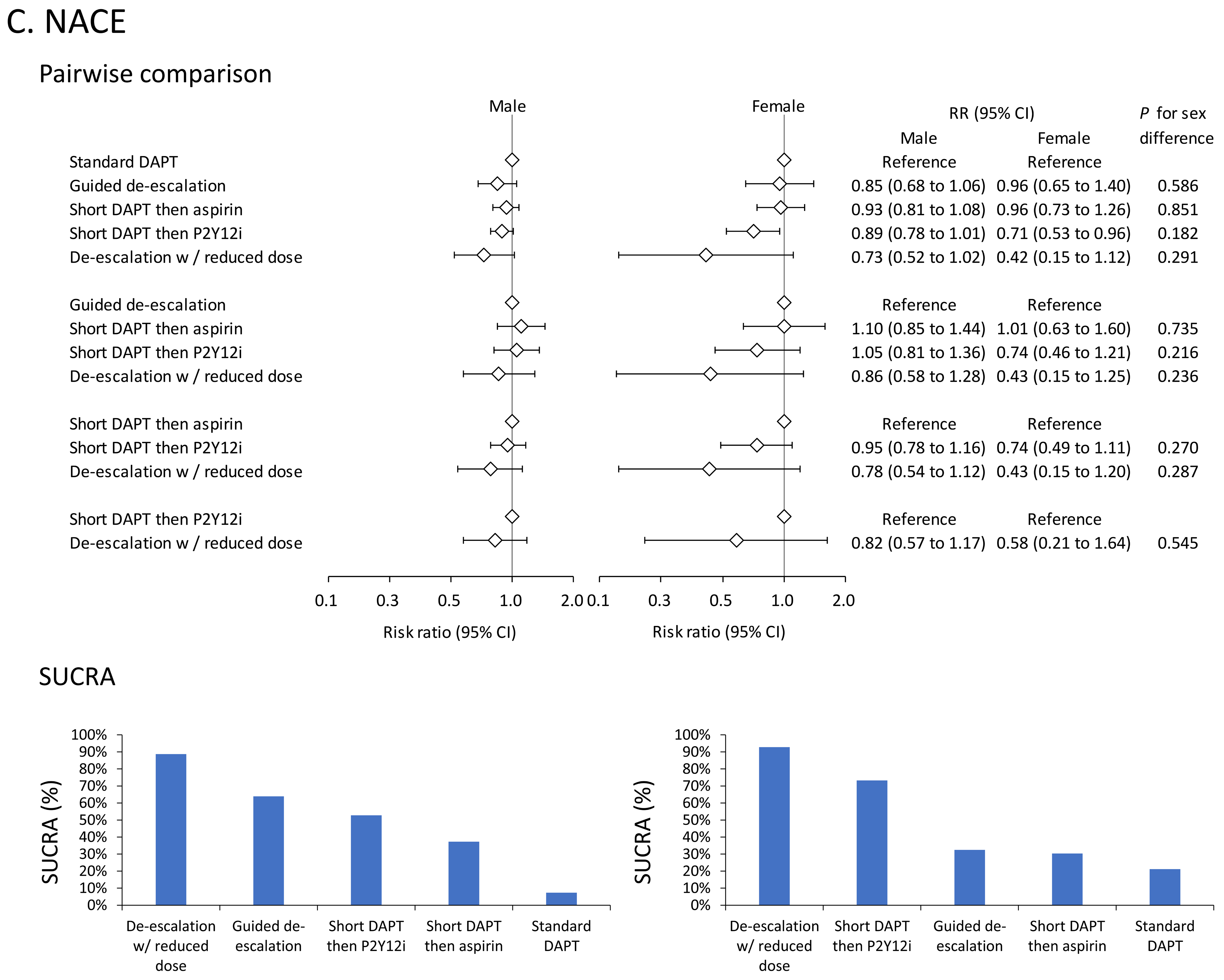

Supplement: Supplementary file 33 — Supplementary Material 33 [file 13293_2026_903_MOESM33_ESM.tif]

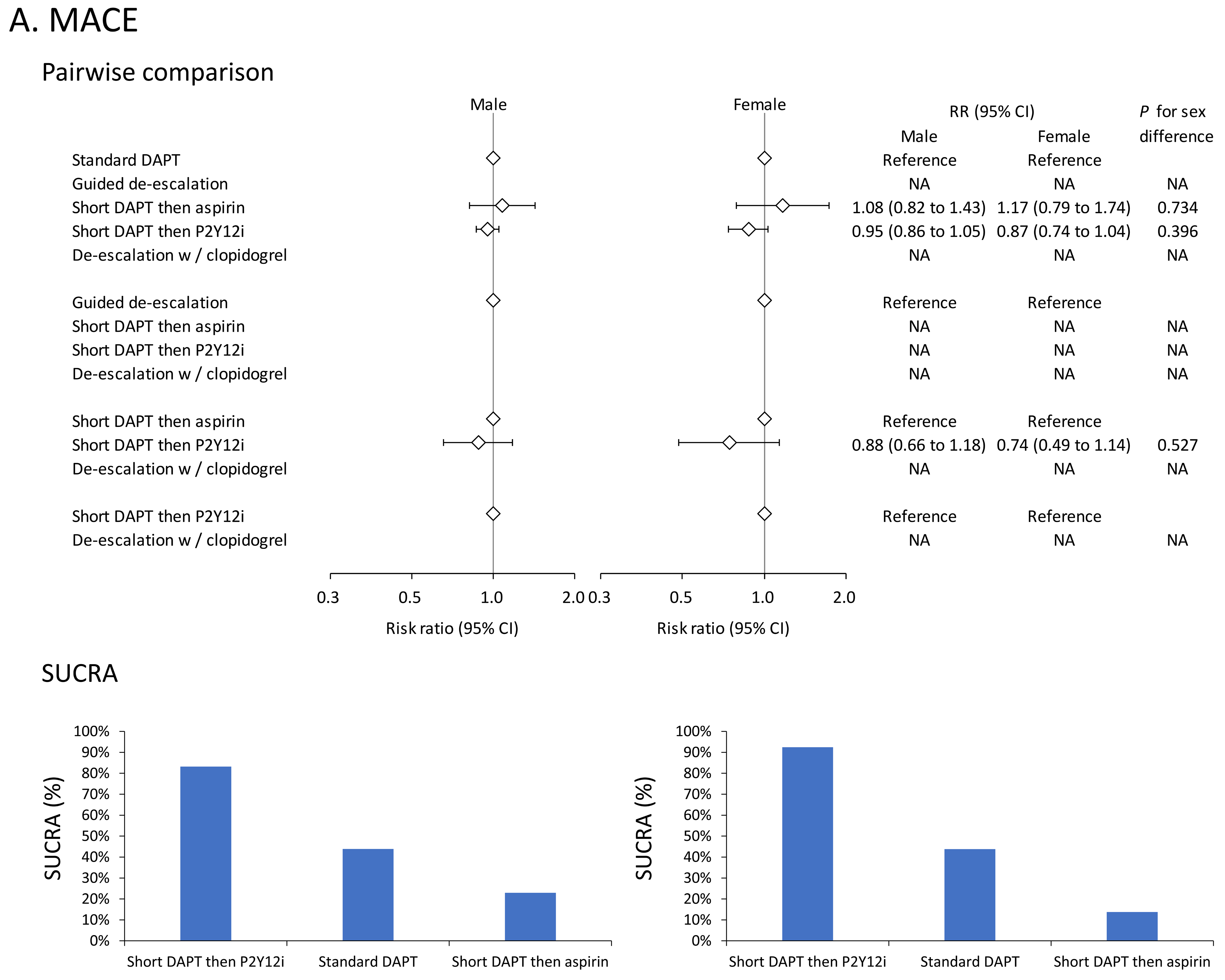

Supplement: Supplementary file 34 — Supplementary Material 34: Fig. S11. Forest plot and SUCRA of the network meta-analysis of MACE (A), BARC 2, 3, 5 bleeding (B), and NACE (C) among patients receiving different dual antiplatelet therapy strategies following percutaneous coronary intervention, restricted to trials enrolling both chronic and acute coronary syndrome populations. BARC, Bleeding Academic Research Consortium; CI, confidence interval; DAPT, dual antiplatelet therapy; MACE, major adverse cardiovascular events; NACE, net adverse clinical events; P2Y12i, P2Y12 receptor inhibitor; RR, risk ratio; SUCRA, surface under the cumulative ranking curve. [file 13293_2026_903_MOESM34_ESM.tif]

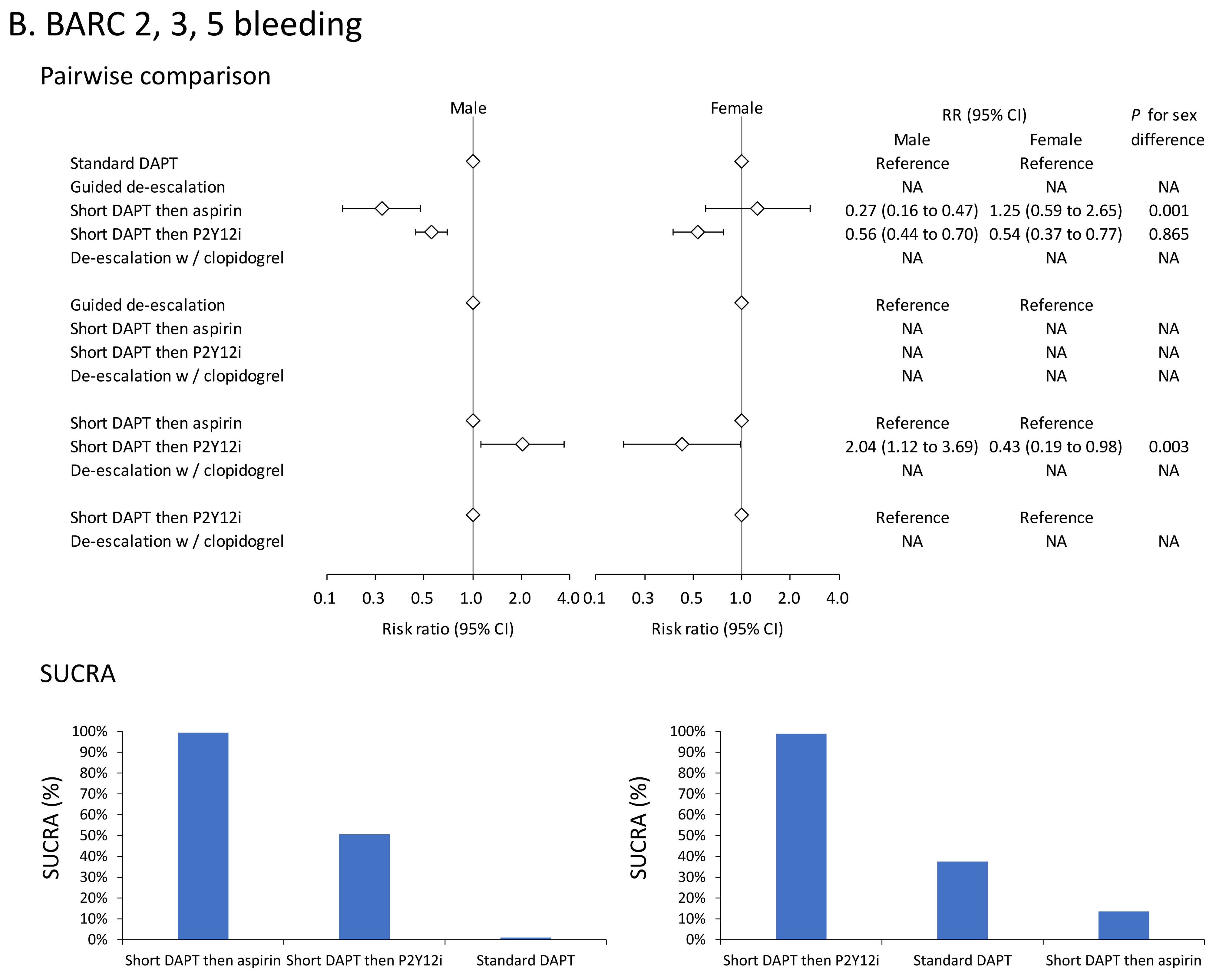

Supplement: Supplementary file 35 — Supplementary Material 35 [file 13293_2026_903_MOESM35_ESM.tif]

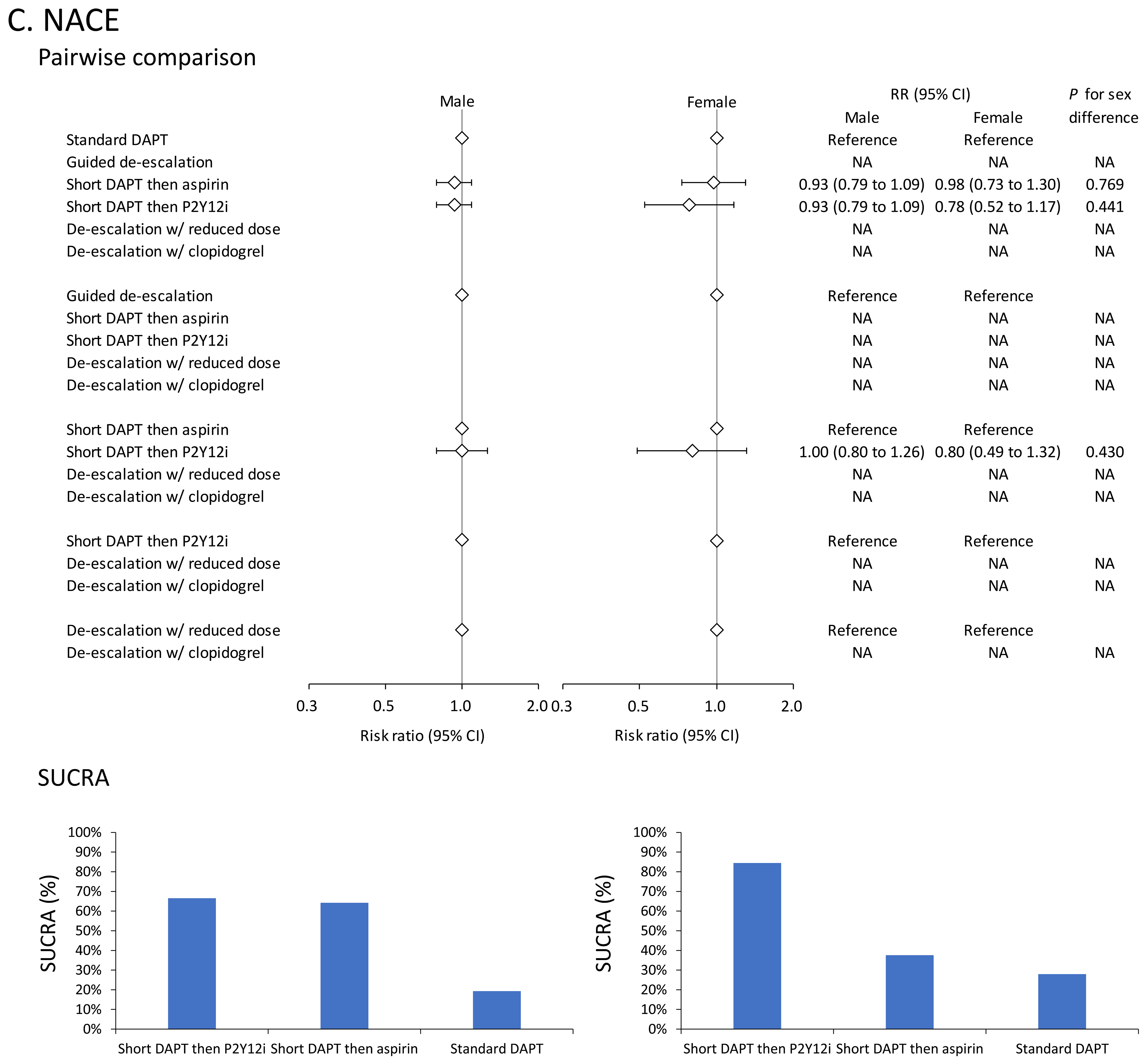

Supplement: Supplementary file 36 — Supplementary Material 36 [file 13293_2026_903_MOESM36_ESM.tif]

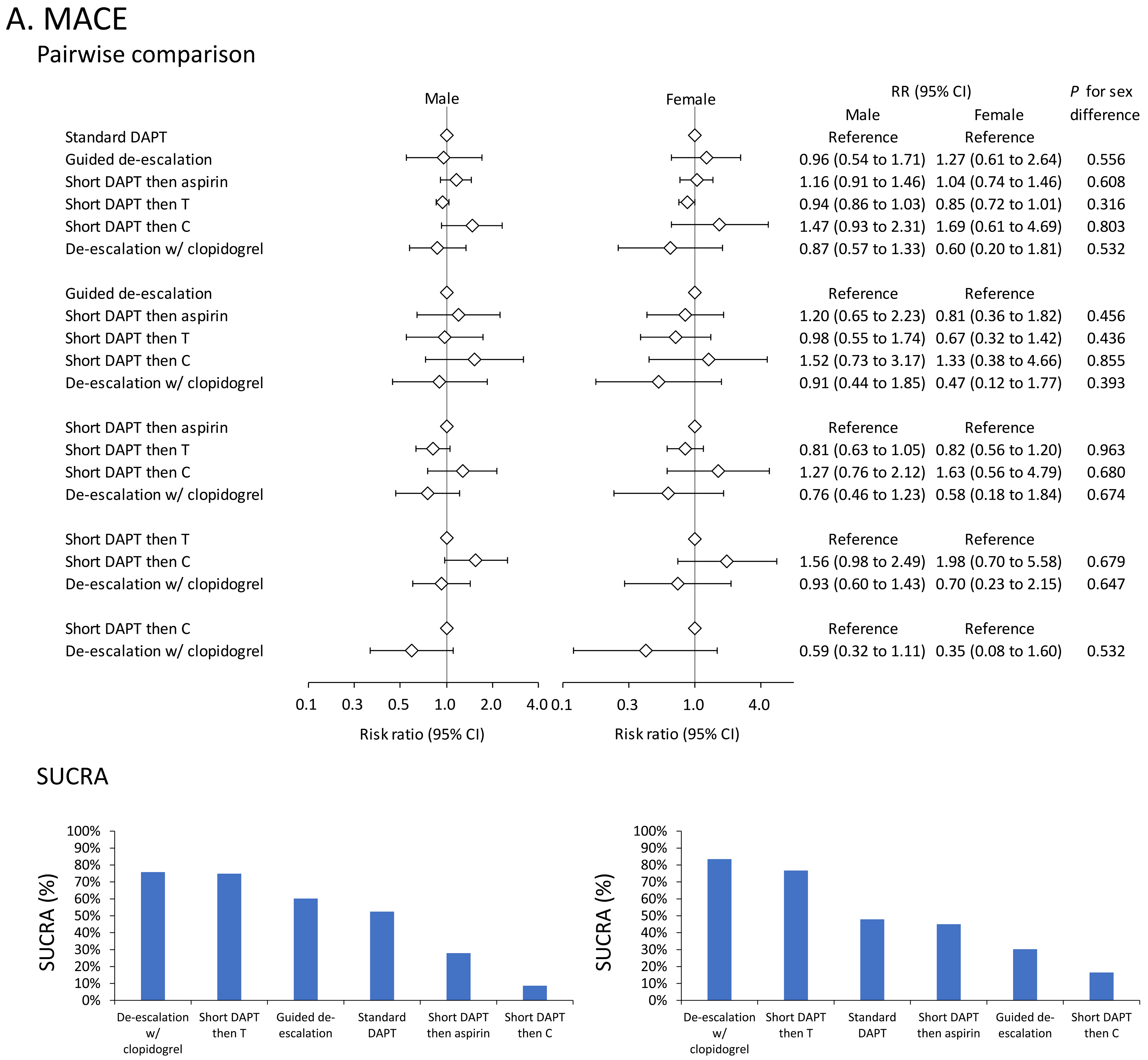

Supplement: Supplementary file 37 — Supplementary Material 37: Fig. S12. Forest plot and SUCRA of the network meta-analysis of MACE (A), BARC 2, 3, 5 bleeding (B), and NACE (C) among patients receiving different dual antiplatelet therapy strategies following percutaneous coronary intervention, using a seven-node network in which the short DAPT followed by P2Y12 inhibitor monotherapy category was sub-divided into potent (ticagrelor-based) and clopidogrel-based strategies. BARC, Bleeding Academic Research Consortium; CI, confidence interval; DAPT, dual antiplatelet therapy; MACE, major adverse cardiovascular events; NACE, net adverse clinical events; P2Y12i, P2Y12 receptor inhibitor; RR, risk ratio; SUCRA, surface under the cumulative ranking curve. [file 13293_2026_903_MOESM37_ESM.tif]

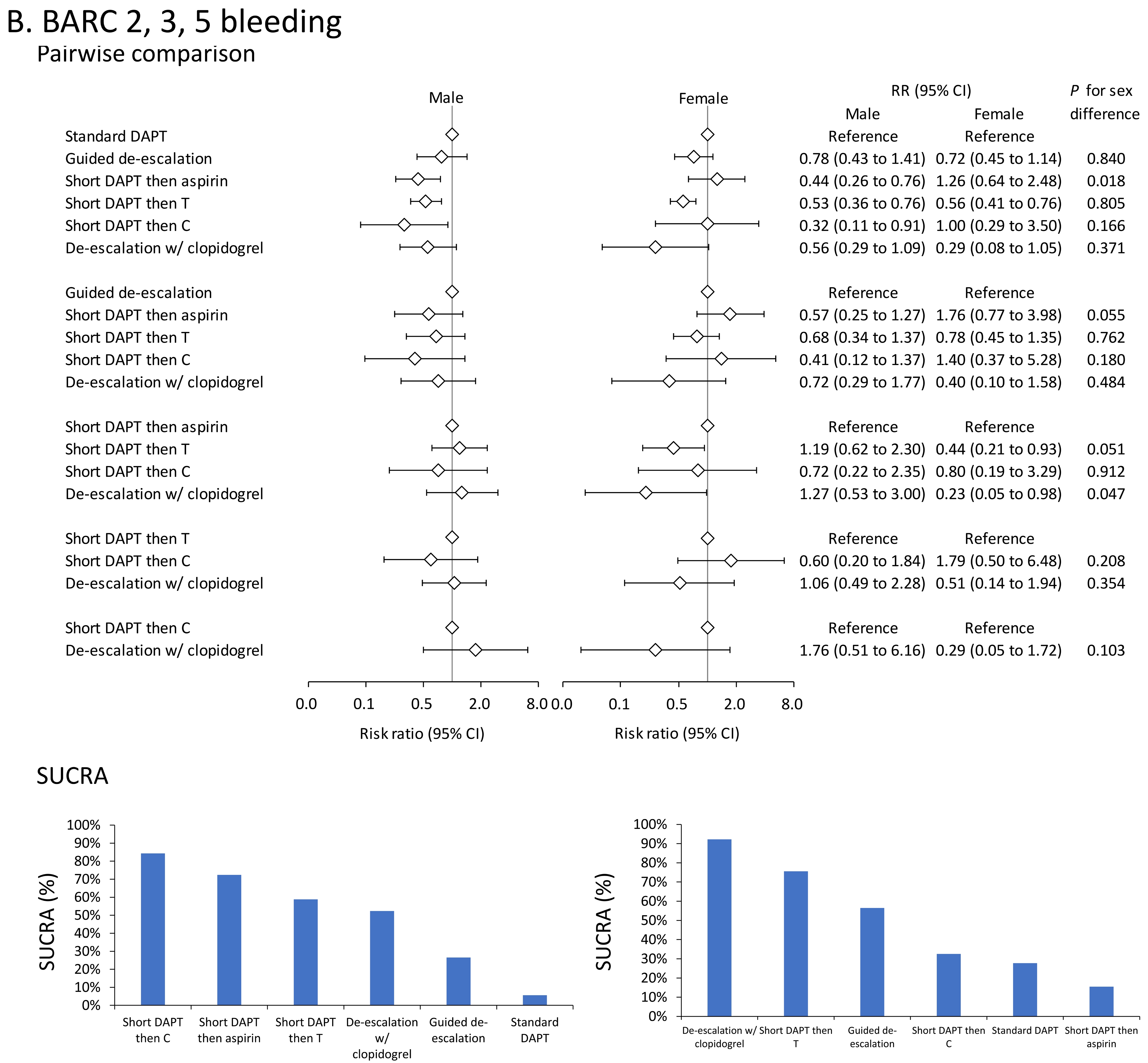

Supplement: Supplementary file 38 — Supplementary Material 38 [file 13293_2026_903_MOESM38_ESM.tif]

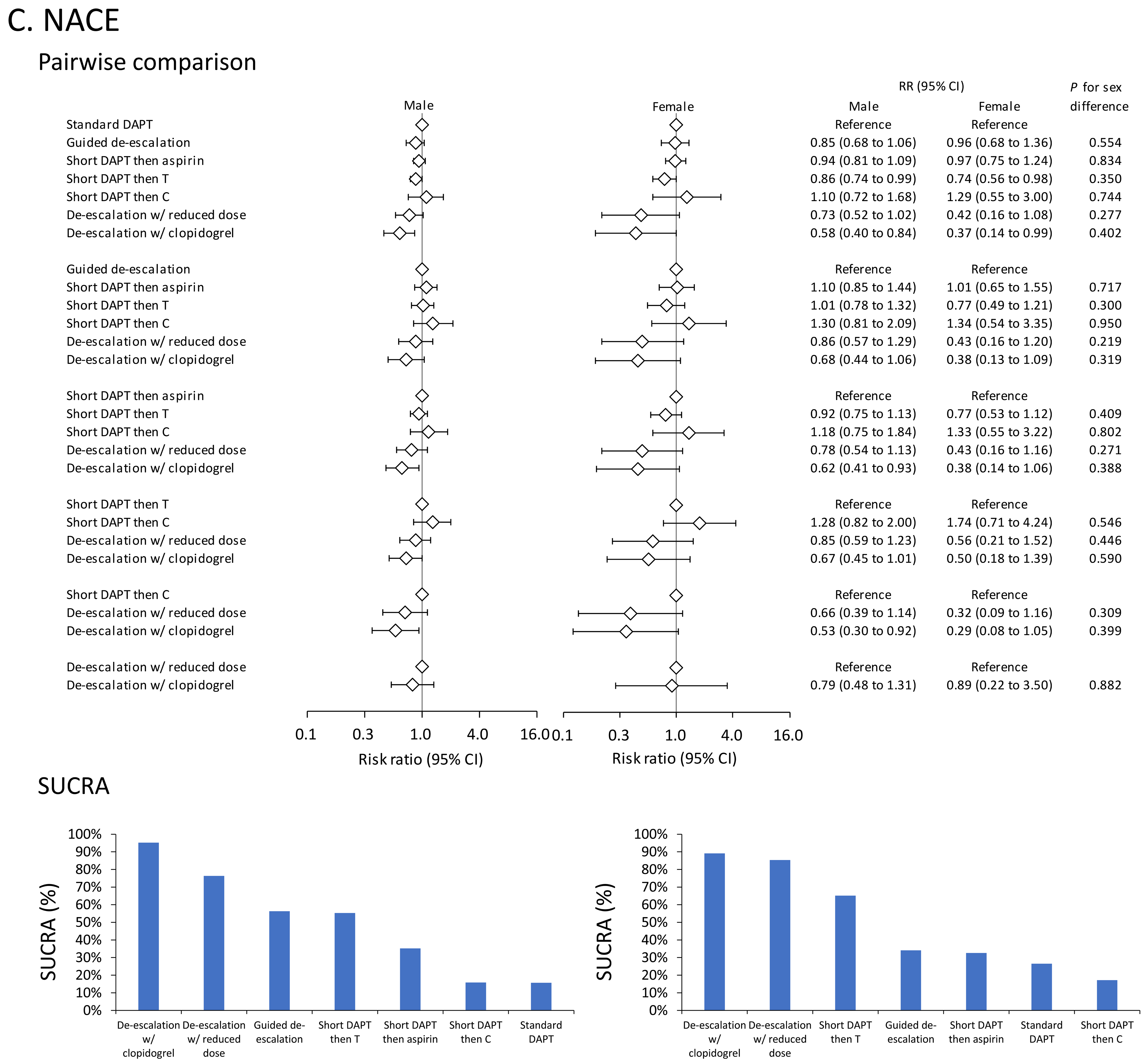

Supplement: Supplementary file 39 — Supplementary Material 39 [file 13293_2026_903_MOESM39_ESM.tif]
